# Multiscale machine learning molecular mechanics for mechanism and stereoselectivity of Diels-Alderase catalysis

**DOI:** 10.1038/s41467-026-72904-9

**Published:** 2026-05-13

**Authors:** Xujian Wang, Haocheng Tang, Xiongwu Wu, Bernard R. Brooks, Junmei Wang, Wan-Lu Li

**Affiliations:** 1 Aiiso Yufeng Li Family Department of Chemical and Nano Engineering, University of California San Diego, San Diego, CA USA; 2 Department of Pharmaceutical Sciences and Computational Chemical Genomics Screening Center, School of Pharmacy, University of Pittsburgh, Pittsburgh, PA USA; 3 Department of Computational and Systems Biology, School of Medicine, University of Pittsburgh, Pittsburgh, PA USA; 4 Laboratory of Computation Biology, National Heart, Lung and Blood Institute, National Institutes of Health, Bethesda, MD USA; 5 Program of Materials Science and Engineering, University of California San Diego, San Diego, CA USA

**Keywords:** Computational chemistry, Method development

## Abstract

Enzymes catalyze complex chemical transformations with remarkable efficiency and selectivity, yet their atomistic mechanisms remain challenging to capture because conventional simulations trade accuracy for efficiency. Here we introduce a reactive machine learning/molecular mechanics (ML/MM) framework that bridges quantum chemistry with long-timescale sampling, enabling direct exploration of enzymatic transition states and free-energy landscapes. Coupled with metadynamics, this approach achieves nanosecond sampling of bond-forming reactions and quantitatively predicts activation barriers, mutational effects, and stereoselectivity. Applied to Diels-Alderases, the framework not only reproduces experimental activity and *endo*/*exo* preferences with sub-kcal mol^−1^ accuracy but also uncovers how pathway dynamics and local electrostatics preorganize substrates for selective outcomes. By uniting reactivity, conformational dynamics, and predictive power, this work establishes reactive ML/MM as a broadly applicable strategy for mechanistic enzymology and a foundation for the rational design of new biocatalysts.

## Abstract (web summary)

Enzymes catalyze complex reactions with high efficiency and selectivity, but resolving their atomistic mechanisms remains challenging due to limitations of conventional simulation approaches. Here, the authors develop a reactive ML/MM framework combined with enhanced sampling that enables the exploration of enzymatic reactions, reproduces key experimental observables, and provides insight into the roles of dynamics and electrostatics in determining selectivity.

## Introduction

**Fig. 1:**
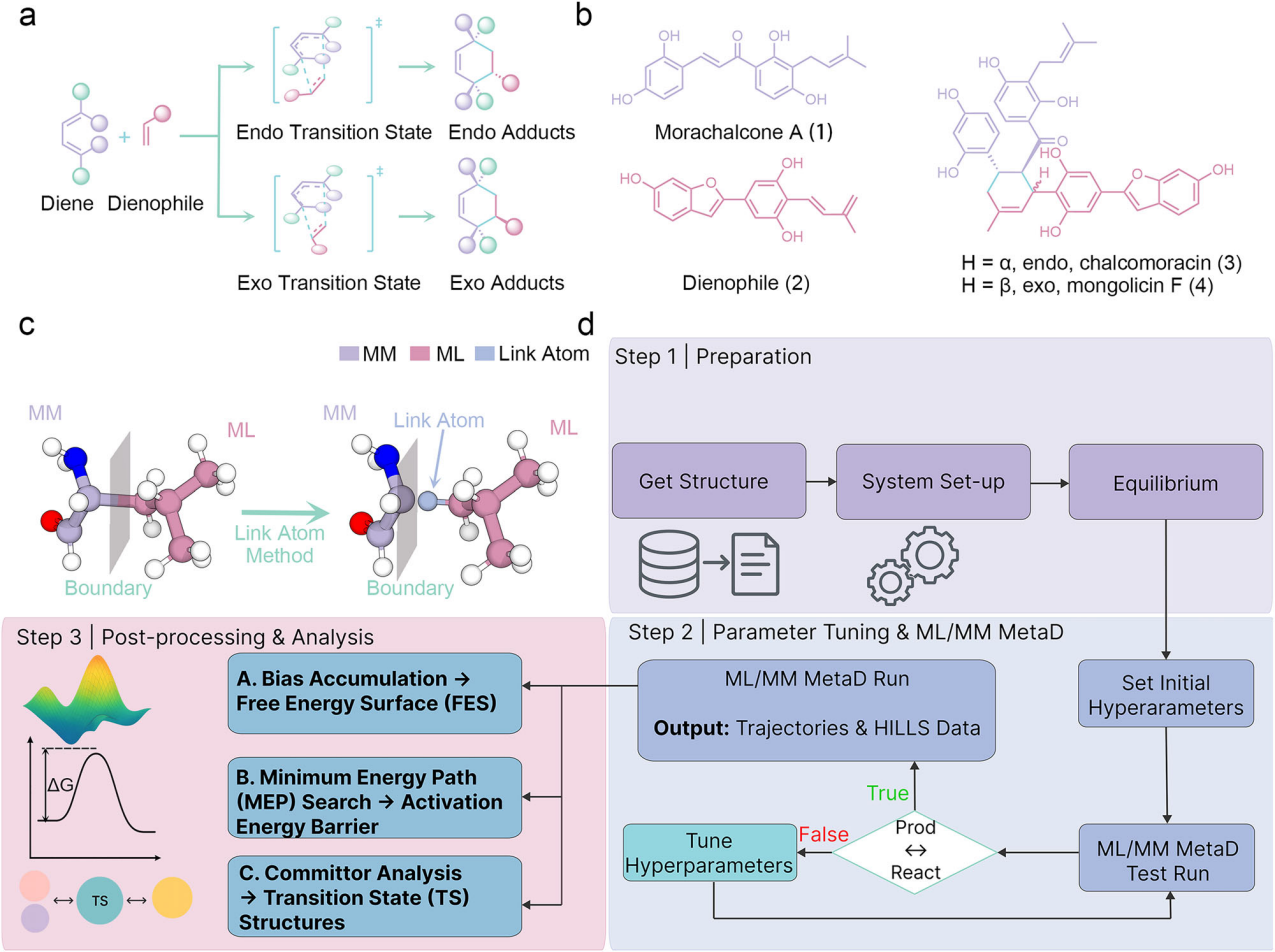
D–A reaction and reactive ML/MM MetaD workflow. **a** Two competing pathways of the D–A reaction affording the *exo* and *endo* products; ‡ denotes the transition state. **b** The natural substrate and catalytic products of the MaDA enzyme family. **c** Schematic representation of the link-atom strategy within the machine-learning/molecular-mechanics (ML/MM) framework; purple, pink and blue spheres represent MM, ML and link-atom regions, respectively. **d** The reactive ML/MM metadynamics (MetaD) protocol, encompassing system setup (blue), hyperparameter optimization (green) and subsequent post-simulation analyses (pink). Green and red text in the decision node indicate convergence (true) and non-convergence (false), respectively.

The Diels–Alder (D–A) reaction, a [4+2] cycloaddition between a conjugated diene and a dienophile, is a cornerstone of synthetic chemistry because of its efficiency in constructing complex cyclic structures. Recent studies have shown that nature also exploits this mode of reactivity, as numerous enzymes catalyzing D–A-like transformations have been discovered in biosynthetic pathways^1–5^. Among them, SpnF was the first stand-alone intramolecular [4 + 2] cyclase identified^6^, broadening the enzymatic repertoire of natural D–A reactions. More recently, Gao et al. reported the first intermolecular Diels-Alderase (MaDA) from *Morus alba*, which generates a methylcyclohexene skeleton, a motif prevalent in bioactive compounds of pharmaceutical interest^1,2^. Despite its utility, MaDA exhibits poor enantioselectivity, limiting its broader biocatalytic potential (Fig. 1a). Protein engineering has since yielded two stereoselective variants: MaDA-1, which favors the *endo* pathway to produce chalcomoracin (**3**) from morachalcone A (**1**) and a dienophile (**2**), and MaDA-3, which instead directs the reaction along the *exo* pathway to yield mongolicin (**4**) (Fig. 1b). These complementary variants provide a compelling model system for dissecting the molecular basis of enzymatic stereocontrol and highlighting the challenges of controlling selectivity in complex enzymatic environments.

To move beyond trial-and-error experimental mutagenesis, computational methods provide atomic-level insights for rational enzyme design^7,8^. Molecular dynamics (MD) and quantum mechanics (QM) are central tools: conventional molecular dynamics (cMD) relies on fixed force fields that cannot describe bond breaking, while QM captures such events but is too costly for large systems. To balance accuracy and efficiency, Warshel and Levitt introduced the hybrid QM/MM framework^9^, which has been widely applied to the studies of mechanisms and free-energy landscapes, though its scope is limited by computational cost. Recent advances in machine learning interatomic potentials (MLIPs) reproduce QM-level accuracy at greatly reduced cost^10–14^. Building on this, the ML/MM framework replaces the QM region with MLIPs, achieving both efficiency and accuracy. In recent years, ML/MM has been implemented across several mainstream MD software platforms, including GROMOS^15,16^, GENESIS^17^, and AMBER^18–21^. A central challenge common to all ML/MM implementations is the treatment of the coupling between the ML and MM subsystems. The simplest approach, mechanical embedding (ME), evaluates the ML region in vacuo and adds classical non-bonded interactions between subsystems; this strategy has been employed in early AMBER-based ML/MM work^18,20^. However, ME ignores the polarization of the ML region by the MM environment. Therefore a more advanced polarization-corrected mechanical embedding (PCME) schemes have been developed by Semelak et al.^21^; subsequently, Zinovjev et al.^19,22^ introduced an electrostatic embedding. Beyond the embedding scheme, progress in ML/MM spans long-range electrostatic treatments, model architectures, and coupling strategies; comprehensive accounts of these developments can be found in recent reviews^23–25^.

To address this limitation, we draw on decades of QM/MM experience with boundary schemes including hybrid orbitals^26^, buffer methods^27^, and pseudobonds^28^, and adopt the link-atom method^29^ (Fig. 1c), the most widely used approach^30^, to incorporate active-site residues into the ML region and thereby extend reactive ML/MM to the full breadth of enzymatic mechanisms. In the link-atom scheme, a hydrogen capping atom is inserted along each severed covalent bond at every MD step, saturating the dangling valence of the boundary atom and providing the MLIP descriptor with a chemically complete local environment. A single link-atom module can interface with MLIPs built on fundamentally different representation paradigms, including ANI^11,31^, MACE^14,32^, AIMNet2^12,13,33,34^, SpookyNet^35^, and EANN^36–38^, providing a unified and backend-agnostic codebase. Combined with state-of-the-art universal reactive potentials such as ANI-1xnr^31^ and AIMNet2-NSE^34^ that require no system-specific training, and with an interface to PLUMED^39,40^ for enhanced sampling methods such as metadynamics, this design removes the key barriers that have historically limited the accessibility of reactive ML/MM simulations.

Here, we present a reactive ML/MM framework implemented within AMBER^41,42^ that incorporates the link-atom boundary treatment, seamlessly coupling reactive MLIPs with molecular mechanics environments. We validate the framework on the intermolecular Diels–Alderase MaDA, where ML/MM metadynamics reproduces the reaction barrier in agreement with both DFT calculations and experimental data^1,2^. Committor and frequency analyses confirm the located transition states, and the simulations reveal an asynchronous mechanism consistent with prior computational and experimental evidence^1,2^. We further demonstrate that the framework captures mutational effects on enantioselectivity across engineered MaDA variants and extends to diverse substrates, reproducing differences in reaction barriers and stereochemical outcomes in agreement with experimental observations^1^. Altogether, this work establishes a versatile ML/MM platform for resolving subtle effects of enantiomers, mutations, and substrates, providing a practical tool for rational enzyme design.

## Results

### Link-atom method for the ML/MM approach

Accurate multiscale simulations require proper treatment of covalent bonds crossing the ML/MM boundary. A straightforward partition can leave a dangling valence in the ML region, perturbing the electronic structure and introducing significant errors^30^. To address this, we adapt the link-atom construction from QM/MM to ML/MM. For a bond split between atoms MLL (ML side) and MML (MM side), a link atom *L* is added to cap the ML valence. *L* introduces no additional degrees of freedom; its position is constrained along the MLL-MML bond vector and updated on the fly from their coordinates. Let **R**_MLL_ and **R**_MML_ be their Cartesian coordinates, and define **r** = **R**_MML_ − **R**_MLL_, *u* = **r**/∥**r**∥. The link-atom position is then

1 $${{{{\bf{R}}}}}_{L}\,=\,{{{{\bf{R}}}}}_{{{{\rm{MLL}}}}}\,+\,d\,u\,=\,{{{{\bf{R}}}}}_{{{{\rm{MLL}}}}}\,+\,d\,\frac{{{{{\bf{R}}}}}_{{{{\rm{MML}}}}}-{{{{\bf{R}}}}}_{{{{\rm{MLL}}}}}}{\parallel {{{{\bf{R}}}}}_{{{{\rm{MML}}}}}-{{{{\bf{R}}}}}_{{{{\rm{MLL}}}}}\parallel },$$

where *d* is an equilibrium capping distance. Unless otherwise specified, we use a hydrogen link atom with *d* = 1.09 Å (the equilibrium C–H bond length in methyl). Because *L* is an auxiliary “ghost” atom used only to make the ML potential well-defined, it is not integrated as an independent particle; instead, **R**_*L*_ is deterministically reconstructed at each step from Eq. (1).

The ML energy *E*(**R**_ML_, **R**_*L*_) depends on the ML-region coordinates and on **R**_*L*_. The force on the link atom is

2 $${{{{\bf{F}}}}}_{L}\,=\,-\,\frac{\partial E}{\partial {{{{\bf{R}}}}}_{L}}.$$

To ensure energy-consistent coupling and momentum conservation, the contribution **F**_*L*_ is redistributed to the boundary atoms by the chain rule, using the geometric dependence of **R**_*L*_ on **R**_MLL_ and **R**_MML_:

3 $${{{{\bf{F}}}}}_{{{{\rm{MLL}}}}}^{{{{\rm{red}}}}}\,=\,{{{{\bf{F}}}}}_{L}\,\frac{\partial {{{{\bf{R}}}}}_{L}}{\partial {{{{\bf{R}}}}}_{{{{\rm{MLL}}}}}},$$

4 $${{{{\bf{F}}}}}_{{{{\rm{MML}}}}}^{{{{\rm{red}}}}}\,=\,{{{{\bf{F}}}}}_{L}\,\frac{\partial {{{{\bf{R}}}}}_{L}}{\partial {{{{\bf{R}}}}}_{{{{\rm{MML}}}}}}.$$

Here ${{{{\bf{F}}}}}_{{{{\rm{MLL}}}}}^{{{{\rm{red}}}}}$ and ${{{{\bf{F}}}}}_{{{{\rm{MML}}}}}^{{{{\rm{red}}}}}$ are the portions of the link-atom force that are accumulated on the two boundary atoms, and their sum reproduces the virtual work associated with **F**_*L*_.

The required Jacobians follow directly from Eq. (1). Let **I** denote the 3 × 3 identity and set **r** = **R**_MML_ − **R**_MLL_. Using ∂*u*/∂**r** = ∥**r**∥^−1^**I** − ∥**r**∥^−3^ **rr**^⊤^, together with ∂**r**/∂**R**_MLL_ = − **I** and ∂**r**/∂**R**_MML_ = **I**, we obtain:

5 $$\frac{\partial {{{{\bf{R}}}}}_{L}}{\partial {{{{\bf{R}}}}}_{{{{\rm{MLL}}}}}}={{{\bf{I}}}}\,-\,d\,\frac{{{{\bf{I}}}}}{\parallel {{{\bf{r}}}}\parallel }\,+\,d\,\frac{{{{\bf{r}}}}{{{{\bf{r}}}}}^{\top }}{\parallel {{{\bf{r}}}}{\parallel }^{3}},$$

6 $$\frac{\partial {{{{\bf{R}}}}}_{L}}{\partial {{{{\bf{R}}}}}_{{{{\rm{MML}}}}}}=d\,\frac{{{{\bf{I}}}}}{\parallel {{{\bf{r}}}}\parallel }\,-\,d\,\frac{{{{\bf{r}}}}{{{{\bf{r}}}}}^{\top }}{\parallel {{{\bf{r}}}}{\parallel }^{3}}.$$

These expressions are inserted into Eqs. (3 and 4) to compute the redistributed forces at each step. In practice, this construction (i) avoids introducing extra dynamical variables, (ii) preserves the correct projection of forces along and perpendicular to the cut bond, and (iii) stabilizes the ML description of the boundary without contaminating the MM region.

### Energy partitioning at the ML/MM boundary

With the link-atom construction defined, we next specify the total energy decomposition to avoid double counting between the ML potential and the MM force field. We denote the atoms treated by the ML potential (capped by the link atom *L* attached to MLL) as ML and the remaining atoms including the boundary atom MML as MM. Unless stated otherwise, *L* is used only as an auxiliary coordinate within the ML energy and coupling terms.

All bonded interactions (bonds, angles, dihedrals) entirely within the ML region are excluded from MM calculations, since they are already represented by the ML potential. Only mixed terms that cross the ML/MM boundary and purely MM terms are retained:

7 $${E}_{{{{\rm{ML}}}}-{{{\rm{MM}}}}}^{{{{\rm{bonded}}}}}=	{\sum}_{{(i,j)\in {{{\rm{bond}}}}}\atop {\{i,j\}\not\subset {{{\rm{ML}}}}}}{V}_{{{{\rm{bond}}}}}(i,j)\,+\,{\sum}_{{(i,j,k)\in {{{\rm{angle}}}}}\atop {\{i,j,k\}\not\subset {{{\rm{ML}}}}}}{V}_{{{{\rm{angle}}}}}(i,j,k)\\ 	+{\sum}_{{(i,j,k,l)\in {{{\rm{dihedral}}}}}\atop {\{i,j,k,l\}\not\subset {{{\rm{ML}}}}}}{V}_{{{{\rm{dihedral}}}}}(i,j,k,l).$$

In particular, interactions involving MML and its MM neighbors are included, whereas those fully within the ML region (including the capped valence at MLL) are excluded from the MM Hamiltonian.

Nonbonded van der Waals (vdW) interactions between ML and MM atoms are evaluated at the MM level. While the interactions between any two ML region atoms including the auxiliary *L*, are omitted to avoid double counting with the ML energy. Denoting ${{{{\bf{R}}}}}_{i}^{{{{\rm{MM}}}}}$ and ${{{{\bf{R}}}}}_{j}^{{{{\rm{ML}}}}}$ as MM and ML coordinates, respectively, the interaction takes the standard Lennard–Jones 12-6 potential form:

8 $${E}_{{{{\rm{ML}}}}-{{{\rm{MM}}}}}^{{{{\rm{vdW}}}}}=\,\sum_ {{i\in {{{\rm{MM}}}}}\atop {j\in {{{\rm{ML}}}}}}{\epsilon }_{ij}\,\left[{\left(\frac{{A}_{ij}}{\left\Vert {{{{\bf{R}}}}}_{i}^{{{{\rm{MM}}}}}-{{{{\bf{R}}}}}_{j}^{{{{\rm{ML}}}}}\right\Vert }\right)}^{12}-{\left(\frac{{B}_{ij}}{\left\Vert {{{{\bf{R}}}}}_{i}^{{{{\rm{MM}}}}}-{{{{\bf{R}}}}}_{j}^{{{{\rm{ML}}}}}\right\Vert }\right)}^{6}\right],$$

where *A*_*ij*_, *B*_*ij*_ and *ϵ*_*ij*_ are given by standard mixing rules and depend on the atom types of *i* and *j*.

For Coulomb coupling we adopt an e nergy-consistent protocol following Field and co-workers^43^, combined with a charge-balancing scheme similar to Walker and colleagues for stable QM/MM embedding^30^. The ML region is assigned an integer total charge; if its nominal charge is non-integer, a user-specified integer is enforced by redistributing small compensating charges onto MM atoms to maintain overall neutrality (or the desired total charge). When calculating the coupling energy, purely ML-ML electrostatics (including pairs involving *L*) are excluded, and ML-MM interactions are evaluated at the MM level. For the MM boundary atom MML, its coordinate is replaced by the link-atom position **R**_*L*_ when computing distances to other MM atoms, thereby reducing artifacts from cutting a polar bond at the boundary. The resulting coupling term is:

9 $${E}_{{{{\rm{ML}}}}-{{{\rm{MM}}}}}^{{{{\rm{elec}}}}}=\,{\sum}_{{i\in {{{\rm{MM}}}},\,i\ne {{{\rm{MML}}}}}\atop {j\in {{{\rm{ML}}}}}}\frac{{q}_{i}\,{q}_{j}}{\left\Vert {{{{\bf{R}}}}}_{i}^{{{{\rm{MM}}}}}-{{{{\bf{R}}}}}_{j}^{{{{\rm{ML}}}}}\right\Vert }\,+\,{\sum}_{j\in {{{\rm{MM}}}},\,j\ne {{{\rm{MML}}}}}\frac{{q}_{{{{\rm{MML}}}}}\,{q}_{j}}{\left\Vert {{{{\bf{R}}}}}_{L}-{{{{\bf{R}}}}}_{j}^{{{{\rm{MM}}}}}\right\Vert },$$

where *q*_*i*_ and *q*_*j*_ are the MM partial charges. This formulation avoids double counting with the ML energy, ensures a consistent electrostatic boundary, and improves numerical stability in practice.

### Probe the MaDA Diels-Alderases with ML/MM MetaD

ANI-1xnr was chosen to model the MaDA-catalyzed D–A reaction because, unlike many general-purpose MLIPs, it is specifically curated to describe covalent bond formation and breaking along reaction pathways. Two-dimensional PES scans along the endo and exo forming-bond coordinates at the DFT and ANI-1xnr levels confirm close agreement in reaction pathway geometry and energetics, with only minor deviations in non-reactive regions (Fig. S1). Systematic benchmarking against *ω*B97X/6-31G* QM/MM across ML regions spanning 84–822 atoms demonstrates speedups of 690–955 ×, with throughput independent of ML region size and negligible link-atom overhead (Tables S2 and S3).

**Fig. 2:**
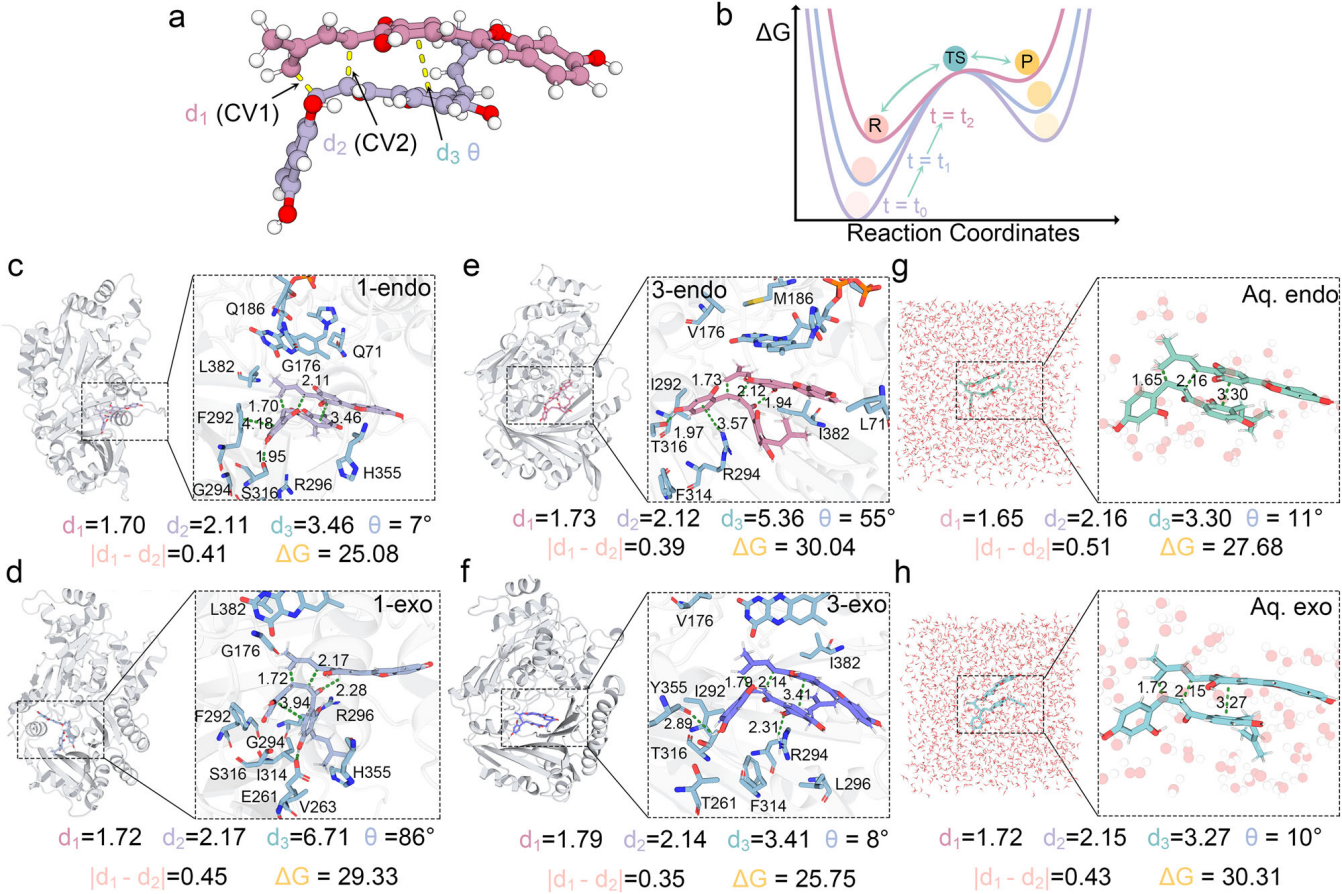
Transition-state structures and energetics of the D–A reaction. **a** Substrate geometry showing the two CV *d*_1_ (CV1) and *d*_2_ (CV2), the auxiliary distance *d*_3_ and the dihedral angle *θ*. **b** Schematic free-energy profiles along the reaction coordinate at successive metadynamics time points (*t*_0_-*t*_2_); R, TS and P denote reactant, transition state and product, respectively. Purple, blue and yellow curves correspond to progressive bias-potential filling. **c**–**f** Representative transition-state snapshots for the *endo* (**c**, **e**) and *exo* (**d**, **f**) pathways of substrates **1** (**c**, **d**) and **3** (**e**, **f**) within the enzyme active site. Key interatomic distances are given in Å; residues within 4 Å of the substrate are shown as blue sticks. **g**, **h** Corresponding transition-state structures for the *endo* (**g**) and *exo* (**h**) pathways in aqueous solution; water molecules are depicted as red spheres. In all panels, *d*_1_, *d*_2_, *d*_3_, *θ*, ∣*d*_1_ − *d*_2_∣ and activation free energies (*ΔG*^‡^, kcal mol^−1^) are reported below each structure. Bars indicate the mean; error bars represent one standard deviation (*N* = 5 independent simulation replicates.

MetaD was integrated to overcome the challenge of rare-event sampling, accelerating transitions along well-defined collective variables (CVs). For the intramolecular D–A cycloaddition, as shown in Fig. 2a, we employed two CVs corresponding to the forming C–C bonds, a choice that accounts for the asynchronicity of this reaction^2^. Their suitability was validated by path-CV steered MD simulations, which revealed a sequential mechanism in which CV1 is elaborated first, followed by CV2, and finally the products dissociate into two substrates (Fig. S5). To determine the minimal ML region sufficient for converged barriers, we performed MetaD simulations of MaDA-3 with four progressively expanded ML regions, from substrate only (84 atoms) to a second coordination shell (833 atoms). The activation barrier converges upon inclusion of the catalysis-related residues identified in prior work^1^ (222 atoms, 12 residues; 30.0 ± 1.7 kcal mol^−1^), with no statistically significant change upon further expansion (Fig. S5), and this region was therefore adopted for all production simulations.

Using these CVs and optimized MetaD parameters, we investigated MaDA-3, MaDA-1, and the aqueous reaction of substrates **1** and **2**. The predicted activation free energies (*ΔG*^‡^) reproduced the relative trends observed in DFT and experiment, though absolute values obtained with ANI-1xnr exhibited a positive offset of approximately 5 kcal mol^−1^ compared to DFT cluster model results (and approximately 7–9 kcal mol^−1^ compared to experimental values, which themselves differ from DFT estimates by 1–4 kcal mol^−1^^1,2^). Encouragingly, ML/MM MetaD simulations performed with AIMNet2-NSE yielded barriers substantially closer to the DFT reference, reducing the mean unsigned deviation to approximately 2–3 kcal mol^−1^ across all six pathways (Table S5), suggesting that more advanced reactive universal potentials hold considerable promise for further improving quantitative accuracy within this framework. Such deviations may also reflect (i) the generalization limits of MLIPs trained on finite DFT datasets, (ii) incomplete treatment of long-range interactions, and (iii) the use of mechanical rather than electrostatic embedding. However, the traditional methods, like theozyme model would also leads to energy difference up to 5 kcal mol^−1^^8,44–47^, but for a different reason: their oversimplified treatment of the protein environment inherently neglects conformational ensembles, electrostatics, and mutational effects. By contrast, our ML/MM MetaD framework systematically incorporates protein dynamics and explicit environment, enabling reliable prediction of relative barriers. More importantly, it correctly capture enantioselectivity trend inaccessible to static theozyme models: MaDA-3 favors the *exo* product (**4**), MaDA-1 favors the *endo* product (**3**), and aqueous solution favors the *endo* pathway. The computed relative activation free energy (*ΔΔG*) values are in excellent agreement with the DFT results (Table S5), supporting its robustness as a more meaningful metric than absolute *ΔG*^‡^.

Importantly, despite the systematic offset in *ΔG*^‡^, relative barriers are reliably captured by error cancellation. Our ML/MM MetaD framework correctly distinguishes enantioselective outcomes: MaDA-1 favors the *endo* product (**3**), whereas MaDA-3 favors the *exo* product (**4**). In aqueous solution, where the environment is more disordered and steric constraints are reduced, the *endo* pathway is thermodynamically preferred. In this case, our method yields *ΔΔG* = − 2.63 kcal mol^−1^, in close agreement with the DFT estimate of −2.8 kcal mol^−1^ (Table S5). Likewise, the difference between MaDA-3 *exo* and MaDA-1 *endo* is reproduced with high fidelity (ML/MM: 0.67 kcal mol^−1^; DFT: 0.5 kcal mol^−1^, Table S5). Taken together, these results indicate that *ΔΔG* is a more reliable and mechanistically meaningful metric than absolute *ΔG*^‡^ within this protocol.

Beyond activation free energies, ML/MM MetaD enables direct and reliable identification of TS structures, a key requirement for structure-based enzyme design. Its computational efficiency supports nanosecond-scale simulations, during which multiple reactive events were sampled (Fig. 2b), providing access to well-sampled ensembles of TS configurations, in contrast to the isolated snapshots typically obtained from static QM or theozyme models. Committor and vibrational frequency analyses (Fig. S8) confirmed the validity of the generated configurations. To characterize TS geometry, we defined and evaluated four geometric descriptors shown in Fig. 2a: the two forming C–C distances (*d*_1_, *d*_2_), the benzene-benzene centroid distance (*d*_3_), and the dihedral angle between the ring planes (*θ*). Interestingly, *d*_1_ and *d*_2_ varied only slightly (1.65–1.79 Å and 2.11–2.17 Å), making them less informative. In contrast, *d*_3_ and *θ* are better geometric descriptors for distinguishing favored enantiomers in MaDA-1 and MaDA-3 (Fig. 2C–F), as they closely resembled aqueous-phase TS structures (Fig. 2G, H). Favored TSs consistently displayed stacked benzene rings, stabilized by *π*-*π* interactions. By contrast, in disfavored TS structures (Fig. 2D, E), this stacking arrangement was disrupted, highlighting a key structural determinant underlying the distinct enantioselective outcomes of MaDA-1 and MaDA-3. Interestingly, disfavored TSs often attempted to compensate through alternative interactions. For example, in the MaDA-1 *exo* pathway, one benzene ring distorted the backbone of substrate **1** to establish a weak hydrogen bond with E261 and another *π*-*π* stacking interaction (Fig. 2d). However, this could not compensate for the loss of stabilization normally provided by F292 and S316 (Fig. 2c). A similar pattern was observed in MaDA-3, where alternative contacts arose but remained insufficient. Importantly, the model was able to capture higher-order weak interactions, such as multipole effects, *π*-*π* stacking, and *π*-cation interactions, which are typically not well described by traditional force fields due to their limited functional form^48,49^. This finding highlights the potential of ML/MM approaches in accurately describing delicate electrostatic interactions. It also demonstrate that ML/MM MetaD framework not only identifies preferred TS geometries but also elucidates the compensatory interactions that arise in disfavored enantiomeric pathways, which prove insufficient to overcome their higher free-energy cost.

Apart from stereoselectivity, the MaDA-catalyzed D–A reaction exhibits a pronounced asynchronicity in bond formation, with the difference between the two forming C–C bonds (*d*_1_–*d*_2_) spanning 0.35 to 0.51 Å. This agrees with previous DFT studies and kinetic isotope effect (KIE) experiments on substrate analogs^2^, which together established asynchronicity as a key mechanistic signature of D–A enzymology. Motivated by these findings, we tested whether ML/MM MetaD could emulate KIEs computationally. By substituting hydrogen (1.008 amu) with deuterium (2.014 amu), our simulations reproduced experimental KIE trends (Fig. S6). These results demonstrate that ML/MM MetaD not only recapitulates stereoselectivity and barrier heights but also extends to subtle kinetic observables such as isotope effects, underscoring its potential as a broadly predictive tool for enzymatic reaction mechanisms.

### Investigation of mutation effects on activity and stereoselectivity

**Fig. 3:**
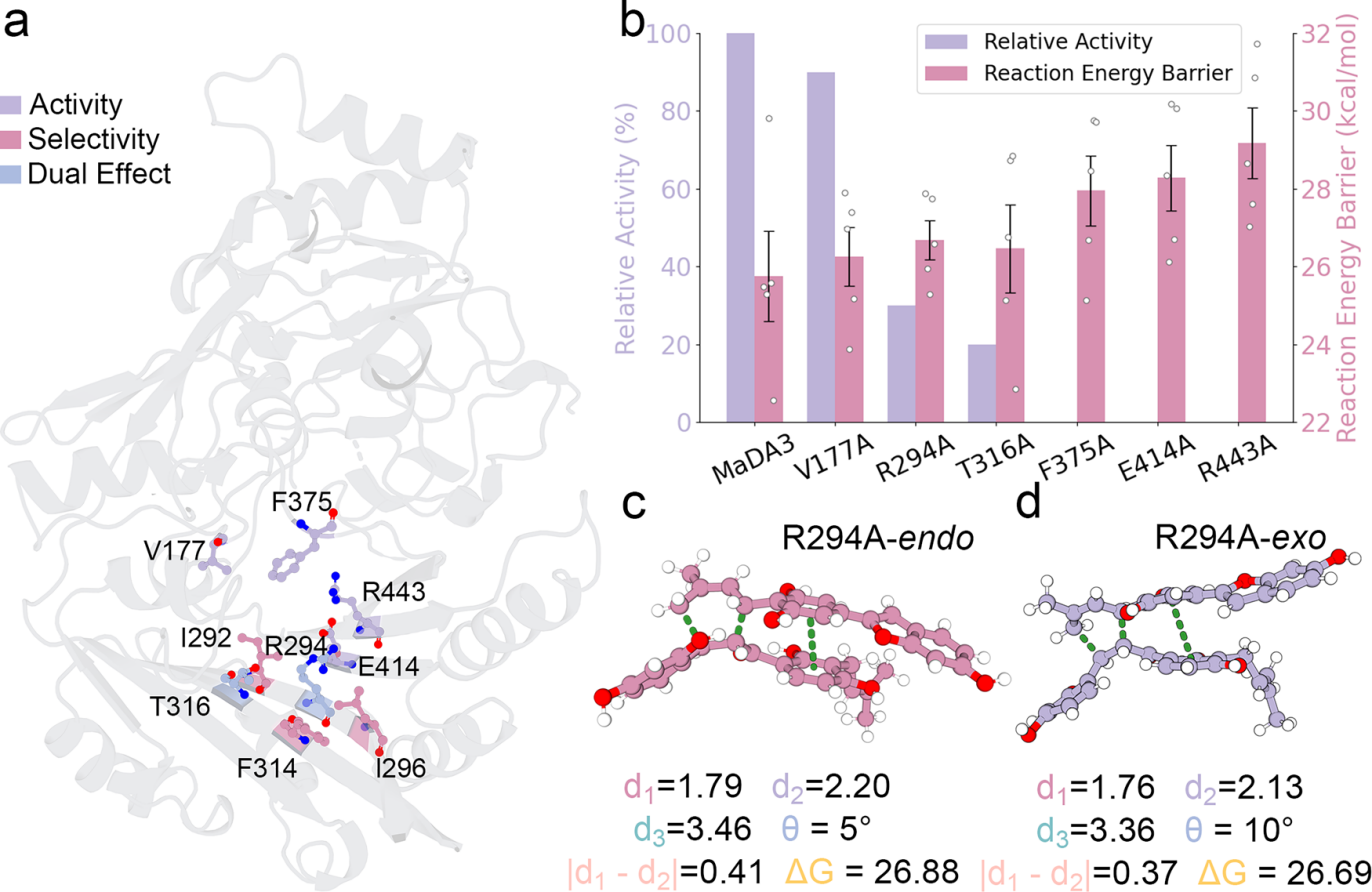
Mutation effects on activity and selectivity. **a** Key active-site residues color-coded by their functional role: activity (light purple), stereoselectivity (pink) and dual effect (blue). **b** Experimentally determined relative activities (purple bars, left axis) and computationally estimated activation free-energy barriers (*ΔG*^‡^, pink bars, right axis, kcal mol^−1^) for wild-type MaDA3 and alanine-scanning mutants; error bars represent the standard error of the mean from three independent biological replicates (*N* = 3). **c**, **d** Transition-state structures of the R294A mutant for the *endo* (**c**) and *exo* (**d**) pathways; green dashed lines indicate the forming C--C bonds. Key distances (*d*_1_, *d*_2_, *d*_3_) are in Å, *θ* is the dihedral angle in degrees, and *ΔG*^‡^ is in kcal mol^−1^. Bars indicate the mean; error bars represent one standard deviation (*N* = 5 independent simulation replicates.

The information obtained from ML/MM MetaD simulations on the wild type motivated us to examine whether this protocol could be applied to mutants, thereby probing how amino acid substitutions affect catalytic activity and stereoselectivity. Several residues located near the catalytic site were selected (Fig. 3a), as these first-shell residues are expected to strongly influence the TS structures.

For catalytic activity, we compared the predicted active free energies with experimentally measured relative activities of MaDA mutants (Fig. 3b). A clear relationship was observed that variants with higher *ΔG*^‡^ exhibited reduced activity, consistent with the principle that increased energetic barriers diminish catalytic turnover. In the experimental assay, reactions were quenched after a short incubation period^1^, such that variants with *ΔG*^‡^ exceeding a critical threshold yielded no detectable product. This trend was reproduced in our simulations, which predicted complete loss of activity for F375A, E414A, and R443A, all of which exhibited substantially higher barriers than the wild type or other mutants. Moreover, when considering the full panel of variants, the computed *ΔG*^‡^ values showed a strong linear correlation with experimentally measured relative activities, underscoring the ability of ML/MM MetaD to connect mutational effects at the atomic scale with macroscopic measures of enzymatic efficiency.

We further investigated the stereoselectivity by calculating the *ΔΔG* values. Consistent with previous experimental studies that identified stereodetermining residues^1^, MaDA-1 is intrinsically endo-selective, whereas MaDA-3 favors the exo product. To probe the molecular origins of this preference, we simulated a series of MaDA-3 mutants: R294G, R294A, MU3 (I292F, R294G, L296R), and MU5 (MU3 plus F314I and T316S), that mirror variants previously characterized experimentally. These substitutions progressively reduced the *exo* preference, with MU5 displaying an  ~ 1:2 *exo*:*endo* ratio and largely losing stereoselectivity. Although direct *ΔG*^‡^ measurements were not available, *ΔΔG* values could be inferred from product ratios via the Eyring equation^50^. Our simulations reproduced these values with a mean unsigned error of 0.61 kcal mol^−1^ in *ΔΔG* across the four MaDA-3 variants (Table S6). Three of the four mutants were predicted correctly in both sign and magnitude; the exception was Mu3, for which the simulation predicted an endo preference (*ΔΔG* = −1.19 kcal mol^−1^) whereas experiment indicates roughly equal product formation (*ΔΔG* ≈ 0). This deviation, while outside the sub-kcal mol^−1^ threshold, remains within the typical uncertainty of free-energy simulations.

Importantly, residue R294 is identified as a critical determinant that modulates both catalytic activity and stereoselectivity. Mutation of R294 to alanine reduced the activity to nearly 30% of the wild type (Fig. 3c) and shifted the product distribution from exclusively *exo* to an *endo*:*exo* ratio of 1:1.38 (Table S6). Consistent with the TS configurations observed in Fig. 2f, this result highlights the essential role of the *π*-cation interaction contributed by R294 in stabilizing the transition state. Notably, neighboring residues appear capable of reorganizing their side chains to conpensate sterically, leading to minimal differences in the geometric descriptors of *d*_3_ and *θ* between the two TS structures (Fig. 3c, d), which remain key parameters for enantiomer discrimination.

### Exploring the suitability of MaDA enzymes across a broad substrate spectrum

**Fig. 4:**
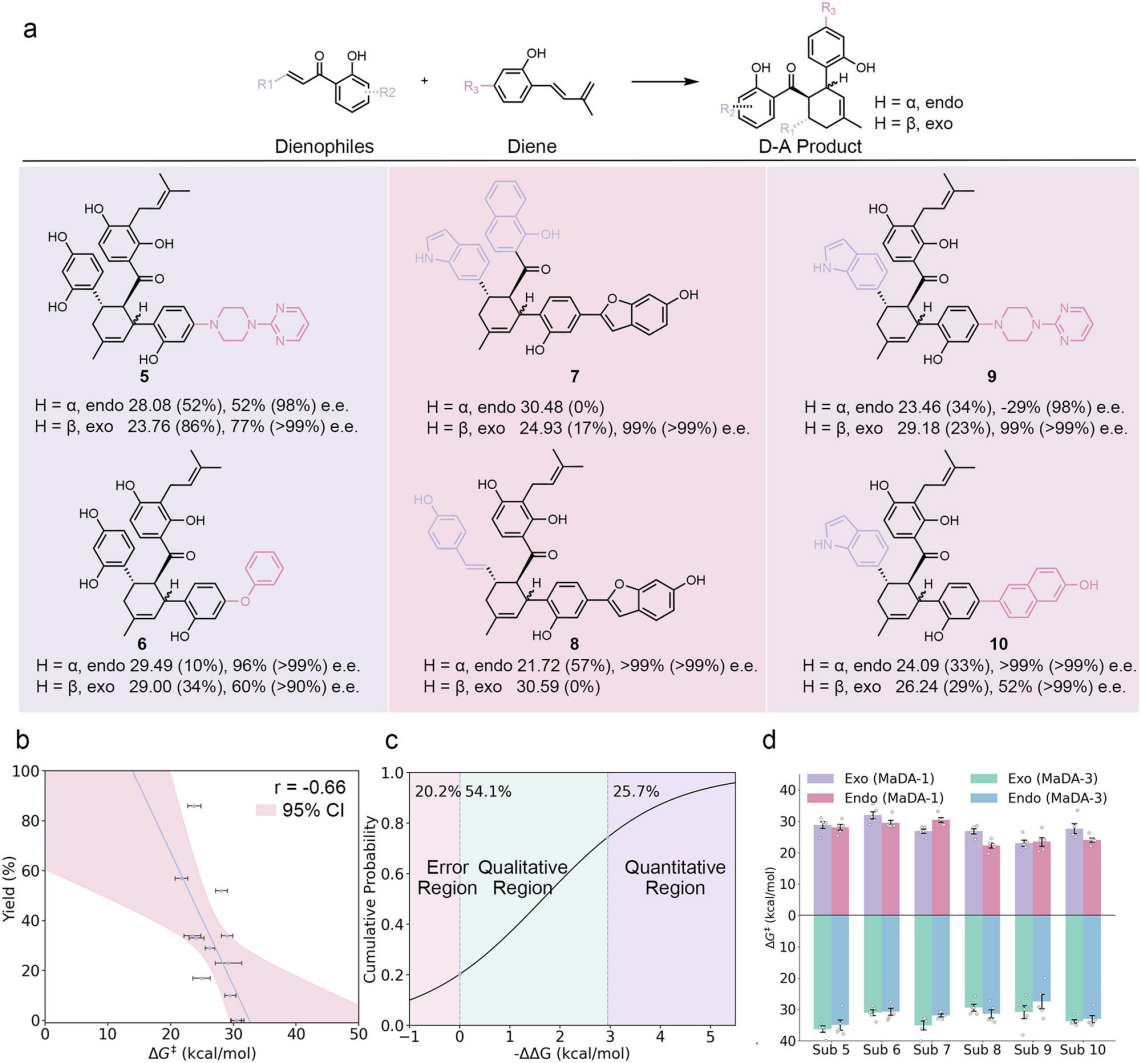
Generalizability of ML/MM across a broad catalytic substrate spectrum. **a** Estimated activation-energy barriers (in kcal mol^−1^) and corresponding enantiomeric excesses (e.e.) obtained from ML/MM simulations; experimental yields and e.e. values^1,2^ are shown in brackets. Substrate pairs are grouped by color: purple (**5**, **6**), green (**7**, **8**) and pink (**9**, **10**). **b** Linear regression of experimental yields versus estimated *ΔG*^‡^; the absolute Pearson correlation coefficient (*r*) and the 95% confidence interval (CI, shaded pink) are indicated. Error bars represent the standard error of the mean. **c** Empirical cumulative distribution function of −*ΔΔG*. The green, yellow and purple shaded regions denote the error region (−*ΔΔG* < 0, incorrect predominant enantiomer), qualitative region (correct enantiomer but >1% deviation in e.e.) and quantitative region (correct enantiomer with quantitative e.e. agreement), respectively. **d** Computed activation-energy barriers (in kcal mol^−1^) for the *exo* and *endo* pathways in MaDA-1 (purple and pink bars) and MaDA-3 (green and blue bars). Dots for **b** Bars for **d** indicate the mean; error bars represent one standard deviation (*N* = 5 independent simulation replicates).

Enzymes typically catalyze not only their natural substrates but also structurally related analogs. Understanding the substrate spectrum is therefore essential for assessing catalytic potential and guiding future industrial applications. We therefore performed virtual screening using our ML/MM MetaD protocol to estimate *ΔG*^‡^ and enantiomeric selectivities based on *ΔΔG* across a panel of substrate variants. Because the intramolecular D–A reaction involves two reacting fragments, both diene and dienophile functional groups were systematically modified as depicted in Fig. 4a, along with more extensive dual substitutions exemplified by substrates **9** and **10**.

As expected, *ΔG*^‡^ values showed a clear correlation with experimental yields and *ΔΔG* values accurately reflected enantiomeric excess (e.e.), as illustrated in Fig. 4a. This agreement across a diverse substrate panel underscores the strength of the ML/MM MetaD framework in capturing both catalytic activity and stereoselectivity. The only deviation was substrate **7**-*exo*, which displayed a low *ΔG*^‡^ yet poor yield. This discrepancy likely arises from altered binding or release modes introduced by multiple functional group substitutions, effects beyond what is represented by barrier heights alone. Importantly, such cases highlight the complementary roles of kinetics and binding dynamics in catalysis, rather than limitations of the ML/MM MetaD framework itself. Overall, the correlation between *ΔG*^‡^ and yield remained strong (*r* = −0.66, Fig. 4c), underscoring the robustness and predictive power of our approach.

Stereoselectivity was also well reproduced by our protocol, even though *ΔΔG* predictions are intrinsically sensitive to small free-energy differences, with an e.e. of 98% corresponding to only 2.95 kcal mol^−1^ difference. Across the tested substrate panel, approximately 25% of cases achieved full quantitative agreement with experiment, while an additional 54% were qualitatively correct, capturing the dominant enantiomer. This level of accuracy is notable given the sub-kcal mol^−1^ precision required to resolve stereochemical outcomes. The only clear deviation occurred for substrate **9**-*endo*, where the preferred enantiomer was reversed; however, the computed energy difference was only 0.78 kcal mol^−1^(Fig. 4d), a value well within the accepted uncertainty range of state-of-the-art free-energy simulations. Importantly, such small deviations, while magnified when expressed as enantiomeric excess, do not undermine the broader predictive capacity of the method. Instead, they highlight both the inherent difficulty of stereoselectivity prediction and the strength of the present approach, which achieves reliable performance across a chemically diverse substrate set.

## Discussion

Understanding enzymatic catalysis at the atomistic level is essential for enzyme engineering and industrial application, yet traditional computational approaches (MMFF, theozyme models, QM/MM) face intrinsic trade-offs between accuracy and efficiency. To overcome these limitations, we developed a unified multiscale framework that integrates universal reactive MLIPs into AMBER with a link-atom boundary scheme, enabling accurate and efficient description of chemical reactivity in enzymatic systems without the need for system-specific potential training. Coupled with PLUMED-driven metadynamics, the framework achieves nanosecond-timescale simulations that directly identify transition states in the MaDA family and in aqueous solution, and is immediately accessible to experimental researchers. The adoption of universal potentials and native enhanced sampling compatibility position this work as a foundation extensible beyond D–A chemistry to broader classes of enzymatic and condensed-phase reactions. It not only reproduces experimental and DFT trends in *ΔG*^‡^ and *ΔΔG*, but also reveals stereoselectivity mechanisms arising from aromatic stacking geometries and clarifies how key residues such as R294 exert dual control over activity and selectivity. Moreover, virtual screening across diverse substrates further demonstrated strong correlations between *ΔG*^‡^ and experimental yields, while *ΔΔG* predictions reached sub-kcal mol^−1^ accuracy in enantiomer discrimination. Together, these results underscore the generality of the framework for probing activity, selectivity, mutational effects, and substrate scope.

Although further advances in reactive MLIP development will be needed to extend ML/MM to radical, metalloenzyme, and photoenzyme catalysis, the present framework already bridges physics-based accuracy with machine-learning (ML) efficiency. We note that the current implementation adopts ME, which performs well for the D–A system studied here owing to its modest charge redistribution; extension to electrostatic embedding remains an important next step toward broader applicability across more polar or charge-transfer reactions. While ANI-1xnr serves as a capable and practical starting point for closed-shell pericyclic chemistry, more accurate and transferable next-generation reactive potentials, such as AIMNet2-rxn^33^, MACE-OMOL^51^, and UMA^52^, are expected to further improve accuracy and extend the scope of the framework to a wider range of reaction classes as they mature and become integrated. By unifying chemical reactivity, environment effects, and statistical sampling in a single platform, this work represents one contribution toward rational enzyme design and next-generation AI-based and physics-informed computational enzymology.

## Methods

### General settings

In this study, MD simulations were conducted using our ML/MM module implemented in the molecular simulation engine SANDER, based on the AMBER23 source code^41,42^. All calculations were performed on NVIDIA L40s GPUs and Intel Xeon Platinum 8462Y+ processors.

The protein was described with the AMBER14SB force field^53^, and the TIP3P model^54^ was used for water molecules. Notably, the MaDA family contains a non-natural residue, a histidine covalently bound to flavin adenine dinucleotide (FAD). This covalently linked fragment was extracted, and the broken valence formerly connected to the neighboring residue was capped with acetyl and methylamine groups. The geometry of the capped structure was optimized using Gaussian^55^ at the *ω*B97X/6-31G* level^56,57^, and RESP charges^58^ were derived at the HF/6-31G* level and ensure an integer total charge. The remaining parameters were assigned with GAFF2^59^. Ligands were parameterized with GAFF2, and partial atomic charges were obtained using the RESP method in Antechamber.

Systems were built with tleap (AMBERTOOLS23), solvated in a TIP3P water box with at least 10 Å padding between solute and box edges. Na^+^ and Cl^−^ ions were added to neutralize the system and adjust the salt concentration to 0.15 M.

All simulations began with energy minimization, consisting of 1000 cycles of steepest descent followed by 9000 cycles of conjugate gradient, for a total of 10,000 cycles. Bond lengths involving hydrogen atoms were constrained using the SHAKE algorithm^60^, and a nonbonded cutoff of 9.0 Å was applied. Minimization was considered converged when the root-mean-square gradient of the potential energy fell below 0.0001 kcal mol^−1^ Å^−1^.

Equilibration was carried out under cMD conditions without MLIPs, with an integration timestep of 1 fs. Simulations were run for 100,000,000 steps (100 ns). The temperature was maintained at 323 K, consistent with experimental conditions^1^, using a Langevin thermostat with a friction coefficient of 5.0 ps^−1^. Pressure was maintained at 1.01325 bar using the Berendsen barostat with a relaxation time of 2.0 ps. Periodic boundary conditions were applied, and a 9.0 Å cutoff was used for vdW and electrostatic interactions.

### Selection of the ML region

The ML region included residues 71, 176, 186, 261, 263, 292, 294, 296, 314, 316, 355, and 382, which have been experimentally demonstrated to play critical roles in catalytic activity and stereoselectivity^1^. The substrates were also included in the ML region, while the remaining parts of the system were treated with the MMFF. Residue M186 in MaDA3 was excluded because the ANI-1xnr potential currently does not support sulfur atoms.

### CV validation with steered MD

Prior to metadynamics, CVs were validated using the path CV^61^ formalism combined with steered molecular dynamics^62^ (SMD). Two forming C–C bond distances were employed as descriptors to define the reaction pathway. The path CV was expressed as a weighted combination of reference structures along the reaction coordinate:

$$s=\frac{{\sum }_{i=1}^{N}i\exp \left(-\lambda R[X-{X}_{i}]\right)}{{\sum }_{i=1}^{N}\exp \left(-\lambda R[X-{X}_{i}]\right)},\;\; z=-\frac{1}{\lambda }ln\left[{\sum}_{i=1}^{N}\exp \left(-\lambda R[X-{X}_{i}]\right)\right],$$

where *R*[*X* − *X*_*i*_] is the mean-square deviation between the instantaneous configuration *X* and the *i*^th^ reference structure *X*_*i*_, *s* measures the progression along the path, and *z* quantifies the distance from the path.

SMD was performed along the *s* coordinate with a moving restraint, in which the target value of *s* was gradually increased from 0.0 to 100.0 over the course of the simulation. A short equilibration stage of 100,000 steps was applied prior to pulling (STEP0), and an additional relaxation stage was included after reaching the final target (STEP1). A harmonic tube potential with a force constant of 200 kcal mol^−1^ Å^−2^ was applied on the *z* coordinate to confine sampling near the predefined path. Each simulation was carried out for a total of 2,000,000 steps with a timestep of 0.5 fs, corresponding to 1 ns of sampling, under the same thermodynamic conditions as cMD.

### Metadynamics

ML/MM MetaD simulations were initiated from equilibrated structures, under conditions nearly identical to those used in cMD, except that a smaller timestep of 0.5 fs was applied. Each simulation was run for 2,000,000 steps, corresponding to 1 ns of sampling. All simulations employed plumed-2.9^39,40^ compiled with AMBER.

Well-tempered metadynamics (WT-MetaD) was carried out by plumed using two CVs. For both CVs, an upper-wall restraint was imposed at 3.0 Å with a force constant of 50 kcal mol^−1^ Å^−2^ to prevent exploration of irrelevant regions of the free-energy landscape. Gaussian bias potentials were deposited every 50 MD steps (PACE = 50), with an initial height of 0.6 kcal mol^−1^ and a width of 0.08 Å for each CV. For each data point, ML/MM MetaD simulations were performed in five independent replicates, and the reported values represent the averages over these runs.

### Free-energy surface reconstruction and minimum-energy pathway identification

The free-energy surface (FES) was reconstructed using the sum_hills module of plumed, based on the HILLS files collected from MetaD simulations. Gaussian kernels were accumulated onto a 300 × 300 grid for *d*_1_ and *d*_2_ over the range of 1.4–3.5 Å, and the global minimum was set to zero as the reference energy.

From the resulting grid, the lowest free-energy pathway (LFEP) was identified using the LFEP algorithm^63^. The highest point along this pathway was taken as the TS.

### Committor analysis

According to the minimum-energy pathway, cpptraj was used to identify candidate structures corresponding to the most probable transition states by locating configurations with the root-mean-square deviation (RMSD) less than 0.1 Å in the *d*_1_ and *d*_2_ distances, defined as

$${{{\rm{RMSD}}}}=\sqrt{\frac{1}{N}{\sum }_{i=1}^{N}{({d}_{i}-{d}_{i}^{{{{\rm{ref}}}}})}^{2}}.$$

From each candidate structure, ML/MM MD simulations were restarted for 10,000 steps with a timestep of 0.5 fs. For each structure, ten independent simulations were performed, and the trajectories were analyzed collectively. A structure was considered a TS if the corresponding simulations demonstrated connectivity to both reactant and product states.

### Frequency analysis

Frequency calculations were performed on the TS structures by extracting the ML region, with link atoms placed according to Eq. (1). Energies, forces, and the Hessian matrix were evaluated using ANI-1xnr with PyTorch autograd^64^. The frequency analysis was conducted using the MAPLE package as follows^65^. The gradient and Hessian were obtained from the first- and second-order derivatives of the total energy,

$${g}_{i}=\frac{\partial E}{\partial {x}_{i}},\;\;{H}_{ij}=\frac{{\partial }^{2}E}{\partial {x}_{i}\partial {x}_{j}}.$$

To remove the dependence on nuclear masses, the Hessian was transformed into its mass-weighted form,

$${\widetilde{H}}_{ij}=\frac{{H}_{ij}}{\sqrt{{m}_{i}{m}_{j}}},$$

which was subsequently diagonalized to obtain eigenvalues *λ*_*k*_ and eigenvectors. The vibrational frequencies were then calculated directly from the eigenvalues as

$${\nu }_{k}=\frac{1}{2\pi c}\sqrt{{\lambda }_{k}},$$

where *c* is the speed of light.

### Reporting summary

Further information on research design is available in the Nature Portfolio Reporting Summary linked to this article.

## Supplementary information

Supplementary Information

Description of Additonal Supplementary Files

Supplementary Dataset 1

Supplementary Dataset 2

Supplementary Dataset 3

Supplementary Dataset 4

Supplementary Dataset 5

Reporting Summary

Transparent Peer Review file

## Source data

Source Data

## Data Availability

The data that support the findings of this study are available within this article and its Supplementary Information. The structural files used for modeling and the force field parameters for FAD are provided as Supplementary Data. Source data are provided with this paper.

## Code availability

The software developed in this work is available at https://github.com/ClickFF/MLMM4AMBER. The MAPLE package is available at https://github.com/ClickFF/MAPLE.
