# Supplementary material for: Multiscale machine learning molecular mechanics for mechanism and stereoselectivity of Diels-Alderase catalysis
Source: Nat Commun. 2026 May 13;17:6437. doi: 10.1038/s41467-026-72904-9 (PMC13377127; doi:10.1038/s41467-026-72904-9)
Supplement: Supplementary file 1 — Supplementary Information [file 41467_2026_72904_MOESM1_ESM.pdf]

Supplementary Information: Multiscale Machine Learning Molecular Mechanics for Mechanism and Stereoselectivity of Diels-Alderase Catalysis

Xujian Wang,[1,2,3] Haocheng Tang,[2] Xiongwu Wu,[4] Bernard R. Brooks,[4] Junmei Wang,[*,2] and Wan-Lu Li[*,1,5]

1 *Aiiso Yufeng Li Family Department of Chemical and Nano Engineering, University of California San Diego, CA 92093, United States*

2 *Department of Pharmaceutical Sciences and Computational Chemical Genomics Screening Center, School of Pharmacy, University of Pittsburgh, Pittsburgh, Pennsylvania 15261, United States*

3 *Department of Computational and Systems Biology, School of Medicine, University of Pittsburgh, Pittsburgh, Pennsylvania 15261, United States*

4 *Laboratory of Computation Biology, National Heart, Lung and Blood Institute, National Institutes of Health, Bethesda, MD, USA*

5 *Program of Materials Science and Engineering, University of California San Diego, CA 92093, United States*

E-mail: junmei.wang@pitt.edu; wal019@ucsd.edu

Theoretical Details

Universal Link Atom Treatment Across Heterogeneous MLIP Architectures

A central aspect of the present implementation is that a single link atom module interfaces with MLIPs built on fundamentally different representation paradigms. In practice, no modification to any MLIP architecture is required: the module simply presents each model with a capped molecular cluster containing a hydrogen link atom, and the architecture-specific behavior described below arises entirely from the native descriptor extraction scheme of each model. Unlike traditional QM/MM,[1] where the link atom merely adds basis functions to a Fock matrix, each MLIP architecture processes the link atom through a qualitatively distinct computational pathway determined by its descriptor extraction scheme. Below, we first describe the shared positioning and force redistribution formalism, then detail how the link atom enters the descriptor space of each of the five supported architectures, including ANI, MACE, AIMNet2, SpookyNet, and EANN, highlighting the unique challenges in each case.

Shared Formalism: Link Atom Positioning and Force Redistribution

The geometric positioning and chain-rule force redistribution are common to all architectures and inherited from the established QM/MM link atom formalism.[1] When a covalent bond between a QM-region atom Q and an MM-region atom M is severed, a link atom L is placed along the Q–M bond vector:

$$\mathbf{r}_L = \mathbf{r}_Q + \frac{d_L}{|\mathbf{r}_M - \mathbf{r}_Q|} (\mathbf{r}_M - \mathbf{r}_Q) \tag{1}$$

where $d_L = 1.09$ Åis the equilibrium C–H bond length. With $\hat{\mathbf{e}} = (\mathbf{r}_M - \mathbf{r}_Q)/R$ and $R = |\mathbf{r}_M - \mathbf{r}_Q|$, the force redistribution reads:

$$\mathbf{F}_L^{\perp} = \mathbf{F}_L - (\mathbf{F}_L \cdot \hat{\mathbf{e}})\,\hat{\mathbf{e}} \tag{2}$$

$$\mathbf{F}_M^{\text{mod}} = \frac{d_L}{R}\,\mathbf{F}_L^{\perp} \tag{3}$$

$$\mathbf{F}_Q \mathrel{+}= \mathbf{F}_L - \mathbf{F}_M^{\text{mod}}, \quad \mathbf{F}_M \mathrel{+}= \mathbf{F}_M^{\text{mod}} \tag{4}$$

Our contribution lies in how the force $\mathbf{F}_L$ is computed by the MLIP, a process that differs fundamentally across architectures, as detailed below.

ANI: Link Atom in Symmetry Function Descriptors

The ANI family[2–4] decomposes the total energy into atomic contributions via species-specific neural networks: $E = \sum_i \mathrm{NN}^{Z_i}(\mathbf{G}_i^{Z_i})$, where $\mathbf{G}_i^{Z_i}$ is the Atomic Environment Vector (AEV) consisting of species-resolved radial and angular sub-vectors.[2] The link atom L (species H) modifies the AEVs of every ML-region atom i within the cutoff:

Radial modification. For any QM atom i with $R_{iL} < R_c^{\text{rad}}$, L contributes to the hydrogen radial sub-AEV:

$$G_{i,m}^{R,\text{H}} \mathrel{+}= \exp\!\big[-\eta_R (R_{iL} - R_s^{(m)})^2\big]\; f_c(R_{iL}) \tag{5}$$

where $f_c(R) = \frac{1}{2}\cos(\pi R/R_c) + \frac{1}{2}$ for $R < R_c$ (zero otherwise), and m indexes the radial shift parameters $R_s^{(m)}$.

Angular modification. For any QM atom i and neighbor j both within R_c^{ang} of i, the triplet (j, i, L) contributes to the angular sub-AEV $\mathbf{G}_i^{A,(Z_j,\text{H})}$:

$$G_{i,m}^{A,(Z_j,\text{H})} \mathrel{+}= 2^{1-\zeta}\big(1 + \cos(\theta_{jiL} - \theta_s)\big)^{\zeta}\; \exp\left[-\eta_A \left(\frac{R_{ij} + R_{iL}}{2} - R_s\right)^2\right] f_c(R_{ij})\, f_c(R_{iL}) \tag{6}$$

where θ_{jiL} is the angle at center atom i between neighbors j and L.

Link atom's own AEV. The link atom has its own AEV $\mathbf{G}_L^{\mathrm{H}}$ constructed from all QM neighbors within its cutoff. The atomic energy $E_L = \mathrm{NN}^{\mathrm{H}}(\mathbf{G}_L^{\mathrm{H}})$ contributes to the total ML energy, and the force $\mathbf{F}_L = -\nabla_{\mathbf{r}_L} E$ is redistributed via Eqs. 2–4.

MACE: Link Atom as a Graph Node in Equivariant Convolutions

MACE[5,6] builds on the Atomic Cluster Expansion (ACE) framework, constructing body-ordered equivariant features through iterated message passing on a molecular graph $\mathcal{G} = (\mathcal{V}, \mathcal{E})$.[5] Unlike ANI, the link atom is not merely a radial perturbation, it is a first-class node in the molecular graph with profound consequences:

(a) Edge construction and species encoding. The link atom L participates in edge enumeration:

$$\mathcal{E} = \big\{(i,j) \;\big|\; |\mathbf{r}_i - \mathbf{r}_j| < r_{\text{cut}},\;\; i,j \in \{1,\ldots,N_Q + N_L\}\big\} \tag{7}$$

generating edges to/from all QM atoms within r_{cut}. L receives the hydrogen embedding: $h^{(0)}_{L,k00} = W_{k,Z=1}$, providing species-specific learned features that influence all downstream computations.

(b) Contribution to the one-particle basis. In the first message-passing layer, L contributes to the atomic basis of every atom i with $r_{iL} < r_{\text{cut}}$:

$$A^{(1)}_{i,\,klm} \mathrel{+}= R^{(1)}_{kl}(r_{iL})\; Y_l^m(\hat{\mathbf{r}}_{iL})\; W^{(1)}_{k,Z_{\mathrm{H}}} \tag{8}$$

where $R^{(1)}_{kl}$ are learnable radial functions expanded in Bessel basis, Y_l^m are real spherical harmonics, and $W^{(1)}_{k,Z_{\mathrm{H}}}$ are species-dependent learnable weights. This contribution propagates into all body-ordered features via the symmetric contraction. For correlation order $\nu = 3$ (four-body terms), the link atom enters cross-terms of the form:

$$B_i^{(\nu)} \ni \mathcal{C} \cdot A_i^{[\text{from } L]} \cdot A_i^{[\text{from } j]} \cdot A_i^{[\text{from } k]} \tag{9}$$

encoding angular correlations between the link atom and pairs of QM atoms through Clebsch–Gordan (CG) coupled spherical harmonics, a many-body equivariant interaction.

(c) Multi-hop message propagation. Through T layers of message passing, the link atom's influence propagates to atoms up to $T \times r_{\text{cut}}$ away. At each layer, the link atom both sends equivariant messages (shaped by its species weight and local geometry) and receives messages from QM neighbors, enabling iterative refinement of its feature representation.

(d) Effective body order. With $\nu = 3$ and $T = 2$, the effective body order is $(\nu^{T+1} - 1)/(\nu - 1) = (3^3 - 1)/(3 - 1) = 13$. The link atom participates in up to 13-body correlations through the iterated symmetric contraction, a far richer interaction than the 3-body terms captured by ANI's angular symmetry functions.

AIMNet2: Link Atom in Message Passing with Charge Equilibration

AIMNet2[7,8] is an invariant message-passing neural network that decomposes the total energy into local, dispersion, and Coulomb contributions.[7] The link atom participates in both geometric and electronic channels:

(a) Scalar/vector convolution. The link atom contributes to every QM atom's scalar embedding through the hydrogen 2D embedding:

$$v_{isd} \mathrel{+}= \mathbf{a}_{ds}(Z_{\text{H}}) \odot g_{i,L,s} \tag{10}$$

where $\mathbf{a}_{ds}(Z_{\text{H}}) \in \mathbb{R}^{d \times s}$ is the learnable 2D embedding matrix for hydrogen, $g_{i,L,s} = \exp[-\eta_s(r_{iL} - r_s)^2]\, f_c(r_{iL})$ is the Gaussian radial basis, and $\odot$ denotes the Hadamard product. It similarly contributes to the vector embedding, encoding the angular direction $\hat{\mathbf{r}}_{iL}$ from atom i to the link atom.

(b) Iterative feature refinement. Through T iterations of message passing, the link atom both sends and receives feature updates, allowing its representation to adapt to the local QM-region environment.

(c) Critical: Charge equilibration with link atoms. This is the most consequential aspect of the link atom in AIMNet2. When a link atom is added, it modifies the effective electron count of the ML subsystem. The Neural Charge Equilibration (NQE) constraint[8] enforces $\sum_i \tilde{q}_i = Q$, where the sum now includes the link atom:

$$\tilde{q}_i = q_i + f_i \cdot \left(Q - \sum_{j=1}^{N} q_j \right) \tag{11}$$

where q_i is the raw predicted charge and f_i is a neural-network-predicted weight analogous to the Fukui function ($\sum_i f_i = 1$). The link atom acquires a non-trivial partial charge $\tilde{q}_L$ through this redistribution mechanism. For AIMNet2-NSE (Neural Spin Equilibration),[8] both the total charge Q and spin multiplicity S must correctly reflect the ML subsystem including the link atom:

$$\tilde{q}_i^\alpha = q_i^\alpha + f_i^\alpha \cdot \left(Q^\alpha - \textstyle\sum_j q_j^\alpha\right), \quad Q^\alpha = \tfrac{1}{2}(Q - S + 1) \tag{12}$$

SpookyNet: Link Atom in Nuclear/Electronic Embedding with Nonlocal Interactions

SpookyNet[9] is the only architecture in our framework that employs both distance-dependent local interactions and distance-independent nonlocal interactions (self-attention), combined with explicit electronic state encoding.[9] The link atom has the richest interaction pathway of all five architectures:

(a) Nuclear charge embedding. The link atom enters as hydrogen ($Z = 1$):

$$\mathbf{e}_{Z=1} = \mathbf{M} \cdot \mathbf{d}_\mathrm{H} + \tilde{\mathbf{e}}_\mathrm{H} \tag{13}$$

where $\mathbf{d}_\mathrm{H} = (1, 0, \ldots, 0, 1, 0, 0, 0, 1)^T$ encodes the $1s^1$ electron configuration, $\mathbf{M}$ is a learnable projection matrix, and $\tilde{\mathbf{e}}_\mathrm{H}$ is a freely learnable bias.

(b) Electronic state embedding. Through a softplus-weighted attention mechanism, the link atom receives a share of the total charge/spin:

$$a_L = \frac{\Psi \cdot \ln\bigl(1 + \exp(\mathbf{q}_L^T \mathbf{k}/\sqrt{F})\bigr)}{\sum_j \ln\bigl(1 + \exp(\mathbf{q}_j^T \mathbf{k}/\sqrt{F})\bigr)} \tag{14}$$

where $\Psi = Q$ (charge) or S (spin). The attention weights satisfy $\sum_i a_i = \Psi$, exactly distributing the global electronic information across atoms. Since $\mathbf{q}_L = \mathrm{linear}(\mathbf{e}_{Z=1})$, the fraction of electronic embedding allocated to the link atom depends on the learned interaction between the hydrogen nuclear embedding and the charge/spin key vectors.

(c) Local interaction: s/p/d channel contributions. The link atom contributes to the scalar, vector, and tensor channels of every QM atom within r_{cut}:

$$s_i \mathrel{+}= \mathrm{resmlp}_s(\tilde{\mathbf{x}}_L) \odot (G_s \cdot \rho_s(r_{iL})) \tag{15}$$

The p- and d-channel contributions encode angular information through spherical harmonics $Y_1^m(\hat{\mathbf{r}}_{iL})$ and $Y_2^m(\hat{\mathbf{r}}_{iL})$, providing directional sensitivity to the link atom position.

(d) Nonlocal interaction: global attention. Uniquely among all architectures, SpookyNet's nonlocal module allows the link atom to interact with every atom in the ML region regardless of distance. The link atom's query/key/value vectors participate in global self-attention, meaning its influence is not bounded by r_{cut}.

EANN: Link Atom in GTO-Based Embedded Atom Density

EANN[10–12] represents the atomic environment through a squared density built from Gaussian-type orbital (GTO) primitives.[10] The link atom contributes to the embedded atom density (EAD) of every QM atom i within r_c:

(a) Contribution to the EAD. The link atom L (with orbital coefficient $c_L = c_{\mathrm{H}}$) adds to the EAD:

$$\rho_{i,n} \ni \ldots + \frac{l!}{l_x!\, l_y!\, l_z!} \left[\ldots + c_{\mathrm{H}} \sum_m d_{mn}\, x_{iL}^{l_x} y_{iL}^{l_y} z_{iL}^{l_z}\, e^{-\alpha_m (r_{iL} - r_m)^2} f_c(r_{iL}) + \ldots \right]^2 \tag{16}$$

where $l_x + l_y + l_z = l$ is the total angular momentum ($l = 0$: s-type, $l = 1$: p-type, $l = 2$: d-type), and α_m, r_m are the Gaussian width and center for the m-th primitive.

(b) Implicit three-body correlations with the link atom. The squared-density construction generates cross-terms between the link atom and real QM neighbors:

$$\rho_{i,n} \ni \ldots + 2\, c_{\mathrm{H}}\, c_j\, r_{iL}^l\, r_{ij}^l\, \cos^l(\theta_{LiJ}) \sum_{m,m'} d_{mn}\, d_{m'n}\, g_m(r_{iL})\, g_{m'}(r_{ij}) + \ldots \tag{17}$$

This encodes the angle θ_{LiJ} between the link atom direction and each QM neighbor direction, with increasing angular resolution for higher l. The angular dependence $\cos^l(\theta)$ emerges naturally from the Cartesian GTO formulation without explicit angular symmetry functions.

Link atom's own density. L has its own EAD ρ_L constructed from all QM neighbors within r_c. Its atomic energy $E_L = \mathrm{NN}^{\mathrm{H}}(\rho_L)$ contributes to the total.

Comparative Analysis and Universal Framework

Table S1 summarizes the qualitative differences in how the link atom participates across the five architectures. The diversity of descriptor extraction mechanisms underscores the non-trivial nature of providing a universal link atom interface. Specifically, our framework must handle:

1. **Heterogeneous input tensor formats.** ANI expects species-indexed coordinate arrays; MACE requires edge index tensors and one-hot species vectors; AIMNet2 uses 2D embedding matrices; SpookyNet needs nuclear charge arrays plus global Q and S; EANN requires neighbor lists with Cartesian displacement vectors.

2. **Architecture-specific validation.** The link atom species ($Z = 1$, hydrogen) must be in the model's training domain. For MACE-OFF23, this means $Z_L \in \{$H, C, N, O, F, P, S, Cl, Br, I$\}$; for ANI-1xnr, $Z_L \in \{$C, H, N, O$\}$. Our framework validates this at initialization.

3. **Force computation consistency.** All backends compute forces via PyTorch autograd ($\mathbf{F} = -\nabla E$ via `energy.backward()` or `torch.autograd.grad`), but differ in whether gradients are retained for higher-order derivatives, whether the computation graph is preserved, and how the gradient tensors are shaped and indexed. The link atom force redistribution (Eqs. 2–4) must receive consistent force arrays regardless of upstream computation details.

4. **Unit conversion.** ANI and AIMNet2 output energies in Hartree ($\times 627.509 \rightarrow$ kcal/mol), while MACE, SpookyNet, and EANN output in eV ($\times 23.0605 \rightarrow$ kcal/mol). Forces undergo corresponding conversions.

5. **Global electronic state management.** For AIMNet2 (NQE/NSE) and SpookyNet (electronic embedding), the total charge Q and spin multiplicity S of the ML subsystem must be correctly propagated through the Fortran–C++ interface, accounting for the link atom's effect on effective electron count.

Table S1: Architecture-specific link atom participation. Each row represents a distinct computational pathway through which the link atom influences the MLIP's energy and force predictions.

**Feature**	**ANI**	**MACE**	**AIMNet2**	**SpookyNet**	**EANN**
**Descriptor type**	Symmetry functions (AEV)	ACE + message passing	2D embedding + message passing	s/p/d channels + attention	GTO density
**Link atom role**	Additive contribution to radial & angular sub-AEVs	Graph node in $E(3)$-equivariant convolutions	Neighbor in scalar/vector conv. + charge equilibration	Four-fold: nuclear, electronic, local, nonlocal	Orbital density source for GTO-based EAD
**Angular encoding**	Explicit θ_{ijk}	Spherical harmonics Y_l^m + CG coupling	Vector channel with $\hat{\mathbf{r}}_{ij}$	p/d channels with Y_1^m, Y_2^m	Implicit via $\cos^l(\theta)$ from squared GTO
**Body order**	3-body (pairwise + angular)	Up to $\frac{\nu^{T+1}-1}{\nu-1}$-body via iterated contraction	3-body (with iterative refinement)	Many-body via nonlocal attention	3-body via squared density
**Range of influence**	$R_c^{\text{ang}} \leq 3.5$ Å	$T \times r_{\text{cut}}$ (multi-hop)	$R_c = 5.0$ Å (+ Coulomb at 10 Å)	**Unbounded** (nonlocal attention)	$r_c \leq 6.0$ Å
**Species encoding**	Species-resolved sub-AEVs	One-hot $\rightarrow$ learnable embedding	2D embedding matrix per element	Electron configuration + bias	Orbital coefficient c_Z
**Electronic state**	None	None	NQE/NSE: Q, S	Charge + spin attention	None
**Energy unit**	Hartree	eV	Hartree	eV	eV

Computational Methods

Reactive Machine Learning Interatomic Potentials Used in This Work

ANI-1xnr

ANI-1xnr[4] is a general-purpose reactive MLIP developed by Zhang et al. for condensed-phase chemistry of the elements C, H, N, and O. The model builds on the ANI architecture,[2,3,13] which decomposes the total energy into atomic contributions via species-specific neural networks operating on Atomic Environment Vectors (AEVs), a modified Behler–Parrinello symmetry function representation.

Architecture. ANI-1xnr uses radial ($R_c^{\mathrm{rad}} = 5.2$ Å) and angular ($R_c^{\mathrm{ang}} = 3.5$ Å) cutoffs to construct the AEV descriptor for each atom. The AEV consists of species-resolved radial and angular sub-vectors, and a separate feed-forward neural network is trained for each element (C, H, N, O). The total energy is:

$$E = \sum_i \mathrm{NN}^{Z_i}\big(\mathbf{G}_i^{Z_i}\big) \tag{18}$$

where $\mathbf{G}_i^{Z_i}$ is the AEV of atom i with element Z_i. Similar to previous ANI models, ANI-1xnr predicts energies based solely upon atomic positions and element types; it does not explicitly depend on the total charge or spin multiplicity. Forces are obtained via automatic differentiation. In our ML/MM framework, the model is loaded through a C++ interface linked via LibTorch (the C++ frontend of PyTorch[14]).

Training data. Unlike prior ANI models that were trained on near-equilibrium organic molecules in vacuo, ANI-1xnr was developed through an automated nanoreactor active learning (NR–AL) workflow designed to sample condensed-phase reactive chemistry.[4] The NR–AL procedure initializes periodic simulation cells with randomly placed small molecules (e.g. C_2H_2, NH_3, H_2O, CO, CH_4) and drives MLIP-accelerated molecular dynamics under oscillating temperature (up to ∼6000 K) and density conditions to promote chemical reactions. High-uncertainty structures, identified by ensemble disagreement, are selected and labelled with single-point DFT calculations performed using CP2K[15] at the BLYP[16,17]/TZV2P[18] level

of theory with Goedecker–Teter–Hutter (GTH) pseudopotentials[19] and Grimme D3 dispersion correction with zero damping.[20] All reference calculations used unrestricted Kohn–Sham DFT with singlet spin multiplicity for the periodic simulation cell. After more than 50 AL iterations, the resulting training set comprises 26,650 periodic simulation cells with an average system size of 139 atoms, spanning densities from $\sim$0.03 to $>$2.0 g cm^{-3} and containing over 1,200 unique molecular species identified by cross-referencing with the PubChem database.[4]

AIMNet2-NSE

AIMNet2-NSE[7,8] is an invariant message-passing neural network potential with Neural Charge/Spin Equilibration, developed for open-shell and charged reactive chemistry. It extends the AIMNet2 architecture[7] with explicit handling of both total charge Q and spin multiplicity S.

Architecture. AIMNet2-NSE uses a 2D learnable atomic embedding and T iterations of message-passing convolution with scalar ($l = 0$) and vector ($l = 1$) channels, using Gaussian radial basis functions ($S = 16$ radial shells) with a cutoff of $R_c = 5.0$ Å.[7] The total energy is decomposed into local, dispersion (D4), and Coulomb contributions:

$$U_{\text{Total}} = U_{\text{Local}} + U_{\text{Disp}} + U_{\text{Coul}} \tag{19}$$

The critical distinguishing feature is the Neural Spin Equilibration (NSE) module,[8] which constrains the spin-resolved partial charges through:

$$\tilde{q}_i^\alpha = q_i^\alpha + f_i^\alpha \cdot \left(Q^\alpha - \sum_j q_j^\alpha\right), \quad Q^\alpha = \tfrac{1}{2}(Q - S + 1) \tag{20}$$

where f_i^α is a learned Fukui-like weight ensuring $\sum_i \tilde{q}_i^\alpha = Q^\alpha$. This enables the model to distinguish between different electronic states of the same molecular geometry, which is essential for modelling radical intermediates, homolytic bond cleavage, and open-shell transition states.

Training data. AIMNet2 was trained on a large, chemically diverse dataset of organic and elemental-organic molecules spanning 14 elements (H, B, C, N, O, F, Si, P, S, Cl, As, Se, Br, I), with reference calculations performed at the ωB97X-D[21]/def2-TZVPP level of theory.[7] The training data include neutral and charged species across multiple charge states, with conformations sampled by normal-mode displacement, torsion scanning, and molecular dynamics.

Gas-Phase Potential Energy Surface Validation of ANI-1xnr

To assess the fidelity of the ANI-1xnr potential across the entire reactive landscape, we constructed two-dimensional potential energy surfaces (PES) for both the *endo* and *exo* Diels–Alder pathways and compared the ANI-1xnr predictions against DFT reference calculations.

PES Construction

For each stereochemical pathway (*endo* and *exo*), the two forming C–C bond distances (denoted AB and CD) were scanned from 1.5 to 4.0 Åin increments of 0.05 Å, yielding a uniform grid of $51 \times 51 = 2{,}601$ structures per pathway (5,202 structures in total). At each grid point, a constrained geometry optimisation was performed using Gaussian 16[22] at the ωB97X/6-31G*[23,24] level of theory: the two scanned bond distances were frozen while all remaining internal coordinates were fully relaxed. The resulting DFT energies define the reference PES.

For each of the 5,202 optimised structures, single-point energies were then evaluated with ANI-1xnr using MAPLE[25] package. MAPLE provides a unified inference interface for multiple MLIP backends including ANI-family models, enabling batch evaluation of energies and forces on arbitrary molecular geometries.

Minimum-Energy Path Search

To further assess whether the MLIP preserves the intrinsic reaction coordinate, we performed minimum-energy path (MEP) searches on both the DFT and ANI-1xnr surfaces using a modified Dijkstra algorithm with a path-length penalty.

Algorithm. The two-dimensional PES is represented as a regular grid graph in which each node (i, j) corresponds to a grid point with energy E_{ij}, and edges connect each node to its eight nearest neighbours (including diagonals). For a diagonal step the edge length is $\sqrt{2}\,\Delta$, whereas for an axial step it is Δ, where $\Delta = 0.05$ Åis the grid spacing.

To find the path of lowest energy barrier that also avoids unnecessarily circuitous routes, we define a composite cost function. For a path $\mathcal{P} = \{(i_0, j_0), (i_1, j_1), \ldots, (i_N, j_N)\}$ from the

reactant node to the product node, the cost is:

$$C(\mathcal{P}) \;=\; \max_{k\in\mathcal{P}} E_{i_k j_k} \;+\; \lambda \sum_{k=1}^{N} d_k \tag{21}$$

where $\max_k E_{i_k j_k}$ is the highest energy encountered along the path (the bottleneck energy), d_k is the Euclidean step length between consecutive nodes, and λ is a path-length penalty coefficient (in kcal mol^{-1} Å^{-1}) that penalises unnecessarily long detours. This formulation seeks the path whose maximum energy is lowest, with the penalty term breaking ties in favour of shorter, more direct routes. A value of $\lambda = 1.0$ was used throughout.

This cost function is minimised globally using Dijkstra's algorithm adapted for the composite objective: at each step, the algorithm expands the unvisited node with the smallest accumulated cost

$$C_{\text{acc}}(i_k, j_k) \;=\; \max\!\big(C_{\text{acc}}(i_{k-1}, j_{k-1}),\; E_{i_k j_k}\big) \;+\; \lambda\, d_k \tag{22}$$

initialised with $C_{\text{acc}}(i_0, j_0) = E_{i_0 j_0}$. Because the cost is monotonically non-decreasing along any extension of the path, the algorithm is guaranteed to find the global optimum.

Post-processing. The raw grid path was smoothed using a cubic B-spline interpolation with 500 intermediate points, followed by uniform arc-length resampling to N_{pts} evenly spaced points along the path. Energies at the resampled coordinates were obtained by bilinear interpolation on the original grid.

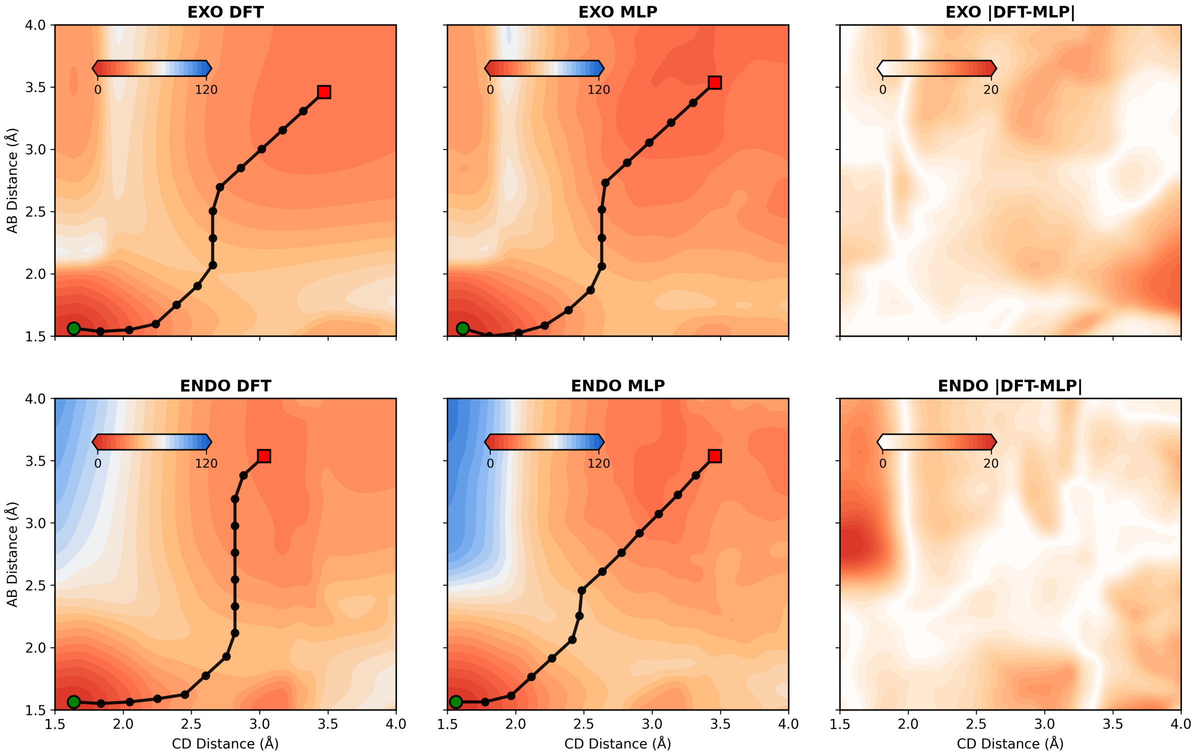

Figure S1: Comparison of two-dimensional potential energy surfaces for the *exo* (top row) and *endo* (bottom row) Diels–Alder pathways. Left: DFT reference (ωB97X/6-31G*). Centre: ANI-1xnr single-point energies. Right: unsigned error $|E_{\mathrm{DFT}} - E_{\mathrm{MLP}}|$. The energy colour scale is in $\mathrm{kcal\,mol^{-1}}$. Black curves with dots denote the minimum-energy paths obtained by the Dijkstra algorithm. Green circles mark the reactant minimum; red squares mark the product minimum. RMSEs over the full grid are 4.39 $\mathrm{kcal\,mol^{-1}}$ (*exo*) and 4.44 $\mathrm{kcal\,mol^{-1}}$ (*endo*).

Link Atom Energy Conservation Validation

To assess the numerical error potentially introduced by the link atom scheme at the ML/MM boundary, we performed a series of NVE molecular dynamics benchmarks on a tyrosine–tyrosine (Tyr–Tyr) dipeptide in vacuum (45 atoms). The system was constructed using `tLEaP` with the ff14SB force field[26] and energy-minimized (5000 cycles) prior to dynamics. An initial NVE equilibration was carried out at $300\,\mathrm{K}$ with $\Delta t = 1.0\,\mathrm{fs}$ for $1\,\mathrm{ns}$ using the classical force field. Four production NVE simulations were then restarted from the equilibrated coordinates and velocities (Figure S2). All runs used `ntt` = 0 (microcanonical ensemble), no periodic boundaries, and an effectively infinite nonbonded cutoff (9999 Å). The ANI1-xnr potential was employed.[4] In the ML/MM simulation, only the sidechain atoms from C_β onward (atoms @7–21 and @28–42, 30 atoms total) were assigned to the ML region, with link atoms placed at each C_α–C_β boundary.

The energy conservation of the ML/MM simulation ($\Delta E = 0.009$ kcal/mol over $0.5\,\mathrm{ns}$, Figure S2) confirms that the link atom treatment at the ML/MM boundary introduces negligible numerical error into the Hamiltonian, comparable to the intrinsic integration noise of the ML potential itself.

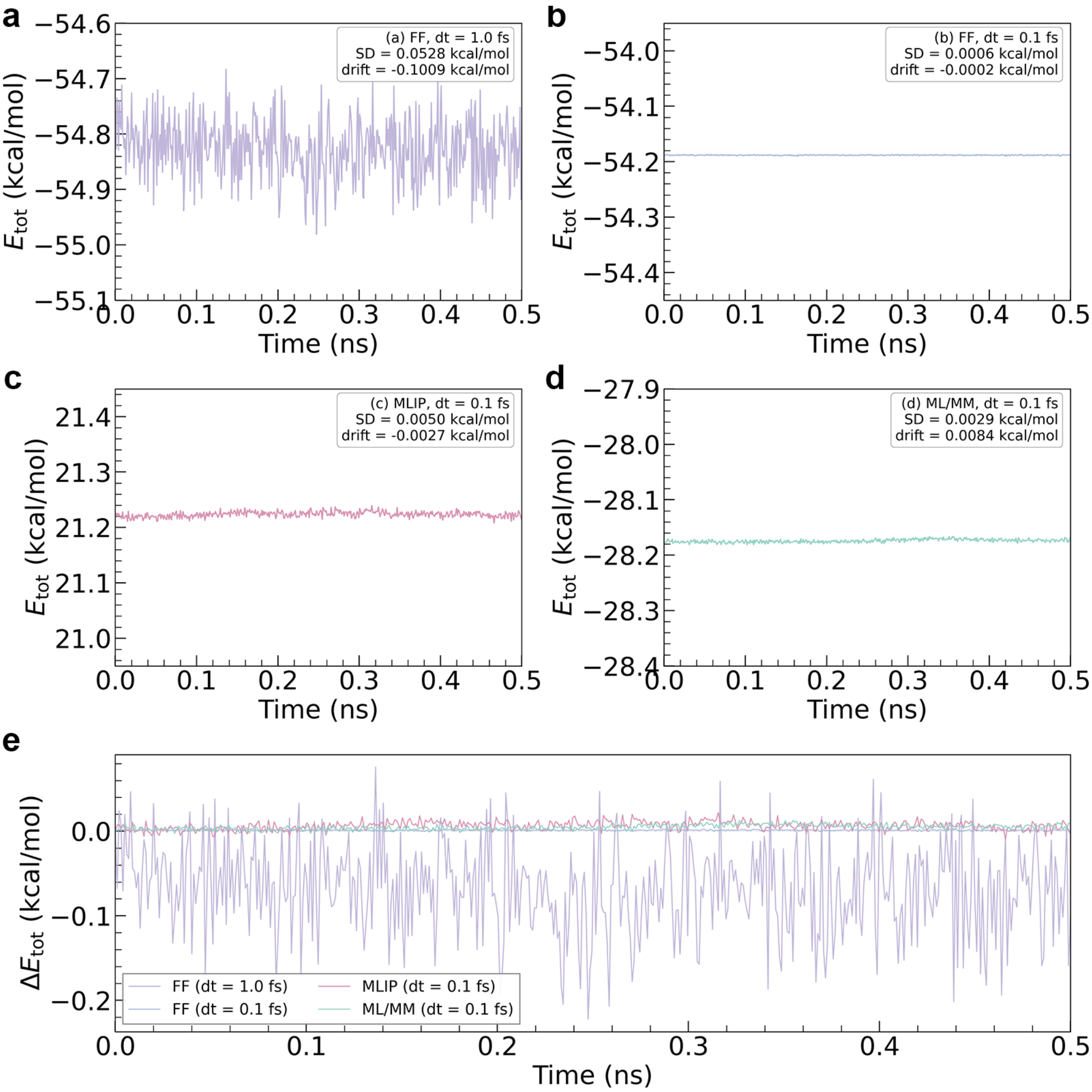

Figure S2: Energy conservation benchmarks for NVE simulations of the Tyr–Tyr dipeptide in vacuum. (a)–(d) Total energy $E_{\mathrm{tot}}(t)$ for each simulation over $0.5\,\mathrm{ns}$, with the method, timestep, standard deviation (SD), and total energy drift annotated in each panel. All panels share a $0.5\,\mathrm{kcal/mol}$ y-axis range to facilitate visual comparison. (e) Overlay of the total energy drift $\Delta E_{\mathrm{tot}}(t) = E_{\mathrm{tot}}(t) - E_{\mathrm{tot}}(0)$ for all four simulations.

QM/MM and ML/MM Performance Benchmarks

To assess the computational efficiency of our ML/MM interface, we performed a systematic benchmarking study by comparing ML/MM and QM/MM simulation speeds across a series of test systems with progressively expanding QM/ML regions. All the simulations are performed on Intel(R) Xeon(R) w7-3455 with NVIDIA RTX 5070Ti.

QM/MM setup. QM/MM simulations were carried out using the GROMACS–CP2K interface obtained through joint compilation (GROMACS 2024.2 and CP2K 2024.2),[15,27] which automatically generates a CP2K input template from the system topology. The auto-generated template was modified only to replace the exchange–correlation functional and basis set with ωB97X/6-31G*; all other settings were retained. A time step of 0.5 fs was used and each simulation was run for 100 steps, with the per-step wall-clock time averaged over all steps to estimate throughput (ps hour^{-1}). The spin multiplicity was set to 1 throughout; the total QM charge was adjusted for each system according to the net charge of the included residues (see Table S2).

ML/MM setup. ML/MM simulations employed the interface developed in this work, using either ANI-1xnr or AIMNet2-NSE as the ML potential. The same 0.5 fs time step was applied. Each simulation was run for 10,000 steps and the average throughput was computed from the final 1,000 steps to ensure thermal equilibration of the timing measurements.

Test systems. Fourteen test systems were constructed to span a wide range of ML/QM region sizes (Table S2). Cases 1–11 follow an incremental expansion protocol in which one additional protein residue sidechain is added to the ML region at each step, beginning from the substrate alone (Case 1, 84 atoms, no link atoms) and reaching the complete first-coordination-shell set of 11 fragments (Case 11, 222 atoms including 10 link atoms). Case 12 was designed to isolate the cost of the link-atom boundary treatment: it contains the same 78 sidechain atoms and 6 link atoms as would result from selecting those six sidechains without the substrate, yielding a region of identical total size (84 atoms) to Case 1 but with six QM/MM boundary bonds instead of none. Cases 13 and 14 correspond to the full first-shell (4 Å) and second-shell (8 Å)

residue selections, comprising 19 and 58 residues respectively. Because QM/MM calculations become prohibitively expensive at these scales, QM/MM reference timings were not collected for Cases 13 and 14; ML/MM throughput values are reported for completeness.

Table S2: Composition of QM/ML Benchmark Systems

Case	QM/ML Region	ML atoms[a]	Link atoms	QM charge	Notes
1	Substrate	84	0	0	Substrate only (MOL); no boundary bonds
12	Leu + Thr + Ile_1 + Ile_2 + Arg + Leu_2	84	6	+1	Sidechains only; same total size as Case 1; used to quantify link-atom overhead
2	Substrate + Leu	98	1	0	
3	+ Thr_1	107	2	0	
4	+ Ile_1	121	3	0	
5	+ Ile_2	135	4	0	
6	+ Arg	154	5	+1	Arg sidechain carries charge +1
7	+ Leu_2	168	6	+1	
8	+ Phe	183	7	+1	
9	+ Thr_2	192	8	+1	
10	+ Tyr	208	9	+1	
11	+ Ile_3 (full inner shell)	222	10	+1	All first-coordination-sphere sidechains
13	First shell (4 Å, 19 residues)[b]	349	18	+1	QM/MM not computed[c]
14	Second shell (8 Å, 58 residues)[b]	822	59	+1	QM/MM not computed[c]

[a] ML atom count includes link atoms. [b] Shell residues were selected by identifying all non-Gly/Ala/Cys/Met protein sidechains with at least one heavy atom within 4 Å(Case 13) or 8 Å (Case 14) of any substrate atom; backbone atoms are excluded. [c] QM/MM reference simulations were not carried out for Cases 13 and 14 owing to the prohibitive computational cost of ωB97X/6-31G* calculations at these region sizes.

Table S3: ML/MM Simulation Throughput Benchmarked Against QM/MM

Case	ML atoms[a]	Link atoms	QM/MM (ps $hour^{-1}$)	ANI-1xnr		AIMNet2-NSE	
				ps $hour^{-1}$	Speedup[b]	ps $hour^{-1}$	Speedup[b]
12	84	6	0.019	14.59	751×	18.53	953×
1	84	0	0.021	15.79	745×	18.80	887×
2	98	1	0.021	15.83	746×	18.82	887×
3	107	2	0.021	16.15	754×	19.03	888×
4	121	3	0.021	16.11	755×	18.82	882×
5	135	4	0.021	15.91	743×	18.90	883×
6	154	5	0.021	14.75	693×	18.57	873×
7	168	6	0.021	14.72	698×	18.48	876×
8	183	7	0.021	14.56	690×	18.42	872×
9	192	8	0.021	14.67	698×	18.77	892×
10	208	9	0.021	14.68	712×	18.14	879×
11	222	10	0.020	14.59	732×	18.39	923×
13	349	18	—[c]	14.47	—[c]	17.59	—[c]
14	822	59	—[c]	14.50	—[c]	12.41	—[c]

[a] ML atom count includes link atoms. [b] Speedup = ML/MM throughput ÷ QM/MM throughput. [c] QM/MM reference data are unavailable for Cases 13 and 14 owing to the prohibitive computational cost of running ωB97X/6-31G* QM/MM simulations at these region sizes; speedup values are therefore not reported. The total simulation system comprises 75 607 atoms.

ML Region Convergence Test

To verify that the computed activation free energy barriers are insensitive to the choice of ML region boundary, we performed a systematic convergence study using MaDA-3 as the representative system. Four ML region definitions of increasing size were constructed:

- **Substrate only.** The ML region comprised the two substrate molecules (84 atoms, 0 link atoms). No protein residues were included and no ML/MM boundary bonds were introduced.
- **Catalysis-related (main-text region).** The 12 protein sidechain residues most directly involved in catalysis were added to the substrate, yielding 222 ML atoms and 12 link atoms. This is the region employed in all ML/MM MetaD simulations reported in the main text.
- **First shell.** All non-Gly/Ala/Cys/Met protein sidechains possessing at least one heavy atom within 4 Åof any substrate heavy atom were included (19 residues, 362 ML atoms, 19 link atoms). Backbone atoms were excluded throughout.
- **Second shell.** The distance threshold was extended to 8 Å, giving 58 residues, 833 ML atoms, and 58 link atoms.

For each region definition, five independent ML/MM MetaD simulations were carried out using the ANI-1xnr potential. The endo activation free energy barrier $\Delta G^{\ddagger}_{\mathrm{endo}}$ was extracted from each simulation, and the mean and standard deviation were computed across the five replicates. Protein residues outside the ML region were treated at the MM level using the AMBER ff14SB force field, with ML/MM boundary bonds capped by hydrogen link atoms placed along the C_α–C_β bond of each boundary residue. The total charge of the ML region was adjusted for each system according to the net formal charge of the included residues.

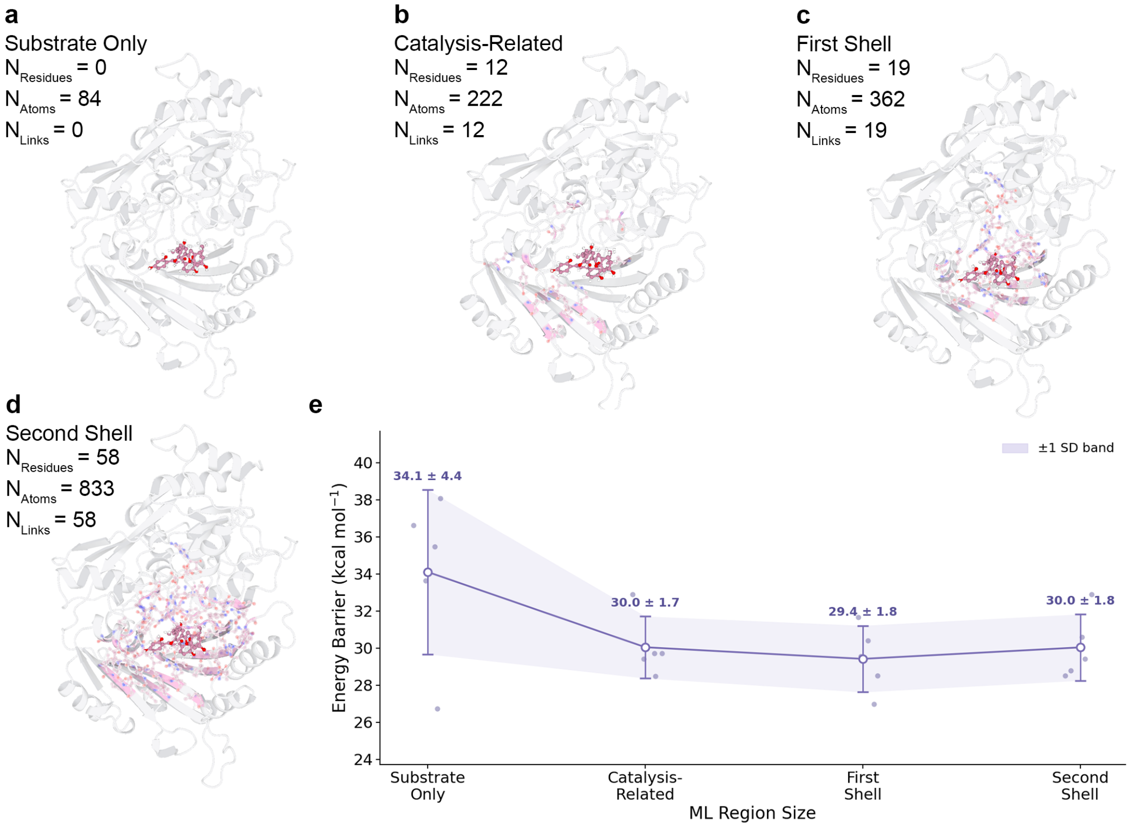

Figure S3: Convergence of the ML/MM activation barrier with respect to ML region size. **a–d**, Representative structures illustrating the four ML region definitions used in the convergence study. ML atoms are shown as sticks coloured by element; the protein backbone is rendered as a grey cartoon. N_{Residues}, N_{Atoms}, and N_{Links} denote the number of residue sidechains, total ML atoms (including link atoms), and QM/MM boundary link atoms, respectively. **e**, Computed endo activation free energy barrier ($\Delta G^{\ddagger}$, kcal mol^{-1}) for MaDA-3 as a function of ML region size. Each data point represents an independent ML/MM MetaD simulation (small circles); the large circle and error bars indicate the mean $\pm$ one standard deviation across replicates; the shaded band shows the ± 1 SD envelope across all four region sizes ($N = 5$ independent simulation replicates).

Protein sequencies for MaDA-1 and MaDA-3

Protein sequence for MaDA-1: THEAFLECLTTRIPSNSTFTPQSIIYTPDNPSYSTILDSTTQNPRFLSSSTRNPFAIITPLHASHIQAALYCSQKHGEQMRIRSGGDYEGLSYQSSVPFFILDLRNLSSISIDAKSKSAWVQAGATIGELYYGIAKTSLNLSFPGGVAHTIGVGGQLGGGGYGYSTRKYGLASDNVIDAQLIDARGRILDRKTMGEDLFWAIRGGGAGSFGIVLAWKIRLVNTPSTVTIFEAVRSWENNTTKKFIRRYQRRASKTDKDLTIFVGFRTTSSTDEEGNERISILTIVSATFHGSKDRLLQLVQKEFPDLGLVSEECTEMSWVRSIIHFNLFGDEVPLEVLLNRTLNFEMKAFKLRSDYVQKPIPDDVLEKLLSKLYDEETGXGYIEFFPYGGKMSKISESEIPFPYRAGNLYNLRYMVSWKDDGNITRTNMHLSWIKDAYDYMTPYVSKDPRGAYLNFRDLDIGVNVNESDYDYVAKASVWGTKYFRNNFYRLVDIKTIVDPTNFFKYEQSIPPLPPL

Protein sequence for MaDA-3: HESFLECLTTRISKSNSTSTPESIIYTKDNPSYSTILNSTSLNPRFFPSSARYPLLIVTPLHASHVQATVHCAKKHGIQIRIRSGGDYEGLSYMSNVTFAIVDLRNLSSIDVDVKRKSAWVQSGATLGELHYWIAKKSQNLAFPGVVGHTVGIGGMLGAGGYGYSSRKYGLSADNILDAQLIDVRGRILNRKSMGEDLFWAIRGGGAGSFGIVLAWKVRLVDVPSKVTVFTAIRDWDNNATKKFIHRYQRRIAKVDKDLTIIVRFLTASITDEKGSKKIQISTFITATYHGSQDRLLSLMEKEFPELGLLAKECAEGAWVQSILYFNLLTNSKSLDVLLNRTLNFEWRAFKIKSDYLKKPIPDQVLENLLVKLYEEDIGETFVEFFPYGGKLDEISESEIPCPHRAGNLYNLRYMVLWKEGQNATAVNKHLSWIRRAYNYMTPYVSKNPRGAFLNFRDLDIGTNPNENEINGAYNYIKQASNWGTKYFKNNFYKLIYVKTIVDPTNFFTYEQSIPSLLPH

For clarity, all MaDA-1 residues were aligned to the numbering scheme where the corresponding site is labeled as position 294 (originally 283 in the native sequence).

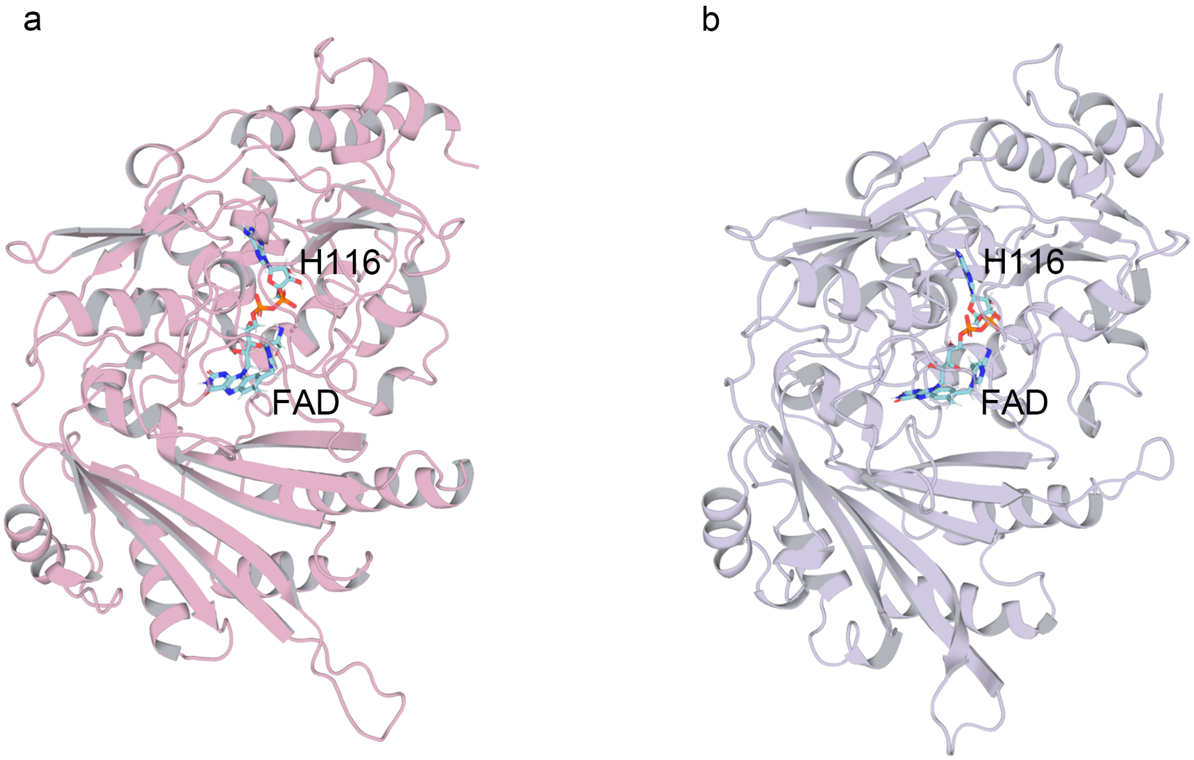

Figure S4: Overall structures of (**a**) MaDA-1 and (**b**) MaDA-3 showing the covalent linkage between residue H116 and the flavin adenine dinucleotide (FAD).

Table S4: Parameters of histidine-covalently bound flavin adenine dinucleotide (FAD).

No.	Atom	Type	M/E	i	j	k	Bond	Angle	Dihedral	Charge
1	DUMM	DU	M	0	-1	-2	0.0000	0.000	0.000	0.000 000
2	DUMM	DU	M	1	0	-1	1.4490	0.000	0.000	0.000 000
3	DUMM	DU	M	2	1	0	1.5230	111.210	0.000	0.000 000
4	N1	N	M	3	2	1	1.5400	111.208	−180.000	−0.417 500
5	H1	H	E	4	3	2	1.0300	92.003	−119.703	0.271 900
6	C1	CT	M	4	3	2	1.4510	60.971	117.717	5.174 981
7	C3	CT	3	6	4	3	1.5300	111.207	49.539	−3.117 144
8	C4	cc	S	7	6	4	1.4940	114.632	−65.986	0.226 420
9	C5	cd	B	8	7	6	1.3710	130.549	68.868	−0.232 670
10	N3	nd	S	9	8	7	1.3750	111.078	−178.411	−0.472 672
11	C6	cc	B	10	9	8	1.3130	104.791	−0.412	−0.022 172
12	N2	na	S	11	10	9	1.3610	112.513	0.503	0.378 016
13	C30	c3	3	12	11	10	1.4590	124.733	−172.992	−0.041 819
14	C28	ca	S	13	12	11	1.5220	112.134	83.147	0.077 281
15	C26	ca	B	14	13	12	1.4190	121.258	−102.237	0.157 018
16	C24	ca	B	15	14	13	1.3800	117.909	176.983	−0.372 100
17	C23	ca	S	16	15	14	1.4060	122.005	−0.003	0.467 513
18	N9	nc	S	17	16	15	1.3680	118.574	−178.827	−0.542 606
19	C20	cd	S	18	17	16	1.2930	117.889	177.624	0.198 957
20	C17	c	B	19	18	17	1.4940	118.340	−179.125	0.732 680
21	N7	ns	B	20	19	18	1.3720	112.908	177.839	−0.800 473
22	C13	c	B	21	20	19	1.4070	127.287	−1.314	0.986 180
23	N5	nc	S	22	21	20	1.3730	118.404	2.691	−0.762 119
24	C33	cd	S	23	22	21	1.3140	119.513	−0.642	0.467 667
25	N13	na	B	24	23	22	1.3620	119.084	176.897	−0.119 372
26	C11	c3	3	25	24	23	1.4700	118.275	−6.469	0.534 845

No.	Atom	Type	M/E	i	j	k	Bond	Angle	Dihedral	Charge
27	C10	c3	3	26	25	24	1.5360	112.140	90.744	−0.207 242
28	C9	c3	3	27	26	25	1.5430	109.564	−170.369	0.561 695
29	C8	c3	3	28	27	26	1.5400	112.478	−178.743	0.403 112
30	C7	c3	3	29	28	27	1.5180	112.734	64.954	−1.558 148
31	O6	os	S	30	29	28	1.4210	108.230	−179.729	−0.474 605
32	P1	p5	3	31	30	29	1.6410	116.341	−171.104	1.412 955
33	O3	o	E	32	31	30	1.4890	107.533	−166.866	−0.808 147
34	O4	o	E	32	31	30	1.5100	108.774	−33.769	−0.808 147
35	O5	os	S	32	31	30	1.6460	101.215	75.044	0.044 056
36	P2	p5	3	35	32	31	1.6380	132.113	55.795	0.507 009
37	O10	o	E	36	35	32	1.5070	103.921	171.129	−0.639 459
38	O12	o	E	36	35	32	1.4890	112.685	36.923	−0.639 459
39	O17	os	S	36	35	32	1.6590	101.422	−75.770	−0.084 483
40	C22	c3	3	39	36	35	1.4270	118.991	−82.142	0.328 170
41	C19	c5	B	40	39	36	1.5120	112.285	−100.140	0.423 189
42	C16	c5	3	41	40	39	1.5300	115.990	46.147	−0.129 213
43	C15	c5	3	42	41	40	1.5230	100.964	−160.062	0.234 821
44	C12	c5	3	43	42	41	1.5370	100.452	39.273	0.414 520
45	O16	os	E	44	43	42	1.4040	107.244	−28.501	−0.649 321
46	N12	na	S	44	43	42	1.4600	111.855	90.101	−0.189 541
47	C18	ca	S	46	44	43	1.3720	126.074	88.025	0.432 342
48	N8	nb	S	47	46	44	1.3400	128.013	1.324	−0.806 597
49	C14	ca	B	48	47	46	1.3300	111.175	−179.909	0.656 319
50	N6	nb	S	49	48	47	1.3400	128.869	0.074	−0.870 681
51	C25	ca	B	50	49	48	1.3440	118.496	−0.179	0.816 410
52	C21	ca	S	51	50	49	1.4090	118.580	0.191	−0.060 951
53	N11	nc	S	52	51	50	1.3820	132.830	179.581	−0.605 209
54	C29	cd	S	53	52	51	1.3100	103.881	−179.723	0.357 833

No.	Atom	Type	M/E	i	j	k	Bond	Angle	Dihedral	Charge
55	H32	h5	E	54	53	52	1.0830	125.595	179.979	0.069 179
56	N10	nv	B	51	50	49	1.3500	119.001	−178.320	−0.959 567
57	H30	hn	E	56	51	50	1.0090	118.146	−13.341	0.408 850
58	H31	hn	E	56	51	50	1.0080	119.131	−167.242	0.408 850
59	H20	h5	E	49	48	47	1.0870	115.951	179.995	0.025 367
60	H17	h2	E	44	43	42	1.0940	111.159	−149.987	0.104 768
61	O13	oh	S	43	42	41	1.4100	110.872	−75.233	−0.739 636
62	H22	ho	E	61	43	42	0.9690	106.031	−39.825	0.453 073
63	H21	h1	E	43	42	41	1.0970	113.501	158.400	0.053 807
64	O14	oh	S	42	41	40	1.3970	113.866	78.788	−0.631 222
65	H26	ho	E	64	42	41	0.9860	103.383	−81.638	0.486 135
66	H25	h1	E	42	41	40	1.0970	109.410	−46.148	0.051 441
67	H27	h1	E	41	40	39	1.0970	108.046	168.489	−0.019 227
68	H28	h1	E	40	39	36	1.0910	107.572	139.361	−0.136 905
69	H29	h1	E	40	39	36	1.0920	110.138	21.084	−0.136 905
70	H23	h1	E	30	29	28	1.0990	110.419	59.799	0.652 145
71	H24	h1	E	30	29	28	1.0950	110.532	−60.311	0.652 145
72	O7	oh	S	29	28	27	1.4130	108.518	−170.811	−0.704 144
73	H19	ho	E	72	29	28	0.9720	108.416	−134.254	0.549 111
74	H18	h1	E	29	28	27	1.0980	106.730	−53.018	−0.050 955
75	O8	oh	S	28	27	26	1.4100	111.703	−48.405	−0.688 436
76	H16	ho	E	75	28	27	0.9760	113.050	−67.208	0.634 368
77	H15	h1	E	28	27	26	1.0980	106.761	65.663	0.020 906
78	O9	oh	S	27	26	25	1.4100	111.852	−46.389	−0.632 103
79	H14	ho	E	78	27	26	0.9730	107.478	−40.831	0.434 002
80	H13	h1	E	27	26	25	1.0980	108.476	69.520	0.274 207
81	H11	h1	E	26	25	24	1.0900	106.608	−29.834	−0.187 717
82	H12	h1	E	26	25	24	1.0890	108.889	−145.610	−0.187 717

No.	Atom	Type	M/E	i	j	k	Bond	Angle	Dihedral	Charge
83	C32	ca	S	25	24	23	1.3870	120.445	172.596	0.033 746
84	C31	ca	S	83	25	24	1.4000	122.857	−173.474	−0.552 176
85	H10	ha	E	84	83	25	1.0800	119.841	0.640	0.637 770
86	O11	o	E	22	21	20	1.2180	118.716	−177.326	−0.637 918
87	H8	hn	E	21	20	19	1.0130	117.000	−179.338	0.397 886
88	O15	o	E	20	19	18	1.2150	124.012	−2.076	−0.588 912
89	H9	ha	E	16	15	14	1.0850	120.914	−179.581	0.213 440
90	C27	c3	3	15	14	13	1.5080	122.273	−3.434	−0.387 976
91	H33	hc	E	90	15	14	1.0920	110.371	−177.955	0.116 842
92	H34	hc	E	90	15	14	1.0950	111.825	62.488	0.116 842
93	H35	hc	E	90	15	14	1.0940	111.680	−58.177	0.116 842
94	H36	h1	E	13	12	11	1.0900	107.572	−39.463	0.048 601
95	H37	h1	E	13	12	11	1.0910	108.659	−154.520	0.048 601
96	H6	h5	E	11	10	9	1.0810	125.741	179.756	0.121 567
97	H5	h4	E	9	8	7	1.0830	126.484	2.002	0.344 943
98	H3	hc	E	7	6	4	1.0920	108.602	56.520	0.005 100
99	H4	hc	E	7	6	4	1.0900	106.494	170.251	0.005 100
100	H2	H1	E	6	4	3	1.0960	107.839	−70.728	−1.107 557
101	C2	C	M	6	4	3	1.5430	110.836	171.541	0.597 300
102	O2	O	E	101	6	4	1.2360	122.064	−113.217	−0.567 900

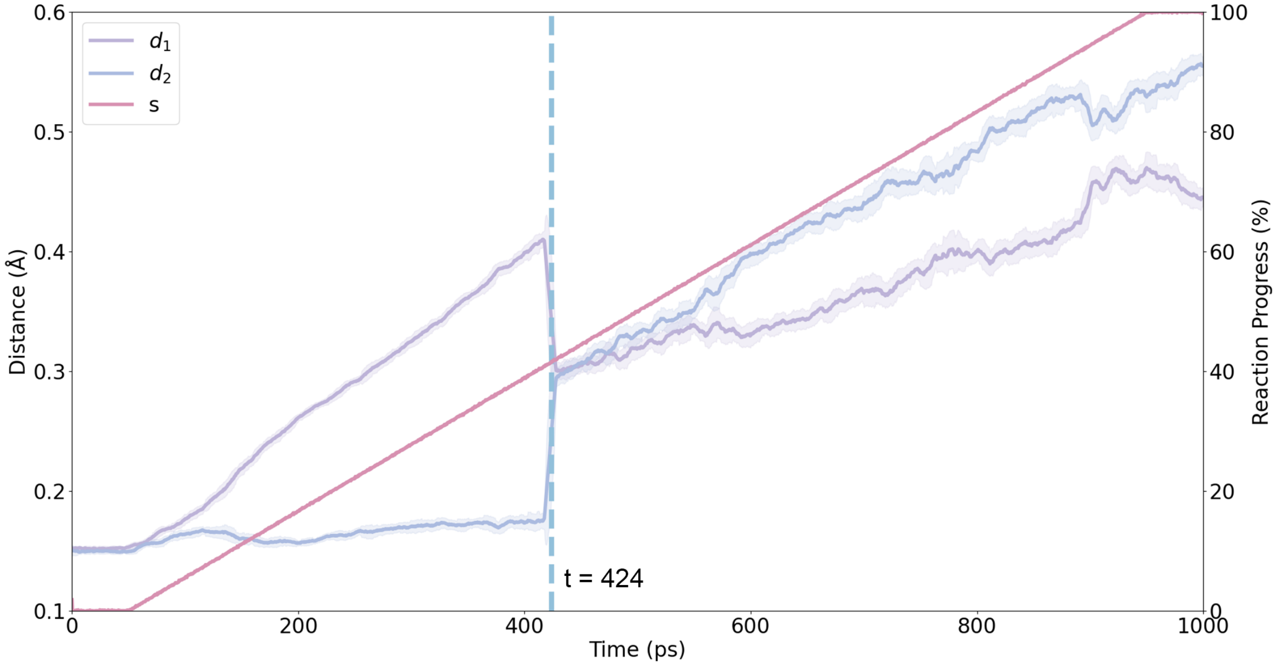

Figure S5: Steered MD validation of CV suitability. d_1 and d_2 correspond to the two forming C–C bonds, while the path-CV coordinate s denotes the progress along the reaction pathway.

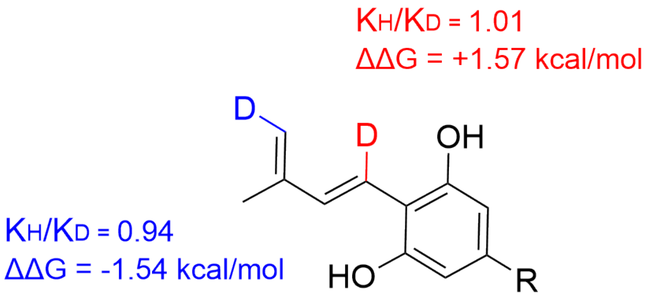

Figure S6: Kinetic isotope effects from experiment and computation. Deuterium substitution was introduced at the two carbon atoms involved in the Diels–Alder reaction. The experimental KIE was characterized by the $K_{\mathrm{H}}/K_{\mathrm{D}}$ ratio, whereas the computational $\Delta\Delta G$ values were obtained from ML/MM MetaD simulations as $\Delta G_{\mathrm{D}} - \Delta G_{\mathrm{H}}$.

Table S5: Activation free energies ($\Delta G^{\ddagger}$, kcal mol^{-1}) of MaDA1 and MaDA3 along the *endo*/*exo* pathways, as obtained from experiment, DFT cluster models, and ML/MM metadynamics. The symbol / denotes cases where no previous experimental or computational data were available. DFT QM cluster model data from Refs.[28,29] All energies are reported in kcal mol^{-1}.

	MaDA3 *endo*	MaDA3 *exo*	MaDA1 *endo*	MaDA1 *exo*	Aqueous *endo*	Aqueous *exo*
Experimental	/	19.29	16.14	/	/	/
DFT cluster	/	20.8	20.3	/	23.3	26.1
ML/MM MetaD (ANI-1xnr)	30.04	25.75	25.08	29.33	27.68	30.31
ML/MM MetaD (AIMNet2-NSE)	22.72	23.13	23.72	27.01	23.76	27.91

Table S6: Endo/Exo selectivity ratios and activation free energies ($\Delta G^{\ddagger}$, kcal mol^{-1}) of MaDA3 variants from experiment and ML/MM metadynamics simulations. The ratio refers to the relative endo:exo product distribution. $\Delta\Delta G = \Delta G_{\text{endo}} - \Delta G_{\text{exo}}$, with positive values favoring the *exo* pathway.

	MaDA3-R294G	MaDA3-R294A	MaDA3-Mu3	MaDA3-Mu5
Experimental ratio (endo:exo)	1:3.8	1:1.2	1:1	1:0.5
Computational ratio (endo:exo)	1:10.6	1:1.38	6.4:1	1:1.36
ΔG_{endo} (kcal mol^{-1})	27.27	26.88	26.69	27.50
ΔG_{exo} (kcal mol^{-1})	25.87	26.69	27.88	27.30
Experimental $\Delta\Delta G$ (kcal mol^{-1})	0.86	0.11	0	−0.45
Computational $\Delta\Delta G$ (kcal mol^{-1})	1.40	0.19	-1.19	0.20

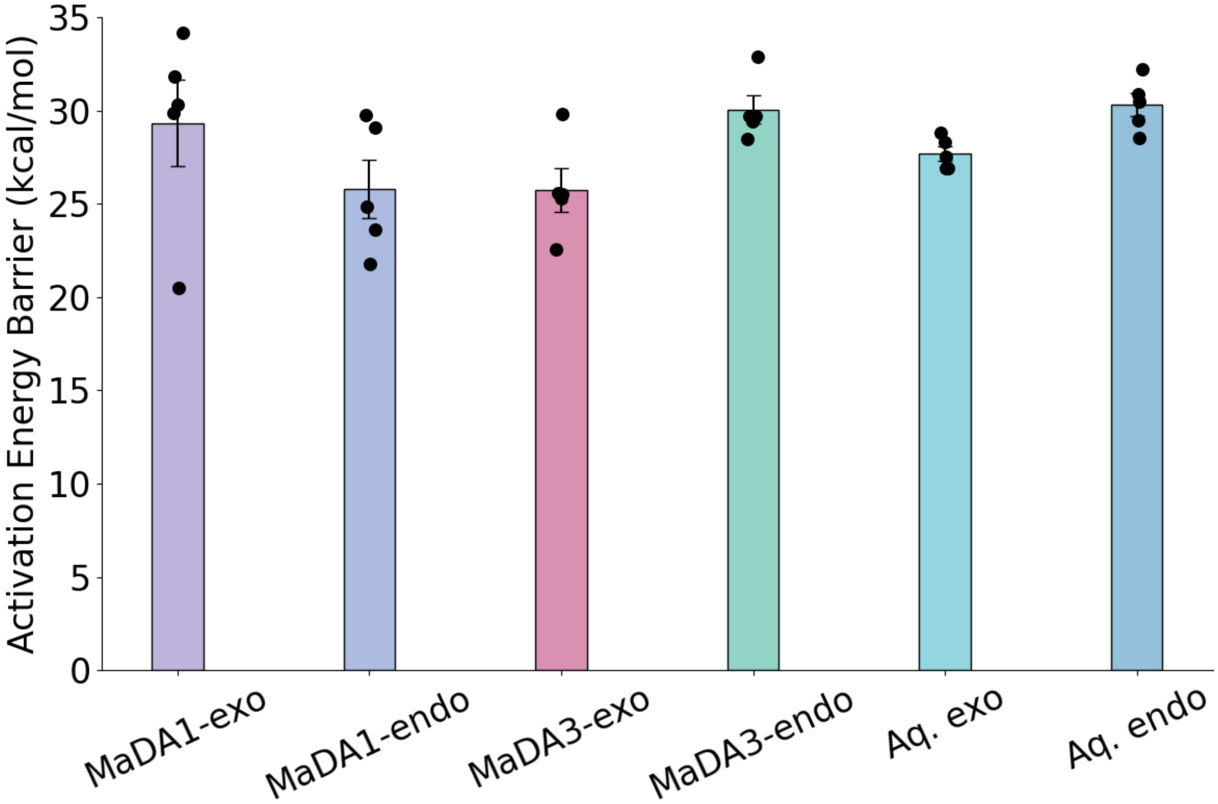

Figure S7: Activation energy barriers for Diels–Alder reactions in three environments (MaDA1, MaDA3, and aqueous solution) for two enantiomers. Each bar represents the mean barrier height, with error bars indicating the standard error of the mean ($N = 5$ independent simulation replicates).

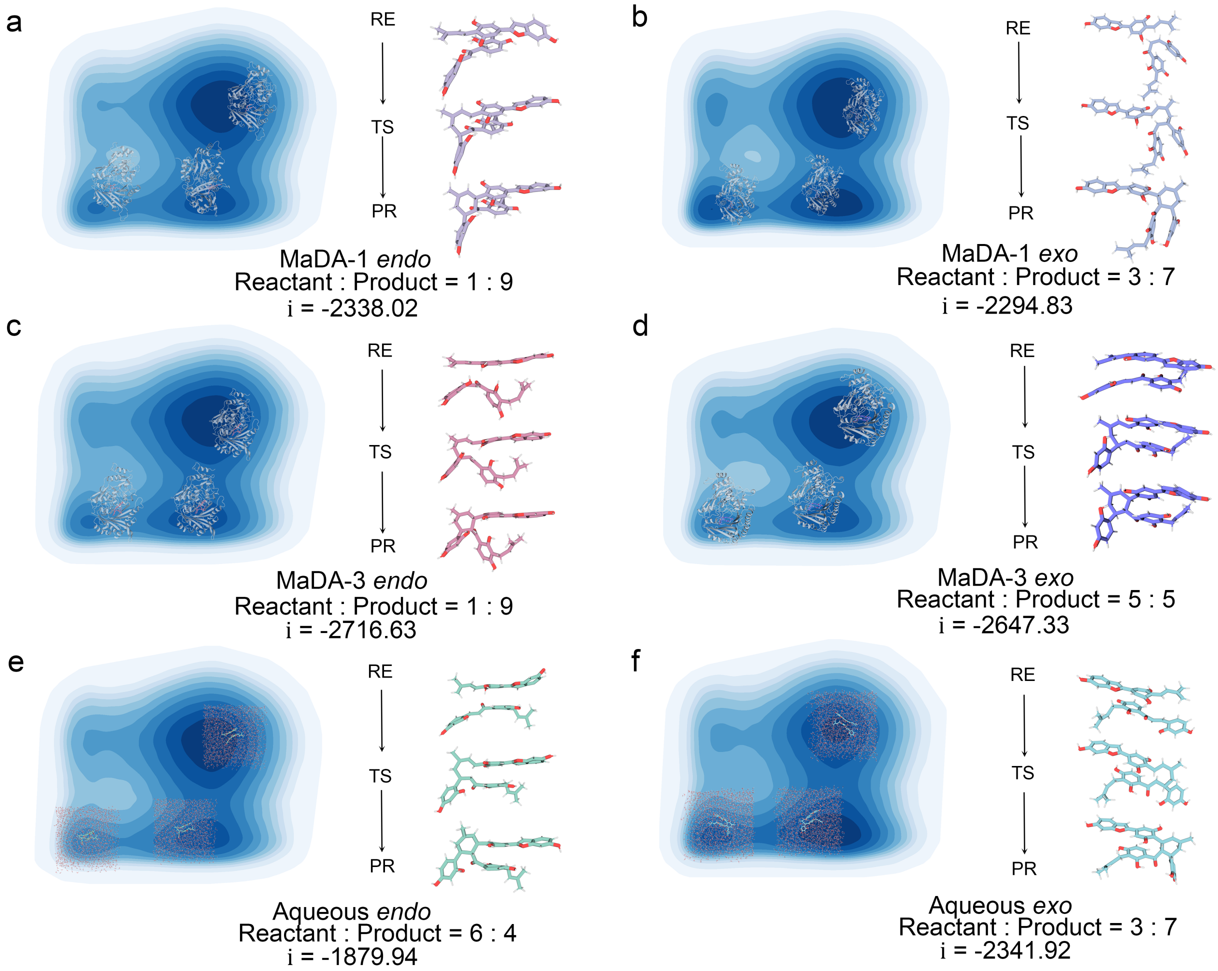

Figure S8: Committor and frequency analysis of putative TS structures. Potential TS structures and the corresponding reactant and product states identified by committor analysis are shown for MaDA-1, MaDA-3, and aqueous conditions, for both *exo* and *endo* enantiomers. For each candidate TS, ten independent trajectories were propagated, and the numbers reaching reactant or product basins are reported. The imaginary frequencies of the validated TS structures were calculated and are reported in cm^{-1}.

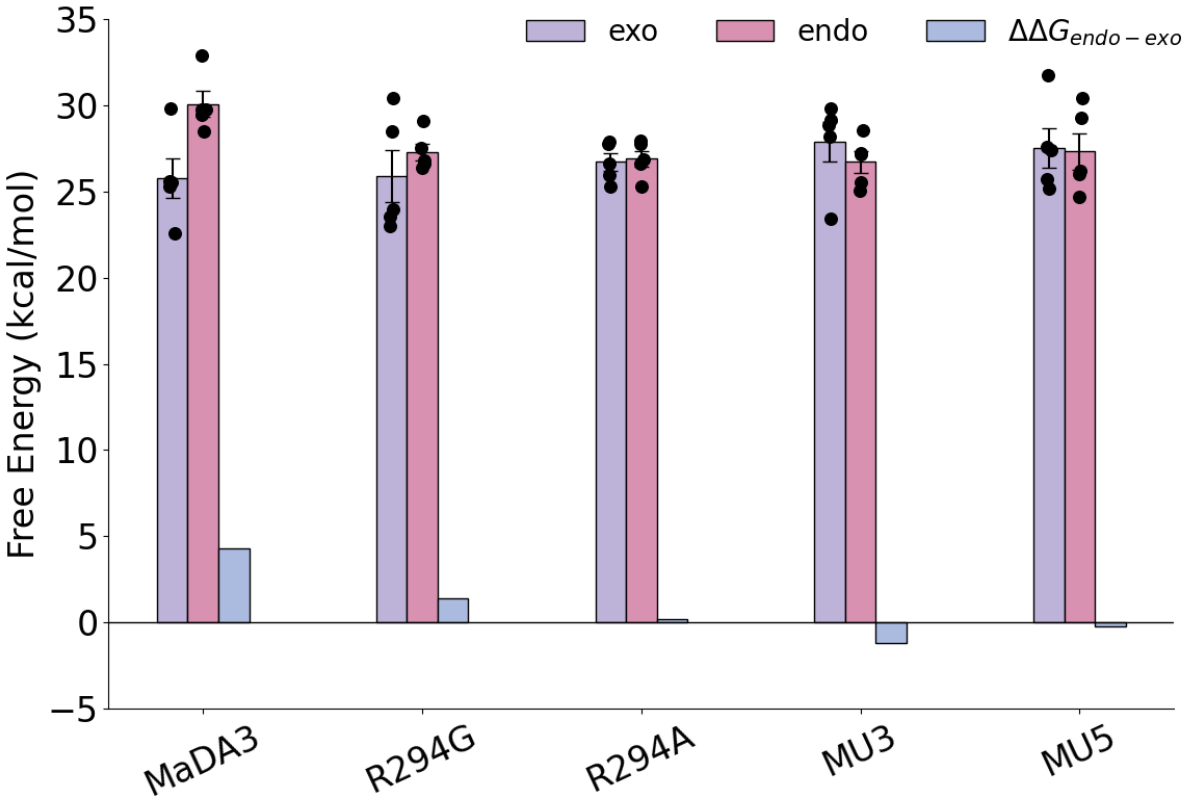

Figure S9: Comparison of free energy barriers for Diels–Alder reactions across different enzyme variants (MaDA3, R294G, R294A, MU3, and MU5). Bars represent mean activation free energies for the exo (purple) and endo (pink) pathways, with error bars indicating the standard error of the mean ($N = 5$ independent simulation replicates).. The blue bars denote the relative enantiomeric selectivity, expressed as $\Delta G_{endo} - \Delta G_{exo}$.

References

(1) Walker, R. C.; Crowley, M. F.; Case, D. A. The Implementation of a Fast and Accurate QM/MM Potential Method in Amber. *J. Comput. Chem.* **2008**, *29*, 1019–1031.

(2) Smith, J. S.; Isayev, O.; Roitberg, A. E. ANI-1: An Extensible Neural Network Potential with DFT Accuracy at Force Field Computational Cost. *Chem. Sci.* **2017**, *8*, 3192–3203.

(3) Devereux, C.; Smith, J. S.; Huddleston, K. K.; Barros, K.; Zubatyuk, R.; Isayev, O.; Roitberg, A. E. Extending the Applicability of the ANI Deep Learning Molecular Potential to Sulfur and Halogens. *J. Chem. Theory Comput.* **2020**, *16*, 4192–4202.

(4) Zhang, S.; Makoś, M. Z.; Jadrich, R. B.; Kraka, E.; Barros, K.; Nebgen, B. T.; Tretiak, S.; Isayev, O.; Lubbers, N.; Messerly, R. A.; Smith, J. S. Exploring the frontiers of condensed-phase chemistry with a general reactive machine learning potential. *Nat. Chem.* **2024**, *16*, 727–734.

(5) Kovács, D. P.; Batatia, I.; Arany, E. S.; Csányi, G. Evaluation of the MACE Force Field Architecture: From Medicinal Chemistry to Materials Science. *J. Chem. Phys.* **2023**, *159*, 044118.

(6) Kovács, D. P.; Moore, J. H.; Browning, N. J.; Batatia, I.; Horton, J. T.; Pu, Y.; Kapil, V.; Witt, W. C.; Magdău, I.-B.; Cole, D. J.; Csányi, G. MACE-OFF: Short-Range Transferable Machine Learning Force Fields for Organic Molecules. *J. Am. Chem. Soc.* **2025**, *147*, 17598–17611.

(7) Anstine, D.; Zubatyuk, R.; Isayev, O. AIMNet2: A Neural Network Potential to Meet your Neutral, Charged, Organic, and Elemental-Organic Needs. *ChemRxiv* **2023**,

(8) Kalita, B.; Zubatyuk, R.; Anstine, D. M.; Bergeler, M.; Settels, V.; Stork, C.; Spicher, S.; Isayev, O. AIMNet2-NSE: A Transferable Reactive Neural Network Potential for Open-Shell Chemistry. *Angew. Chem. Int. Ed.* **2026**, *65*, e202516763.

(9) Unke, O. T.; Chmiela, S.; Gastegger, M.; Schütt, K. T.; Sauceda, H. E.; Müller, K.-R.

SpookyNet: Learning force fields with electronic degrees of freedom and nonlocal effects. *Nat. Commun.* **2021**, *12*, 7273.

(10) Zhang, Y.; Jiang, B. FIREANN model: Universal machine learning for the response of atomistic systems to external fields. *Nat. Commun.* **2023**, *14*, 6424.

(11) Zhang, Y.; Xia, J.; Jiang, B. Physically Motivated Recursively Embedded Atom Neural Networks (REANN): Incorporating local completeness and nonlocality. *Phys. Rev. Lett.* **2021**, *127*, 156002.

(12) Zhang, Y.; Xia, J.; Jiang, B. REANN: A PyTorch-based end-to-end multi-functional deep neural network package for molecular, reactive, and periodic systems. *J. Chem. Phys.* **2022**, *156*, 114801.

(13) Smith, J. S.; Nebgen, B. T.; Zubatyuk, R.; Lubbers, N.; Devereux, C.; Barros, K.; Tretiak, S.; Isayev, O.; Roitberg, A. E. Approaching Coupled Cluster Accuracy with a General-Purpose Neural Network Potential through Transfer Learning. *Nat. Commun.* **2019**, *10*, 2903.

(14) Paszke, A. et al. PyTorch: An Imperative Style, High-Performance Deep Learning Library. Adv. Neural Inf. Process. Syst. 2019; pp 8024–8035.

(15) Kühne, T. D. et al. CP2K: An Electronic Structure and Molecular Dynamics Software Package - Quickstep: Efficient and Accurate Electronic Structure Calculations. *J. Chem. Phys.* **2020**, *152*, 194103.

(16) Becke, A. D. Density-Functional Exchange-Energy Approximation with Correct Asymptotic Behavior. *Phys. Rev. A* **1988**, *38*, 3098–3100.

(17) Lee, C.; Yang, W.; Parr, R. G. Development of the Colle–Salvetti Correlation-Energy Formula into a Functional of the Electron Density. *Phys. Rev. B* **1988**, *37*, 785–789.

(18) VandeVondele, J.; Hutter, J. Gaussian Basis Sets for Accurate Calculations on Molecular Systems in Gas and Condensed Phases. *J. Chem. Phys.* **2007**, *127*, 114105.

(19) Goedecker, S.; Teter, M.; Hutter, J. Separable Dual-Space Gaussian Pseudopotentials. *Phys. Rev. B* **1996**, *54*, 1703–1710.

(20) Grimme, S.; Antony, J.; Ehrlich, S.; Krieg, H. A Consistent and Accurate *Ab Initio* Parametrization of Density Functional Dispersion Correction (DFT-D) for the 94 Elements H–Pu. *J. Chem. Phys.* **2010**, *132*, 154104.

(21) Chai, J.; Head-Gordon, M. Long-Range Corrected Hybrid Density Functionals with Damped Atom–Atom Dispersion Corrections. *Phys. Chem. Chem. Phys.* **2008**, *10*, 6615–6620.

(22) Frisch, M. J. et al. Gaussian 16, Revision C.01. Gaussian, Inc.: Wallingford CT, 2016.

(23) Chai, J.-D.; Head-Gordon, M. Long-Range Corrected Hybrid Density Functionals with Damped Atom–Atom Dispersion Corrections. *Phys. Chem. Chem. Phys.* **2008**, *10*, 6615–6620.

(24) Ditchfield, R.; Hehre, W.; Pople, J. A. Self-Consistent Molecular-Orbital Methods. IX. An Extended Gaussian-Type Basis for Molecular-Orbital Studies of Organic Molecules. *J. Chem. Phys.* **1971**, *54*, 724–728.

(25) Wang, X.; Zhang, Y.; Sun, Z.; Zhu, R.; Asam, C.; Li, W.-L.; Wang, J. MAPLE: A General Framework for Automated Molecular Modeling across Machine-Learning Potentials. ChemRxiv, 2026.

(26) Maier, J. A.; Martinez, C.; Kasavajhala, K.; Wickstrom, L.; Hauser, K. E.; Simmerling, C. ff14SB: Improving the Accuracy of Protein Side Chain and Backbone Parameters from ff99SB. *J. Chem. Theory Comput.* **2015**, *11*, 3696–3713.

(27) Abraham, M. J.; Murtola, T.; Schulz, R.; Páll, S.; Smith, J. C.; Hess, B.; Lindahl, E. GROMACS: High Performance Molecular Simulations Through Multi-Level Parallelism from Laptops to Supercomputers. *SoftwareX* **2015**, *1–2*, 19–25.

(28) Gao, L. et al. FAD-dependent enzyme-catalysed intermolecular [4+2] cycloaddition in natural product biosynthesis. *Nat. Chem.* **2020**, *12*, 620–628.

(29) Gao, L.; Zou, Y.; Liu, X.; Yang, J.; Du, X.; Wang, J.; Yu, X.; Fan, J.; Jiang, M.; Li, Y.; Houk, K. N.; Lei, X. Enzymatic control of endo- and exo-stereoselective Diels–Alder reactions with broad substrate scope. *Nat. Catal.* **2021**, *4*, 1059–1069.
