# Supplementary material for: Multiscale machine learning molecular mechanics for mechanism and stereoselectivity of Diels-Alderase catalysis
Source: Nat Commun. 2026 May 13;17:6437. doi: 10.1038/s41467-026-72904-9 (PMC13377127; doi:10.1038/s41467-026-72904-9)
Supplement: Supplementary file 2 — Description of Additonal Supplementary Files [file 41467_2026_72904_MOESM2_ESM.pdf]

Description of Additional Supplementary Files

Supplementary Dataset 1- PDB file for MaDA1 with endo-products

Supplementary Dataset 2- PDB file for MaDA1 with exo-products

Supplementary Dataset 3- PDB file for MaDA3 with endo-products

Supplementary Dataset 4- PDB file for MaDA3 with exo-products

Supplementary Dataset 5- Forcefield parameters for FAD in AMBER format
