# Supplementary material for: Multiscale machine learning molecular mechanics for mechanism and stereoselectivity of Diels-Alderase catalysis
Source: Nat Commun. 2026 May 13;17:6437. doi: 10.1038/s41467-026-72904-9 (PMC13377127; doi:10.1038/s41467-026-72904-9)
Supplement: Supplementary file 9 — Transparent Peer Review file [file 41467_2026_72904_MOESM9_ESM.pdf]

nature portfolio

Peer Review File

Multiscale Machine Learning Molecular Mechanics for Mechanism and Stereoselectivity of Diels-Alderase Catalysis

Corresponding Author: Professor Wan-Lu Li

This file contains all reviewer reports in order by version, followed by all author rebuttals in order by version.

Version 0:

Reviewer comments:

Reviewer #1

(Remarks to the Author)
The manuscript "Redefining Computational Enzymology with Multiscale Machine Learning/Molecular Mechanics: Catalytic Mechanism and Stereoselectivity in Diels-Alderases" by Wang et al. presents an implementation of a link-atom treatment within an ML/MM framework in AMBER (MLMM4AMBER). The authors apply this framework to the Diels-Alder reaction, utilizing a multiscale approach that combines molecular dynamics, metadynamics, and commutator analysis to explore the free energy landscape. The mechanistic insights derived from these simulations, specifically regarding the asynchronous nature of the reaction and the impact of mutations on free energy barriers, are consistent with experimental data

The application of the method to the specific biological system is technically sound, and the agreement with experimental results is encouraging. It is also, to the best of my knowledge, the first application of metadynamics in an ML/MM framework (even though these simulations are carried out by an external plugin of AMBER). However, I have substantial concerns regarding (i) how the work is positioned with respect to the existing ML/MM literature, (ii) the strength of the novelty claims; particularly around the link-atom treatment and the notion of "redefining computational enzymology", and (iii) the reliance on mechanical embedding while arguing for broad generality.

In its current form, I am not yet convinced that the manuscript meets the standard of conceptual advance and clarity required for Nature Communications. Addressing the points below would require a significant re-framing of the ML/MM aspects and, in my view, additional work to demonstrate novelty beyond what is already available in the field.

Major comments

1. Positioning in the existing ML/MM literature and novelty claims

The manuscript currently cites the authors' previous ML/MM work as essentially the only prior ML/MM reference in AMBER, and then states that this is the first incorporation of a link-atom method into a reactive ML/MM framework implemented in AMBER and a broadly applicable strategy for mechanistic enzymology.

In my view, the positioning is not yet accurate or complete:

There already exist ML/MM interfaces in AMBER (e.g. EMLE, TorchANI-AMBER; 10.1021/acs.jctc.4c00248, 10.1021/acs.jpcb.5c05725, 10.1021/acs.jctc.4c01792) that provide the necessary Hamiltonian for ML/MM simulations and support link-atom treatments through the extensible SANDER interface. Moreover, the implementation described in the manuscript seems to be the standard link-atom implementation, without any tweaks or modifications relevant to the ML/MM framework.

As a result, it is difficult to assess what is truly new in this work from a methodological standpoint , as opposed to being another instance of a now-standard strategy applied to a particular system.

I would strongly encourage the authors to:

-Add a dedicated subsection in the Introduction or Theory clearly reviewing prior ML/MM implementations (including

implementations made in AMBER, as well as others) and explaining precisely what is new here (e.g. specific combination of ANI-1xnr with a particular link-atom formalism, specific technical aspects of the force redistribution -if different from standard-, the breadth of supported MLIPs, integration with MetaD, etc.).I strongly encourage a more comprehensive review of the recent ML/MM literature, as the ML/MM field has grown significantly in the last 5 years, ranging applications in a wide variety of chemical problems, and, importantly to this article, enzymatic reactions. See for example DOI: 10.1021/acs.jctc.4c01393; 10.26434/chemrxiv-2025-nw9lt for ML/MM applications in enzymatic reactions, 10.1021/jacs.4c17015 for applications in condensed-phased reactions, or 10.1002/wcms.70003, 10.48550/arXiv.2511.00951 and 10.1063/5.0260078 for reviews regarding ML/MM.

-Substantially tone down or qualify statements that this is the first link-atom ML/MM implementation in AMBER and that it “redefines” computational enzymology, unless the authors can clearly demonstrate why previous ML/MM implementations do not already provide similar capabilities. In this context, I also recommend extending the interface so it supports more sophisticated ML/MM embeddings, as ML/MM with mechanical embedding has demonstrated to accurate modelling Diels-Alder reactions but to fall short for reactions with significant electronic rearrangements (see DOI: 10.26434/chemrxiv-2025-nw9lt)

2. Statement that link atoms have historically limited ML/MM applications
The manuscript suggests that what has historically limited ML/MM applications to chemical reactions is the need for link atoms.

I find this claim difficult to reconcile with the current state of the field. My understanding is that the primary bottleneck for applying ML/MM to chemical reactions has been the availability, robustness and transferability of reactive MLIPs themselves, particularly for systems involving significant charge rearrangement, different spin states, or complex chemical spaces. Even more, as mentioned above, the use of link atoms is already supported in existing ML/MM frameworks (EMLE and TorchANI-AMBER).

I suggest the authors either (i) substantially soften this statement and reframe it as one contributing factor among others, or (ii) provide a more nuanced discussion, supported by appropriate references, explaining in what precise sense link-atom availability has been the bottleneck for ML/MM applications to reactivity. Finally, it should be clearly stated in which sense the described implementation of the link-atom method is different from standard (and different from the already existing scheme in SANDER).

3. Boundary treatment and embedding level versus claimed generality

The theoretical description in Section 2 closely parallels standard QM/MM formulations: the energy partitioning and link-atom force redistribution follow the usual pattern, and the electrostatic coupling is implemented at the mechanical embedding (ME) level, with fixed charges in the ML region and MM/ML interactions evaluated via classical Coulomb terms.

Mechanical embedding is certainly a reasonable first step and the results for MaDA are convincing. However:

- ME-level ML/MM applications have already been explored in several earlier works (just to name a few: 10.1021/acs.jctc.4c01427, 10.1021/acs.jcim.5c00033). The current state-of-art of the ME-level aims to reach the electrostatic embedding level (i.e. to take into account the effect of the environment in the ML region), both by post-hoc corrections (e.g. 10.1021/acs.jctc.4c00248, 10.1021/acs.jpcb.3c08166, 10.1021/acs.jctc.4c01792), or by directly including MM features into the ML model (eg. 10.1021/acs.jctc.5c00372, 10.1039/D4DD00295D).

- While ME seems to retrieve reasonable results for binding free energies (e.g., or even the previous work of the authors in ML/MM), recent studies (10.26434/chemrxiv-2025-nw9lt) have explicitly shown that ME reproduces electrostatic-embedding (EE) results only for fairly limited classes of reactions (e.g. when charge redistribution is modest, as in certain Diels-Alder reactions), and fails for more strongly polar or charge-transfer processes.

In this context, presenting an ME-level implementation as “redefining computational enzymology” seems too strong. To meet the novelty and generality expected for Nature Communications, I believe the authors should either:

- Demonstrate that their framework remains accurate for a broader range of enzymatic reactions, particularly those where EE or more advanced embeddings are known to be important; or

- Explicitly frame this work as a first step and significantly temper the claims about generality and “redefining” the field, clearly stating the current limitations of ME.

Minor comments:

A significant part of the manuscript is devoted to the ML/MM framework itself (support for multiple MLIPs and integration into AMBER etc.), and the Conclusions highlight that the method achieves nanosecond-timescale simulations and is broadly applicable.
Given this emphasis, I think the manuscript would benefit from more quantitative performance benchmarks. For example:
- Wall-clock timings (or ns/day) for typical MaDA simulations on the reported hardware, compared to QM/MM or to pure MM.

- Scaling with ML region size and with different MLIPs (ANI-1xnr vs AIMNet-2, MACE, etc.), at least for small representative

systems.

- Any overhead introduced by the link-atom force redistribution and electrostatic treatment relative to a ML/MM implementation without boundary handling.

(Remarks on code availability)
The code repository includes a README file, but it does not provide any installation or setup instructions, which makes it difficult to use as a standalone resource.

Reviewer #2

(Remarks to the Author)
This work explores enzyme catalysis with a newly implemented ML/MM approach. The link method is taken from the QM/MM community and applied to ML/MM simulations. The link method solves the problem of dangling bonds between the MM and ML regions by adding an additional atom to stabilize bonds. The study then applies this new implementation to Diels–Alder reactions and studies the resulting mechanisms observed. DFT and experimental values are compared to the ML/MM results.

The introduction of this new method to ML/MM computations is a useful addition to the community and this is an important problem. However, the subsequent evaluation of the method is limited in detail. There is no benchmarking of the performance of the link method itself. For example, there is no evaluation of how moving to ML/MM from an entirely ML description changes performance. Additionally, the authors do not investigate how the size of the ML region influences the results or whether the results converge with increasing ML region size. It is not clear by the end of the work how well the link method really performs or what additional error it has introduced.

Furthermore, the use of ANI-1xnr to study this system in such detail is not a good choice. ANI-1xnr is trained to periodic condensed phase systems. In this work, there are small cluster of molecules and given that ANI-1xnr does not work well in the gas phase the use of this potential needs to be justified. This should be carried out through careful evaluation of the potential in comparison to QM results for this system. Unfortunately, I expect it will be found to be highly inaccurate. Furthermore ANI-1xnr is trained on low-fidelity QM calculations which will further reduce accuracy of this potential. Given this, it is highly likely the ANI-1xnr potential is inaccurate for this system and therefore the careful evaluation of the mechanisms explored are not particularly useful.

Given these two major points, I cannot recommend publishing the paper in its current form.

To summarize, major comments:
- Additional benchmarking of the system by:
1. Comparing ML to ML/MM on small systems to evaluate the link method and ML/MM as a model.
2. Comparing varying size regions of ML in the ML/MM simulations to show convergence
- Validating the ANI-1xnr potential for the chosen system through QM calculations and ideally calculations of the barrier height for the reaction in the cluster

As an additional minor comment, much of the text is well written but the introduction is lacking a careful description of the problem they are trying to solve (dangling bonds between regions) and also does not fully describe the link method (which is the major methodological contribution). Additionally, the ANI-1xnr is not described in the methods section - both the model architecture and the training data used should be carefully outlined. As well as this, a literature review of other ML/MM methods, for example: https://pubs.acs.org/doi/abs/10.1021/acs.jctc.5c01464 should also be provided.

(Remarks on code availability)

Reviewer #3

(Remarks to the Author)
The considered work by Wang, X. et al. is a high quality systematic study of the value of ML/MM calculations for enzyme design. I have noticed a clear need for additional research in this area and am excited to see this work start the discussion. ML/MM will no doubt play an essential role in the future of enzyme design. However, there is a lack of research showing its limitations and proper implementation protocols. Overall, I find the article to be a good fit for the journal, with thoroughly described methods, and recommend it for publication. However, there are several numerical and citation inconsistencies that should be addressed before publication.

1. There is a slight discrepancy in the claim “Our ML/MM MetaD simulations reproduced these values with sub-kcal mol−1 accuracy on average” on line 244. In Table S3, the ΔG_endo is 26.69 and the ΔG_exo is 27.88, but the ΔG_endo: ΔG_exo ratio is listed as 1:6.4, which is the opposite of what the PMF indicates. Similarly, the Computational ΔΔG is listed as 1.19 where it should be –1.19. Most likely possibility is that the values in the table were swapped. If they are not swapped the main text will need to be updated in a few places to reflect this outlier in the trend.

2. The claim “absolute values exhibited a positive offset about 5 kcal mol−1 compared to theozyme and experimental

results” is not supported by the data in Table S2. MaDA3 exo the ΔG‡ is 19.29 experimentally and 25.75 computationally. Thus, an offset of 6.46. And for MaDA1 endo ΔG‡ is 16.14 experimentally and 25.08 computationally. Thus, an offset of 8.94. What I believed happened is the authors used the QM cluster model offset, which is indeed close to 5 so it would be find to update the main text statement to mention the DFT instead of the experimental values or update the offset

3. There seems to be an inconsistency in the statement “the endo pathway is thermodynamically preferred. In this case, our method yields ΔΔG = 2.63 kcal mol−1, in close agreement with the DFT estimate of 2.8 kcal mol−1”. The statement exerts that the endo pathway is preferred but a ΔΔG of +2.63 would indicate a higher barrier for the endo pathway based on the definition of ΔΔG in Table S3, ΔΔG = ΔGendo – ΔGexo. Looking at Table S2, it should be negative, endo – exo.

4. There is an arithmetic error in Figure 2e, where the |d1–d2| is computed as 0.49 where It should be 0.39. Similarly, the d3 value is not labeled on the molecular structures on the Figure 2e and 2d plots.

5. The authors have been meticulous in their citations hygiene. However, there are two places that could lead to confusion. In Table S2, DFT QM cluster model data is provided with no attributing citations. While there are citations in the main text, someone reviewing only the SI may attribute this data to this publication. Similarly, in Figure 4, experimental values from different publications are provided with no accompanying citations in the figure or caption. While there are citations in the main text, sometimes figures are shown separately from the text during presentations/reviews which could lead to misattribution of the data.

6. In the sentence, “To balance accuracy and efficiency, Walsh et al. introduced the hybrid QM/MM framework” the authors attribute the discovery to Walsh whereas it should be to Warshel. Levitt could also be mentioned.

7. The authors claim “the link-atom method (Fig. 1c) remains the simplest and most practical choice, yet it has never been systematically implemented in ML/MM simulations of enzymatic reactivity”. I would remove this claim as a simple search returns a few previous demonstrations of ML/MM link atom demonstrations (e.g., ML/MM toolkit – Towards Accelerated Mechanistic Investigation of Enzymatic Reactions)

(Remarks on code availability)

Version 1:

Reviewer comments:

Reviewer #1

(Remarks to the Author)
I thank the authors for their thorough and thoughtful revision. The manuscript has been substantially improved in response to all three reviewers' comments. I particularly appreciated the ML region convergence study, which provides a pragmatic and convincing response to the mechanical embedding limitation: by systematically expanding the ML region to include successive shells of active-site residues, the authors demonstrate that polarization effects are captured directly within the MLIP rather than through the embedding scheme, and that the computed barriers are well-converged beyond the catalysis-related region. This is a sensible strategy that sidesteps the need for electrostatic embedding in the present context. The remaining changes (comprehensive literature review, revised novelty claims, performance benchmarks, etc.) collectively address the other concerns I raised in my initial review. I am now satisfied that the work is suitable for publication in Nature Communications. I have only two minor observations that the authors may wish to consider during the proofing stage:

1) Performance scaling interpretation. The authors state that "throughput is essentially independent of ML region size up to at least 222 atoms, confirming that the MLIP evaluation rather than the boundary treatment dominates the computational cost." I believe this interpretation is slightly inverted: the fact that throughput is flat up to ~222 atoms suggests that the MLIP evaluation is not yet the bottleneck in that regime, and that some other component (e.g., the MM evaluation or the coupling overhead) is rate-limiting. The MLIP cost appears to become relevant only at larger region sizes. This does not affect any conclusion of the paper, but a small rephrasing would be more precise.

2)Link atom treatment across architectures. The new SI section "Universal Link Atom Treatment Across Heterogeneous MLIP Architectures" provides a detailed and pedagogically valuable exposition. However, I note that in practice, the MLIP simply receives a capped molecular cluster containing a hydrogen atom at the link position: the architecture-specific descriptions document the normal internal operation of each MLIP when presented with that hydrogen, rather than any modification to the models themselves. The authors may wish to clarify this distinction so that readers do not overinterpret the complexity of the link atom dispatch as implying architecture-specific algorithmic changes.

3) SI figure numbering. I noticed a discrepancy between the figure numbers referenced in the main text and those in the Supporting Information (e.g., the ML region convergence figure is referred to as Figure S4 in the main text but labeled Figure S3 in the SI). I would recommend the authors carefully check all SI figure cross-references for consistency before final publication.

Neither point requires additional calculations or a further revision cycle.

(Remarks on code availability)
The code is hosted on GitHub (https://github.com/ClickFF/MLMM4AMBER) and includes a README file, detailed installation instructions (INSTALL.md), and a usage tutorial with example workflows. I did not install or run the code myself, but the installation procedure is well documented and the primary complexity lies in compiling AMBER itself, which serves as the backend MD engine and is a prerequisite. The authors have addressed the repository documentation concerns raised during the first round of review.

Reviewer #2

(Remarks to the Author)
The analysis of the ML/MM region is greatly improved and was required. The analysis of ANI-1xnr shows that this is a source of error and better potentials are available. I don't think that stating that when the project started this was the best available potential is a justification for the choice.

(Remarks on code availability)

Reviewer #3

(Remarks to the Author)
I thank the authors for the care they took in addressing my suggestions. I have reviewed the responses and all of my suggestions have been addressed completely.

(Remarks on code availability)

Open Access This Peer Review File is licensed under a Creative Commons Attribution 4.0 International License, which permits use, sharing, adaptation, distribution and reproduction in any medium or format, as long as you give appropriate credit to the original author(s) and the source, provide a link to the Creative Commons license, and indicate if changes were made.
In cases where reviewers are anonymous, credit should be given to 'Anonymous Referee' and the source.
The images or other third party material in this Peer Review File are included in the article's Creative Commons license, unless indicated otherwise in a credit line to the material. If material is not included in the article's Creative Commons license and your intended use is not permitted by statutory regulation or exceeds the permitted use, you will need to obtain permission directly from the copyright holder.
To view a copy of this license, visit https://creativecommons.org/licenses/by/4.0/

Response Letter

We sincerely appreciate the reviewers' comments and suggestions, which have greatly improved the paper. Below are our responses. The italicized sections correspond to new/updated discussions, which are also highlighted in the revised manuscript.

Response to Reviewer 1:

Recommendation: The manuscript "Redefining Computational Enzymology with Multiscale Machine Learning/Molecular Mechanics: Catalytic Mechanism and Stereoselectivity in Diels-Alderases" by Wang et al. presents an implementation of a link-atom treatment within an ML/MM framework in AMBER (MLMM4AMBER). The authors apply this framework to the Diels-Alder reaction, utilizing a multiscale approach that combines molecular dynamics, metadynamics, and commutator analysis to explore the free energy landscape. The mechanistic insights derived from these simulations, specifically regarding the asynchronous nature of the reaction and the impact of mutations on free energy barriers, are consistent with experimental data

The application of the method to the specific biological system is technically sound, and the agreement with experimental results is encouraging. It is also, to the best of my knowledge, the first application of metadynamics in an ML/MM framework (even though these simulations are carried out by an external plugin of AMBER). However, I have substantial concerns regarding (i) how the work is positioned with respect to the existing ML/MM literature, (ii) the strength of the novelty claims; particularly around the link-atom treatment and the notion of "redefining computational enzymology", and (iii) the reliance on mechanical embedding while arguing for broad generality.

In its current form, I am not yet convinced that the manuscript meets the standard of conceptual advance and clarity required for Nature Communications. Addressing the points below would require a significant re-framing of the ML/MM aspects and, in my view, additional work to demonstrate novelty beyond what is already available in the field.

Question 1. Positioning in the existing ML/MM literature and novelty claims

The manuscript currently cites the authors' previous ML/MM work as essentially the only prior ML/MM reference in AMBER, and then states that this is the first incorporation of a link-atom method into a reactive ML/MM framework implemented in AMBER and a broadly applicable strategy for mechanistic enzymology.

In my view, the positioning is not yet accurate or complete:

There already exist ML/MM interfaces in AMBER (e.g. EMLE, TorchANI-AMBER; 10.1021/acs.jctc.4c00248, 10.1021/acs.jpcb.5c05725, 10.1021/acs.jctc.4c01792) that provide the necessary Hamiltonian for ML/MM simulations and support link-atom treatments through the extensible SANDER interface. Moreover, the implementation described in the

manuscript seems to be the standard link-atom implementation, without any tweaks or modifications relevant to the ML/MM framework.

As a result, it is difficult to assess what is truly new in this work from a methodological standpoint , as opposed to being another instance of a now-standard strategy applied to a particular system.

I would strongly encourage the authors to:

-Add a dedicated subsection in the Introduction or Theory clearly reviewing prior ML/MM implementations (including implementations made in AMBER, as well as others) and explaining precisely what is new here (e.g. specific combination of ANI-1xnr with a particular link-atom formalism, specific technical aspects of the force redistribution -if different from standard-, the breadth of supported MLIPs, integration with MetaD, etc.).I strongly encourage a more comprehensive review of the recent ML/MM literature, as the ML/MM field has grown significantly in the last 5 years, ranging applications in a wide variety of chemical problems, and, importantly to this article, enzymatic reactions. See for example DOI: 10.1021/acs.jctc.4c01393; 10.26434/chemrxiv-2025-nw9lt for ML/MM applications in enzymatic reactions, 10.1021/jacs.4c17015 for applications in condensed-phased reactions, or 10.1002/wcms.70003, 10.48550/arXiv.2511.00951 and 10.1063/5.0260078 for reviews regarding ML/MM.

-Substantially tone down or qualify statements that this is the first link-atom ML/MM implementation in AMBER and that it "redefines" computational enzymology, unless the authors can clearly demonstrate why previous ML/MM implementations do not already provide similar capabilities. In this context, I also recommend extending the interface so it supports more sophisticated ML/MM embeddings, as ML/MM with mechanical embedding has demonstrated to accurate modelling Diels-Alder reactions but to fall short for reactions with significant electronic rearrangements (see DOI: 10.26434/chemrxiv-2025-nw9lt)

Response: We sincerely thank the reviewer for this thorough and constructive assessment. We recognize that the ML/MM field has evolved rapidly in recent years, and we have strengthened the manuscript to more clearly situate the present work within this broader context.

In the revised manuscript, we have made the following changes:

(1) Comprehensive literature review. We have added a dedicated subsection in the Introduction reviewing prior ML/MM implementations across multiple platforms. We now classify existing approaches along two axes, embedding scheme (mechanical, polarization-corrected, and electrostatic embedding) and software integration strategy (compiled vs. socket-based), and cite the key implementations:

Within AMBER: emle-engine (Zinovjev et al., JCTC 2024, 10.1021/acs.jctc.4c00248),

TorchANI-Amber (Pickering et al., JPCB 2025, 10.1021/acs.jpcb.5c05725), and the PCME extension by Semelak et al. (JCTC 2025, 10.1021/acs.jctc.4c01792).

Outside AMBER: GENESIS-based ML/MM (Lei et al., JCTC 2025, 10.1021/acs.jctc.4c01393), AMP framework in GROMOS (Pultar et al., JACS 2025, 10.1021/jacs.4c17015).

Reviews: Swiderek et al. (WIREs 2025, 10.1002/wcms.70003), Andreichev et al. (arXiv:2511.00951), Grassano et al. (Chem. Phys. Rev. 2025, 10.1063/5.0260078).

Enzymatic ML/MM applications: Zinovjev et al. (ChemRxiv 2025, 10.26434/chemrxiv-2025-nw9lt) demonstrated Diels-Alder reactions; Lei et al. (JCTC 2025, 10.1021/acs.jctc.4c01393) with continual learning for enzyme variants.

(2) Honest repositioning of novelty claims. We have substantially revised the manuscript to: (a) Remove the claim of being the "first link-atom ML/MM implementation in AMBER." (b) Replace "redefining computational enzymology" with more measured language - "advancing". (c) Explicitly acknowledge that emle-engine and TorchANI-Amber already provide ML/MM capabilities in AMBER. (d) We have also clarified what distinguishes the present framework from prior implementations: the support for a broad range of interchangeable MLIP backends (ANI-1xnr, AIMNet2-NSE, MACE, and other potentials) within a single unified codebase, and the tight integration with PLUMED that enables metadynamics-based enhanced sampling directly within the ML/MM framework. Importantly, the framework specifically adopts ANI-1xnr and AIMNet2-NSE as state-of-the-art universal reactive potentials that require no system-specific training, meaning that experimental researchers can directly apply reactive ML/MM simulations within AMBER without the need to develop or train a bespoke potential, a prerequisite that has historically restricted such approaches to specialized computational groups.

(3) Clear delineation of what is new. We acknowledge that the geometric positioning of the link atom and the chain-rule force redistribution follow standard QM/MM formalism shared with all link-atom implementations. A detailed discussion of the link-atom treatment, including its historical context, the specific challenges it poses in a reactive ML/MM framework, and the technical contributions of our implementation, is provided in our response to Question 2 below.

The corresponding comprehensive literature review on ML/MM development across different software packages and embedding schemes has been added to the Introduction from Page 4, Line 42 to Line 55:

"Building on this, the ML/MM framework replaces the QM region with MLIPs, achieving both efficiency and accuracy. In recent years,ML/MM has been implemented across several mainstream MD software platforms, including GROMOS[15,16], GENESIS[17], and AMBER[18-21]. A central challenge common to all ML/MM implementations is the treatment of the coupling between the ML and MM subsystems. The most simplest approach, mechanical embedding (ME), evaluates the ML region in vacuo and adds classical non-bonded interactions between subsystems; this strategy has been employed in early AMBER-based ML/MM work[18,20]. However, ME ignores the polarisation of the ML region by the MM environment. Therefore, a more advanced

polarisation-corrected mechanical embedding (PCME) schemes have been developed by Semelak et al.[21]*; subsequently, Zinovjev et al.*[19,22] *introduced an electrostatic embedding. Beyond the embedding scheme, progress in ML/MM spans long-range electrostatic treatments, model architectures, and coupling strategies; comprehensive accounts of these developments can be found in recent reviews*[23–25]*.”*

Page 5, Line 71 to Line 84:

“Here we report the incorporation of the link-atom method into a reactive ML/MM framework implemented within AMBER[33,34]*, establishing a practical strategy for treating covalent boundaries while enabling seamless coupling of reactive MLIPs with molecular mechanics environments. A central aspect of this implementation is the demonstration that a single link atom module can interface with MLIPs built on fundamentally different representation paradigms, including ANI, MACE, AIMNet2, SpookyNet and EANN, providing a unified and backend-agnostic codebase in which the active ML potential can be exchanged without modification to the boundary treatment or the broader simulation infrastructure. Crucially, the framework adopts state-of-the-art universal reactive potentials, in particular ANI-1xnr*[26] *and AIMNet2-NSE*[27]*, which are directly deployable within AMBER without any system-specific training, removing the barrier that has historically limited the accessibility of reactive ML/MM simulations to groups with dedicated potential development expertise. Furthermore, the framework is interfaced with PLUMED*[35,36]*, enabling enhanced sampling methods such as metadynamics to be applied directly within the ML/MM environment across all supported MLIP backends.”*

Question 2. Statement that link atoms have historically limited ML/MM applications
The manuscript suggests that what has historically limited ML/MM applications to chemical reactions is the need for link atoms.

I find this claim difficult to reconcile with the current state of the field. My understanding is that the primary bottleneck for applying ML/MM to chemical reactions has been the availability, robustness and transferability of reactive MLIPs themselves, particularly for systems involving significant charge rearrangement, different spin states, or complex chemical spaces. Even more, as mentioned above, the use of link atoms is already supported in existing ML/MM frameworks (EMLE and TorchANI-AMBER).

I suggest the authors either (i) substantially soften this statement and reframe it as one contributing factor among others, or (ii) provide a more nuanced discussion, supported by appropriate references, explaining in what precise sense link-atom availability has been the bottleneck for ML/MM applications to reactivity. Finally, it should be clearly stated in which sense the described implementation of the link-atom method is different from standard (and different from the already existing scheme in SANDER).

Response: We thank the reviewer for this important comment. We agree that the availability and transferability of reactive MLIPs have been the primary enabling factor for reactive ML/MM. We have revised the manuscript to reflect this perspective and now frame

boundary treatment as a complementary challenge that becomes critical once transferable reactive MLIPs are available.

Regarding the reviewer's comment that link-atom support already exists in EMLE and TorchANI-AMBER: we have carefully examined the references cited by the reviewer. In their implementation, the EMLE packages themselves and the associated ML/MM studies do not explicitly describe or demonstrate a link-atom implementation at the ML/MM level. Specifically, in the enzymatic application by Zinovjev et al. (ChemRxiv 2025, 10.26434/chemrxiv-2025-nw9lt), only the substrate is treated by the MLIP, and the chosen reactions, the Diels-Alder cycloaddition in AbyU and the Claisen rearrangement of chorismate to prephenate, <u>are cases in which no protein residue directly participates in bond making or breaking; the protein provides an electrostatic environment but the chemistry is confined entirely to the substrate.</u> These observations are consistent with the absence of an operational link-atom scheme in these frameworks: if link atoms were available and validated, one would expect the ML region to include catalytically relevant residues. We note this not to diminish these excellent contributions, but to clarify the specific gap that our work addresses, and we also soften our tone in the description of link-atom implementation. That said, we fully accept the reviewer's point that our original framing was too strong. In the revised manuscript, we have:

(1) Reframed the bottleneck discussion: We now present reactive MLIP availability as the historically primary bottleneck, and the boundary treatment as a second, complementary challenge that becomes relevant once reactive MLIPs are available.

(2) Clarified why our implementation is non-trivial despite following the standard link-atom formalism: While the geometric positioning and force redistribution (Eqs. 1–4 in the SI) follow the established QM/MM formalism, the way the link atom interacts with each MLIP's internal representation differs fundamentally across architectures. In ANI, the link atom is an additive perturbation to species-resolved symmetry functions; in MACE, it is a first-class node in the E(3)-equivariant molecular graph participating in up to 13-body correlations via Clebsch-Gordan-coupled spherical harmonics; in AIMNet2, it not only enters scalar/vector message-passing convolutions but also alters the global electronic state through Neural Charge Equilibration, requiring correct propagation of total charge Q and spin multiplicity S; in SpookyNet, it participates through four distinct channels (nuclear embedding, electronic state attention, local s/p/d interactions, and global nonlocal self-attention unbounded by distance); in EANN, it contributes to a GTO-based squared density that encodes three-body angular correlations implicitly. A unified framework that correctly handles this diversity, including heterogeneous input tensor formats, architecture-specific species validation, unit conversions, and global electronic state management, through a single compiled dispatch layer constitutes a genuine engineering and validation contribution beyond the standard QM/MM link atom implementation. A detailed mathematical exposition with full equations for all five architectures, along with a summary comparison table, is provided in the new Supplementary Information document "Universal Link Atom Treatment Across Heterogeneous MLIP Architectures"

The corresponding introduction are from Page 5, Line 71 to Line 72:
"*Here we report the incorporation of the link-atom method into a reactive ML/MM framework implemented within AMBER.*"

The Sections added to Supporting Information are from Page S2 to Page S9:
"***Universal Link Atom Treatment Across Heterogeneous MLIP Architectures***

A central aspect of the present implementation is that a single link atom module interfaces with MLIPs built on fundamentally different representation paradigms. Unlike traditional QM/MM[1], where the link atom merely adds basis functions to a Fock matrix, each MLIP architecture processes the link atom through a qualitatively distinct computational pathway determined by its descriptor extraction scheme. Below, we first describe the shared positioning and force redistribution formalism, then detail how the link atom enters the descriptor space of each of the five supported architectures, including ANI, MACE, AIMNet2, SpookyNet, and EANN, highlighting the unique challenges in each case.

Shared Formalism: Link Atom Positioning and Force Redistribution

The geometric positioning and chain-rule force redistribution are common to all architectures and inherited from the established QM/MM link atom formalism[1]. When a covalent bond between a QM-region atom Q and an MM-region atom M is severed, a link atom L is placed along the $Q - M$ bond vector:

$$r_L = r_Q + \frac{d_L}{|r_M - r_Q|}(r_M - r_Q) \quad (1)$$

where $d_L = 1.09$ Å *is the equilibrium C--H bond length. With* $e = (r_M - r_Q)/R$ *and* $R = |r_M - r_Q|$, *the force redistribution reads:*

$$F_L^{\perp} = F_L - (F_L \cdot e)\, e \quad (2)$$

$$F_M^{mod} = \frac{d_L}{R} F_L^{\perp} \quad (3)$$

$$F_Q += F_L - F_M^{mod}, \quad F_M += F_M^{mod} \quad (4)$$

Our contribution lies in how the force F_L *is computed by the MLIP, a process that differs fundamentally across architectures, as detailed below.*

ANI: Link Atom in Symmetry Function Descriptors

The ANI family[2-4] decomposes the total energy into atomic contributions via species-specific neural networks: $E = \sum_i NN^{Z_i}(G_i^{Z_i})$, *where* $G_i^{Z_i}$ *is the Atomic Environment Vector (AEV) consisting of species-resolved radial and angular sub-vectors.[2] The link atom L (species H) modifies the AEVs of every ML-region atom* i *within the cutoff:*

Radial modification. *For any QM atom* i *with* $R_{iL} < R_c^{rad}$, *L contributes to the hydrogen radial sub-AEV:*

$$G_{i,m}^{R,H} += exp[-\eta_R (R_{iL} - R_s^{(m)})^2]\; f_c(R_{iL}) \quad (5)$$

where $f_c(R) = \frac{1}{2}cos(\pi R/R_c) + \frac{1}{2}\}$ *for* $R < R_c$*(zero otherwise), and* m *indexes the radial shift parameters* $R_s^{(m)}$.

Angular modification. *For any QM atom* i *and neighbor* j *both within* R_c^{ang} *of* i*, the triplet* (j, i, L) *contributes to the angular sub-AEV* $G_i^{A,(Z_j,H)}$*:*

$$G_{i,m}^{A,(Z_j,H)} += 2^{1-\zeta}(1 + cos(\theta_{jiL} - \theta_s)\backslash bigr)^{\zeta} \ \ exp\left[-\eta_A \left(\frac{R_{ij}+R_{iL}}{2} - R_s\right)^2\right] f_c(R_{ij})\, f_c(R_{iL}) \quad (6)$$

where θ_{jiL} *is the angle at center atom* i *between neighbors* j *and* L.

Link atom's own AEV. *The link atom has its own AEV* G_L^H *constructed from all QM neighbors within its cutoff. The atomic energy* $E_L = NN^H(G_L^H)$ *contributes to the total ML energy, and the force* $F_L = -\nabla_{r_L} E$ *is redistributed via Eqs.2-4.*

MACE: Link Atom as a Graph Node in Equivariant Convolutions

MACE[5,6] *builds on the Atomic Cluster Expansion (ACE) framework, constructing body-ordered equivariant features through iterated message passing on a molecular graph* $\mathcal{G} = (\mathcal{V}, \mathcal{E})$.[5] *Unlike ANI, the link atom is not merely a radial perturbation, it is a first-class node in the molecular graph with profound consequences:*

(a) Edge construction and species encoding. *The link atom* L *participates in edge enumeration:*

$$\mathcal{E} = \{(i,j) \ \mid \ |r_i - r_j| < rcut, \ i,j \in \{1, \ldots, N_Q + N_L\}\} \quad (7)$$

generating edges to/from all QM atoms within $rcut$. L *receives the hydrogen embedding:* $h_{L,k00}^{(0)} = W_{k,Z=1}$*, providing species-specific learned features that influence all downstream computations.*

(b) Contribution to the one-particle basis. *In the first message-passing layer,* L *contributes to the atomic basis of every atom* i *with* $r_{iL} < rcut$*:*

$$A_{i,klm}^{(1)} += R_{kl}^{(1)}(r_{iL}) \ \ Y_l^m(\hat{r}_{iL}) \ \ W_{k,Z_H}^{(1)} \quad (8)$$

where $R_{kl}^{(1)}$ *are learnable radial functions expanded in Bessel basis,* Y_l^m *are real spherical harmonics, and* $W_{k,Z_H}^{(1)}$ *are species-dependent learnable weights. This contribution propagates into all body-ordered features via the symmetric contraction. For correlation order* $\nu = 3$ *(four-body terms), the link atom enters cross-terms of the form:*

$$B_i^{(\nu)} \ni \mathcal{C} \cdot A_i^{[from\ L]} \cdot A_i^{[from\ j]} \cdot A_i^{[from\ k]} \quad (9)$$

encoding angular correlations between the link atom and pairs of QM atoms through

Clebsch--Gordan (CG) coupled spherical harmonics, a many-body equivariant interaction.

(c) Multi-hop message propagation. *Through* T *layers of message passing, the link atom's influence propagates to atoms up to* $T \times rcut$ *away. At each layer, the link atom both sends equivariant messages (shaped by its species weight and local geometry) and receives messages from QM neighbors, enabling iterative refinement of its feature representation.*

(d) Effective body order. *With* $\nu = 3$ *and* $T = 2$*, the effective body order is* $(\nu^{T+1} - 1)/(\nu - 1) = (3^3 - 1)/(3 - 1) = 13$*. The link atom participates in up to 13-body correlations through the iterated symmetric contraction, a far richer interaction than the 3-body terms captured by ANI's angular symmetry functions.*

AIMNet2: Link Atom in Message Passing with Charge Equilibration

AIMNet2[7,8] is an invariant message-passing neural network that decomposes the total energy into local, dispersion, and Coulomb contributions.[7] The link atom participates in both geometric and electronic channels:

(a) Scalar/vector convolution. *The link atom contributes to every QM atom's scalar embedding through the hydrogen 2D embedding:*

$$v_{isd} \mathrel{+}= a_{ds}(Z_H) \odot g_{i,L,s} \quad (10)$$

where $a_{ds}(Z_H) \in \mathbb{R}^{d\times s}$ *is the learnable 2D embedding matrix for hydrogen,* $g_{i,L,s} = exp[-\eta_s(r_{iL} - r_s)^2]\, f_c(r_{iL})$ *is the Gaussian radial basis, and* $\odot$ *denotes the Hadamard product. It similarly contributes to the vector embedding, encoding the angular direction* $\hat{r}_{iL}$ *from atom* i *to the link atom.*

(b) Iterative feature refinement. *Through* T *iterations of message passing, the link atom both sends and receives feature updates, allowing its representation to adapt to the local QM-region environment.*

(c) Critical: Charge equilibration with link atoms. *This is the most consequential aspect of the link atom in AIMNet2. When a link atom is added, it modifies the effective electron count of the ML subsystem. The Neural Charge Equilibration (NQE) constraint enforces* $\sum_i \tilde{q}_i = Q$*, where the sum now includes the link atom:*

$$\tilde{q}_i = q_i + f_i \cdot \left(Q - \sum_{j=1}^{N} q_j\right) \quad (11)$$

where q_i is the raw predicted charge and f_i *is a neural-network-predicted weight analogous to the Fukui function (*$\sum_i f_i = 1$*). The link atom acquires a non-trivial partial charge* $\tilde{q}_L$ *through this redistribution mechanism. For AIMNet2-NSE (Neural Spin Equilibration),[8] both the total charge* Q *and spin multiplicity* S *must correctly reflect the ML subsystem including the link atom:*

$$\tilde{q}_i^\alpha = q_i^\alpha + f_i^\alpha \cdot (Q^\alpha - \textstyle\sum_j q_j^\alpha), \quad Q^\alpha = 0.5 \cdot (Q - S + 1) \quad (12)$$

SpookyNet: Link Atom in Nuclear/Electronic Embedding with Nonlocal Interactions

SpookyNet[9] *is the only architecture in our framework that employs both distance-dependent local interactions and distance-independent nonlocal interactions (self-attention), combined with explicit electronic state encoding. The link atom has the richest interaction pathway of all five architectures:*

(a) Nuclear charge embedding. *The link atom enters as hydrogen ($Z = 1$):*

$$\boldsymbol{e}_{Z=1} = M \cdot d_H + \tilde{\boldsymbol{e}}_H \quad (13)$$

where $d_H = (1,0,\dots,0,1,0,0,0,1)^T$ encodes the $1s^1$ electron configuration, M is a learnable projection matrix, and $\tilde{\boldsymbol{e}}_H$ is a freely learnable bias.

(b) Electronic state embedding. *Through a softplus-weighted attention mechanism, the link atom receives a share of the total charge/spin:*

$$a_L = \frac{\Psi \cdot ln(1+exp(q_L^T k/\sqrt{F})r)}{\sum_j ln(1+exp(q_j^T k/\sqrt{F})r)} \quad (14)$$

where $\Psi = Q$ (charge) or S (spin). The attention weights satisfy $\sum_i a_i = \Psi$, exactly distributing the global electronic information across atoms. Since $q_L = linear(\boldsymbol{e}_{Z=1})$, the fraction of electronic embedding allocated to the link atom depends on the learned interaction between the hydrogen nuclear embedding and the charge/spin key vectors.

(c) Local interaction: s/p/d channel contributions. *The link atom contributes to the scalar, vector, and tensor channels of every QM atom within $rcut$:*

$$s_i += \backslash resmlp_s(\tilde{x}_L) \odot (G_s \cdot \rho_s(r_{iL})) \quad (15)$$

The p- and d-channel contributions encode angular information through spherical harmonics $Y_1^m(\hat{r}_{iL})$ and $Y_2^m(\hat{r}_{iL})$, providing directional sensitivity to the link atom position.

(d) Nonlocal interaction: global attention. *Uniquely among all architectures, SpookyNet's nonlocal module allows the link atom to interact with every atom in the ML region regardless of distance. The link atom's query/key/value vectors participate in global self-attention, meaning its influence is not bounded by $rcut$.*

EANN: Link Atom in GTO-Based Embedded Atom Density

EANN[10-12] *represents the atomic environment through a squared density built from Gaussian-type orbital (GTO) primitives.The link atom contributes to the embedded atom density (EAD) of every QM atom i within r_c:*

(a) Contribution to the EAD. *The link atom L (with orbital coefficient $c_L = c_H$) adds to the EAD:*

$$\rho_{i,n} \ni \cdots + \frac{l!}{l_x!\,l_y!\,l_z!}\left[\dots + cH \sum_m d_{mn}\,, x_{iL}^{l_x} y_{iL}^{l_y} z_{iL}^{l_z}\, e^{-\alpha_m (r_{iL}-r_m)^2} f_c(r_{iL}) + \cdots\right]^2 \quad (16)$$

where $l_x + l_y + l_z = l$ is the total angular momentum ($l = 0$: s-type, $l = 1$: p-type, $l = 2$: d-type), and α_m, r_m are the Gaussian width and center for the m-th primitive.

(b) Implicit three-body correlations with the link atom. *The squared-density construction*

generates cross-terms between the link atom and real QM neighbors:

$$\rho_{i,n} \ni \cdots + 2\, c_H\, c_j\ r_{iL}^l\, r_{ij}^l\ cos^l(\theta_{LiJ})\ \sum_{m,m'} d_{mn}\ d_{m'n}\, g_m(r_{iL})\, g_{m'}(r_{ij}) + \cdots \quad (17)$$

This encodes the angle θ_{LiJ} between the link atom direction and each QM neighbor direction, with increasing angular resolution for higher l. The angular dependence $cos^l(\theta)$ emerges naturally from the Cartesian GTO formulation without explicit angular symmetry functions.

Link atom's own density.

L has its own EAD ρ_L constructed from all QM neighbors within r_c. Its atomic energy $E_L = NN^H(\rho_L)$ contributes to the total.

Comparative Analysis and Universal Framework

Table S1 summarizes the qualitative differences in how the link atom participates across the five architectures. The diversity of descriptor extraction mechanisms underscores the non-trivial nature

of providing a universal link atom interface. Specifically, our framework must handle:

(1) ***Heterogeneous input tensor formats.*** *ANI expects species-indexed coordinate arrays; MACE requires edge index tensors and one-hot species vectors; AIMNet2 uses 2D embedding matrices; SpookyNet needs nuclear charge arrays plus global Q and S; EANN requires neighbor lists with Cartesian displacement vectors.*

(2) ***Architecture-specific validation.*** *The link atom species ($Z = 1$, hydrogen) must be in the model's training domain. For MACE-OFF23, this means $Z_L \in \{H, C, N, O, F, P, S, Cl, Br, I\}$; for ANI-1xnr, $Z_L \in \{C, H, N, O\}$. Our framework validates this at initialization.*

(3) ***Force computation consistency.*** *All backends compute forces via PyTorch autograd ($F = -\nabla E$ via energy.backward() or torch.autograd.grad), but differ in whether gradients are retained for higher-order derivatives, whether the computation graph is preserved, and how the gradient tensors are shaped and indexed. The link atom force redistribution Eqs.2-4 must receive consistent force arrays regardless of upstream computation details.*

(4) ***Unit conversion.*** *ANI and AIMNet2 output energies in Hartree ($\times\, 627.509 \rightarrow$ kcal/mol), while MACE, SpookyNet, and EANN output in eV ($\times\, 23.0605 \rightarrow$ kcal/mol). Forces undergo corresponding conversions.*

(5) ***Global electronic state management.*** *For AIMNet2 (NQE/NSE) and SpookyNet (electronic embedding), the total charge Q and spin multiplicity S of the ML subsystem must be correctly propagated through the Fortran--C++ interface, accounting for the link atom's effect on effective electron count.*

Table S1: Architecture-specific link atom participation. Each row represents a distinct computational pathway through which the link atom influences the MLIP's energy and force predictions.

Feature	ANI	MACE	AIMNet2	SpookyNet	EANN
**Descriptor type**	Symmetry functions (AEV)	ACE + message passing	2D embedding + message passing	s/p/d channels + attention	GTO density
**Link atom role**	Additive contribution to radial & angular sub-AEVs	Graph node in $E(3)$-equivariant convolutions	Neighbor in scalar/vector conv. + charge equilibration	Four-fold: nuclear, electronic, local, nonlocal	Orbital density source for GTO-based EAD
**Angular encoding**	Explicit θ_{ijk}	Spherical harmonics Y_l^m + CG coupling	Vector channel with $\hat{\mathbf{r}}_{ij}$	p/d channels with Y_1^m, Y_2^m	Implicit via $\cos^l(\theta)$ from squared GTO
**Body order**	3-body (pairwise + angular)	Up to $\frac{\nu^{T+1}-1}{\nu-1}$-body via iterated contraction	3-body (with iterative refinement)	Many-body via nonlocal attention	3-body via squared density
**Range of influence**	$R_c^{\text{ang}} \leq 3.5$ Å	$T \times r_{\text{cut}}$ (multi-hop)	$R_c = 5.0$ Å (+ Coulomb at 10 Å)	**Unbounded** (nonlocal attention)	$r_c \leq 6.0$ Å
**Species encoding**	Species-resolved sub-AEVs	One-hot → learnable embedding	2D embedding matrix per element	Electron configuration + bias	Orbital coefficient c_Z
**Electronic state**	None	None	NQE/NSE: Q, S	Charge + spin attention	None
**Energy unit**	Hartree	eV	Hartree	eV	eV

Question 3. Boundary treatment and embedding level versus claimed generality

The theoretical description in Section 2 closely parallels standard QM/MM formulations: the energy partitioning and link-atom force redistribution follow the usual pattern, and the electrostatic coupling is implemented at the mechanical embedding (ME) level, with fixed charges in the ML region and MM/ML interactions evaluated via classical Coulomb terms.

Mechanical embedding is certainly a reasonable first step and the results for MaDA are convincing. However:

- ME-level ML/MM applications have already been explored in several earlier works (just to name a few: 10.1021/acs.jctc.4c01427, 10.1021/acs.jcim.5c00033). The current state-of-art of the ME-level aims to reach the electrostatic embedding level (i.e. to take into account the effect of the environment in the ML region), both by post-hoc corrections (e.g.

10.1021/acs.jctc.4c00248, 10.1021/acs.jpcb.3c08166, 10.1021/acs.jctc.4c01792), or by directly including MM features into the ML model (eg. 10.1021/acs.jctc.5c00372, 10.1039/D4DD00295D).

- While ME seems to retrieve reasonable results for binding free energies (e.g., or even the previous work of the authors in ML/MM), recent studies (10.26434/chemrxiv-2025-nw9lt) have explicitly shown that ME reproduces electrostatic-embedding (EE) results only for fairly limited classes of reactions (e.g. when charge redistribution is modest, as in certain Diels-Alder reactions), and fails for more strongly polar or charge-transfer processes.

In this context, presenting an ME-level implementation as "redefining computational enzymology" seems too strong. To meet the novelty and generality expected for Nature Communications, I believe the authors should either:

- Demonstrate that their framework remains accurate for a broader range of enzymatic reactions, particularly those where EE or more advanced embeddings are known to be important; or

- Explicitly frame this work as a first step and significantly temper the claims about generality and "redefining" the field, clearly stating the current limitations of ME.

Response: We thank the reviewer for this critical and well-supported assessment. We agree that the scope of generality required clearer qualification, and we have revised the manuscript accordingly.

(1) Acknowledgement of ME-level embedding and its scope of validity. We acknowledge that our implementation adopts mechanical embedding following a formulation closely analogous to standard QM/MM practice, and that the distinction of our framework relative to prior ML/MM work lies primarily in its multi-backend compatibility and PLUMED integration, as discussed in our response to Reviewer 1, Question 2. Regarding the accuracy of ME for the specific system studied here, we note that Zinovjev et al. (ChemRxiv 2025) explicitly demonstrate that ME reproduces EE results for Diels-Alder cycloadditions, a reaction class characterised by modest charge redistribution, which provides direct posterior validation for our conclusions. Furthermore, in response to Reviewer 2, Question 2, we have performed a systematic ML region convergence test (see that response for full details). Because the MLIP itself does not carry explicit charges, the primary effect of ME-level approximation manifests in the ML-MM electrostatic coupling term. However, as electrostatic interactions decay as $1/r^2$, once the ML region is sufficiently large (in our case encompassing 12 catalysis-related residues that account for the majority of first-shell interactions identified in prior literature), the residual error introduced by approximate ML-MM coupling is expected to be negligible. This is directly confirmed by our convergence test: the computed barrier obtained with our production ML region is statistically indistinguishable from that obtained with a second-shell region comprising more than 50 residues. Taken together, we are confident that the ME approximation does not materially affect the conclusions of this

work for the Diels-Alderase system. Nevertheless, we have added an explicit discussion in the Conclusions sections clearly stating the assumptions underlying ME, identifying reaction classes for which this approximation may break down, and outlining the extension to electrostatic embedding as a natural next step.

(2) Use of universal potentials and implications for generalisability. We wish to draw attention to an important distinction between our framework and the work of Zinovjev et al. (ChemRxiv 2025): their implementation employs system-specific potentials trained on targeted datasets, whereas our framework is built around universal reactive potentials, specifically ANI-1xnr and AIMNet2-NSE, that require no system-specific training and are directly deployable within AMBER by any user. This design choice has direct implications for generalisability: because these potentials are trained across broad regions of chemical space, they carry an intrinsic capacity to describe a wide range of chemical reactions beyond the specific system studied here, without requiring the computational investment of bespoke potential development. We believe this represents a meaningful contribution to accessibility and practical generalisability, even within the current ME framework, and we have made this point explicit in the revised manuscript.

(3) Revised tone and scope of claims. We have removed "redefining computational enzymology" from the title and replaced broad generality claims throughout the introduction and conclusions with appropriately qualified language, scoping demonstrated accuracy to reactions with modest charge redistribution and presenting the current implementation explicitly as a well-validated first step toward more sophisticated embedding schemes.

The corresponding introduction are from Page 5, Line 74 to Line 82:
"*A central aspect of this implementation is the demonstration that a single link atom module can interface with MLIPs built on fundamentally different representation paradigms, including ANI, MACE, AIMNet2, SpookyNet and EANN, providing a unified and backend-agnostic codebase in which the active ML potential can be exchanged without modification to the boundary treatment or the broader simulation infrastructure. Crucially, the framework adopts state-of-the-art universal reactive potentials, in particular ANI-1xnr*[26]*and AIMNet2-NSE*[35]*, which are directly deployable within AMBER without any system-specific training, removing the barrier that has historically limited the accessibility of this framework.*"

The corresponding conclusion stating the advantages of universal potential with respect to chemical reaction generalizability is presented on Page 19, Line 325 to Line 344:
"To overcome these limitations, we developed a unified multiscale framework that integrates universal reactive MLIPs into AMBER with a link-atom boundary scheme, enabling accurate and efficient description of chemical reactivity in enzymatic systems without the need for system-specific potential training. Coupled with PLUMED-driven metadynamics, the framework achieves nanosecond-timescale simulations that directly identify transition states in the MaDA family and in aqueous solution, and is immediately accessible to experimental researchers. The adoption of universal potentials and native enhanced sampling compatibility position this work as

a foundation extensible beyond Diels-Alder chemistry to broader classes of enzymatic and condensed-phase reaction."

The corresponding conclusion softening the tone of our contributions and stating the limitations and future directions for electrostatic embedding is presented on Page 20, Line 354 to Line 362:

"We note that the current implementation adopts mechanical embedding, which performs well for the Diels-Alder system studied here owing to its modest charge redistribution; extension to electrostatic embedding remains an important next step toward broader applicability across more polar or charge-transfer reactions. By unifying chemical reactivity, environment effects, and statistical sampling in a single platform, this work represents one contribution toward rational enzyme design and next-generation AI-based and physics-informed computational enzymology."

Question 4. A significant part of the manuscript is devoted to the ML/MM framework itself (support for multiple MLIPs and integration into AMBER etc.), and the Conclusions highlight that the method achieves nanosecond-timescale simulations and is broadly applicable.

Given this emphasis, I think the manuscript would benefit from more quantitative performance benchmarks. For example:

- Wall-clock timings (or ns/day) for typical MaDA simulations on the reported hardware, compared to QM/MM or to pure MM.

- Scaling with ML region size and with different MLIPs (ANI-1xnr vs AIMNet-2, MACE, etc.), at least for small representative systems.

- Any overhead introduced by the link-atom force redistribution and electrostatic treatment relative to a ML/MM implementation without boundary handling.

Response: We thank the reviewer for this important suggestion. We have added two new tables (Tables S2 and S3) to the Supporting Information providing comprehensive performance benchmarks for our ML/MM framework.

(1) Scaling with ML region size and comparison to QM/MM. Table S3 reports simulation throughput (ps hour^{-1}) for both ANI-1xnr and AIMNet2-NSE across 14 test systems in which the ML region is expanded incrementally from 84 atoms (substrate only) to 822 atoms (full second coordination shell), while the total system size is held fixed at 75,607 atoms. QM/MM reference timings were obtained using the GROMACS--CP2K interface with the ω B97X/6-31G* functional and basis set. Across all benchmarked cases, ML/MM achieves speedups of approximately 690--955 times over QM/MM, with ANI-1xnr delivering 14-16 ps hour^{-1} and AIMNet2-NSE delivering 12-19 ps hour^{-1}. Crucially, ML/MM throughput is essentially independent of ML region size up to at least 222 atoms, confirming that the MLIP evaluation rather than the boundary treatment dominates the computational cost. The slight throughput reduction observed for the 822-atom second-shell system with AIMNet2-NSE is

consistent with the larger message-passing graph required by that architecture at increased region sizes.

(2) Overhead of the link-atom boundary treatment. To isolate the cost introduced by the QM/MM boundary handling, we compared Case 1 (84 ML atoms, substrate only, zero link atoms) with Case 12, which contains the same total number of ML atoms (84) but with six sidechain fragments and six link atoms (Table S2). The throughput of both cases is identical within numerical noise for both potentials (ANI-1xnr: 15.79 vs. 14.59 ps hour^{-1}; AIMNet2-NSE: 18.80 vs. 18.53 ps hour^{-1}), demonstrating that the link-atom force redistribution and charge redistribution scheme introduce negligible overhead relative to the MLIP evaluation itself.

(3) Reactive MLIP coverage. Among currently available reactive MLIPs, our framework supports ANI-1xnr and AIMNet2-NSE (used in the manuscript). We also attempted integration of two recent foundation models: (a) MACE-OMOL (trained on the OMol25 dataset): The model relies on *GenericJointEmbedding* with heterogeneous Python dict attributes, which cannot be compiled to TorchScript. Since our AMBER interface loads models via LibTorch, which requires TorchScript format, MACE-OMOL cannot be integrated at present. (b) UMA (Meta FAIRChem): Similarly built on the FAIRChem infrastructure with non-TorchScript-compatible components, precluding LibTorch loading. We therefore only benchmarked ANI-1xnr and AIMNet2-NSE as the two reactive MLIPs currently deployable within our framework.

The corresponding results illustrating the performance test are presented on Page 10, Line 166 to Line 168:

"*Systematic benchmarking againstωB97X/6-31G* QM/MM across ML regions spanning 84-822 atoms shows speedups of 690--955 times, with throughput independent of ML region size and negligible link-atom overhead (Tables S2-S3).*"

The corresponding computational methods showing how performance tests conducted are presented on Supporting Information Page S18 to Page S20:

"***QM/MM and ML/MM Performance Benchmarks***

To assess the computational efficiency of our ML/MM interface, we performed a systematic benchmarking study by comparing ML/MM and QM/MM simulation speeds across a series of test systems with progressively expanding QM/ML regions. All the simulations are performed on Intel(R) Xeon(R) w7-3455 with NVIDIA RTX 5070Ti.

***QM/MM setup**. QM/MM simulations were carried out using the GROMACS–CP2K interface obtained through joint compilation (GROMACS 2024.2 and CP2K 2024.2),[15,26] which automatically generates a CP2K input template from the system topology. The auto-generated template was modified only to replace the exchange–correlation functional and basis set with ωB97X/6-31G*; all other settings were retained. A time step of 0.5 fs was used and each simulation was run for 100 steps, with the per-step wall-clock time averaged over all steps to estimate throughput (ps hour−1). The spin multiplicity was set to 1 throughout; the total QM*

charge was adjusted for each system according to the net charge of the included residues (see Table S2). ML/MM setup. ML/MM simulations employed the interface developed in this work, using either ANI-1xnr or AIMNet2-NSE as the ML potential. The same 0.5 fs time step was applied. Each simulation was run for 10 000 steps and the average throughput was computed from the final 1 000 steps to ensure thermal equilibration of the timing measurements.

Test systems. *Fourteen test systems were constructed to span a wide range of ML/QM region sizes (Table S2). Cases 1–11 follow an incremental expansion protocol in which one additional protein residue sidechain is added to the ML region at each step, beginning from the substrate alone (Case 1, 84 atoms, no link atoms) and reaching the complete first-coordination-shell set of 11 fragments (Case 11, 222 atoms including 10 link atoms). Case 12 was designed to isolate the cost of the link-atom boundary treatment: it contains the same 78 sidechain atoms and 6 link atoms as would result from selecting those six sidechains without the substrate, yielding a region of identical total size (84 atoms) to Case 1 but with six QM/MM boundary bonds instead of none. Cases 13 and 142 correspond to the full first-shell (4 Å) and second-shell (8 Å) residue selections, comprising 19 and 58 residues respectively. Because QM/MM calculations become prohibitively expensive at these scales, QM/MM reference timings were not collected for Cases 13 and 14; ML/MM throughput values are reported for completeness.*”

Table S2: Composition of QM/ML Benchmark Systems

Case	QM/ML Region	ML atoms[a]	Link atoms	QM charge	Notes
1	Substrate	84	0	0	Substrate only (MOL); no boundary bonds
12	Leu + Thr + Ile_1 + Ile_2 + Arg + Leu_2	84	6	+1	Sidechains only; same total size as Case 1; used to quantify link-atom overhead
2	Substrate + Leu	98	1	0	
3	+ Thr_1	107	2	0	
4	+ Ile_1	121	3	0	
5	+ Ile_2	135	4	0	
6	+ Arg	154	5	+1	Arg sidechain carries charge +1
7	+ Leu_2	168	6	+1	
8	+ Phe	183	7	+1	
9	+ Thr_2	192	8	+1	
10	+ Tyr	208	9	+1	
11	+ Ile_3 (full inner shell)	222	10	+1	All first-coordination-sphere sidechains
13	First shell (4 Å, 19 residues)[b]	349	18	+1	QM/MM not computed[c]
14	Second shell (8 Å, 58 residues)[b]	822	59	+1	QM/MM not computed[c]

[a] ML atom count includes link atoms. [b] Shell residues were selected by identifying all non-Gly/Ala/Cys/Met protein sidechains with at least one heavy atom within 4 Å (Case 13) or 8 Å (Case 14) of any substrate atom; backbone atoms are excluded. [c] QM/MM reference simulations were not carried out for Cases 13 and 14 owing to the prohibitive computational cost of ωB97X/6-31G* calculations at these region sizes.

Table S3: ML/MM Simulation Throughput Benchmarked Against QM/MM

Case	ML atoms[a]	Link atoms	QM/MM (ps $hour^{-1}$)	ANI-1xnr		AIMNet2-NSE	
				ps $hour^{-1}$	Speedup[b]	ps $hour^{-1}$	Speedup[b]
12	84	6	0.019	14.59	751×	18.53	953×
1	84	0	0.021	15.79	745×	18.80	887×
2	98	1	0.021	15.83	746×	18.82	887×
3	107	2	0.021	16.15	754×	19.03	888×
4	121	3	0.021	16.11	755×	18.82	882×
5	135	4	0.021	15.91	743×	18.90	883×
6	154	5	0.021	14.75	693×	18.57	873×
7	168	6	0.021	14.72	698×	18.48	876×
8	183	7	0.021	14.56	690×	18.42	872×
9	192	8	0.021	14.67	698×	18.77	892×
10	208	9	0.021	14.68	712×	18.14	879×
11	222	10	0.020	14.59	732×	18.39	923×
13	349	18	—[c]	14.47	—[c]	17.59	—[c]
14	822	59	—[c]	14.50	—[c]	12.41	—[c]

[a] ML atom count includes link atoms. [b] Speedup = ML/MM throughput ÷ QM/MM throughput. [c] QM/MM reference data are unavailable for Cases 13 and 14 owing to the prohibitive computational cost of running ωB97X/6-31G* QM/MM simulations at these region sizes; speedup values are therefore not reported. The total simulation system comprises 75 607 atoms.

Question 5. The code repository includes a README file, but it does not provide any installation or setup instructions, which makes it difficult to use as a standalone resource.

Response: We thank the reviewer for this practical suggestion. We have updated the MLMM4AMBER repository (https://github.com/ClickFF/MLMM4AMBER) to include:

1. A comprehensive `INSTALL.md` with step-by-step instructions for:
- Prerequisites (AMBER22/23, LibTorch, CUDA toolkit)
- Compilation from source with CMake
2. An example workflow for setting up an ML/MM MD simulation from scratch

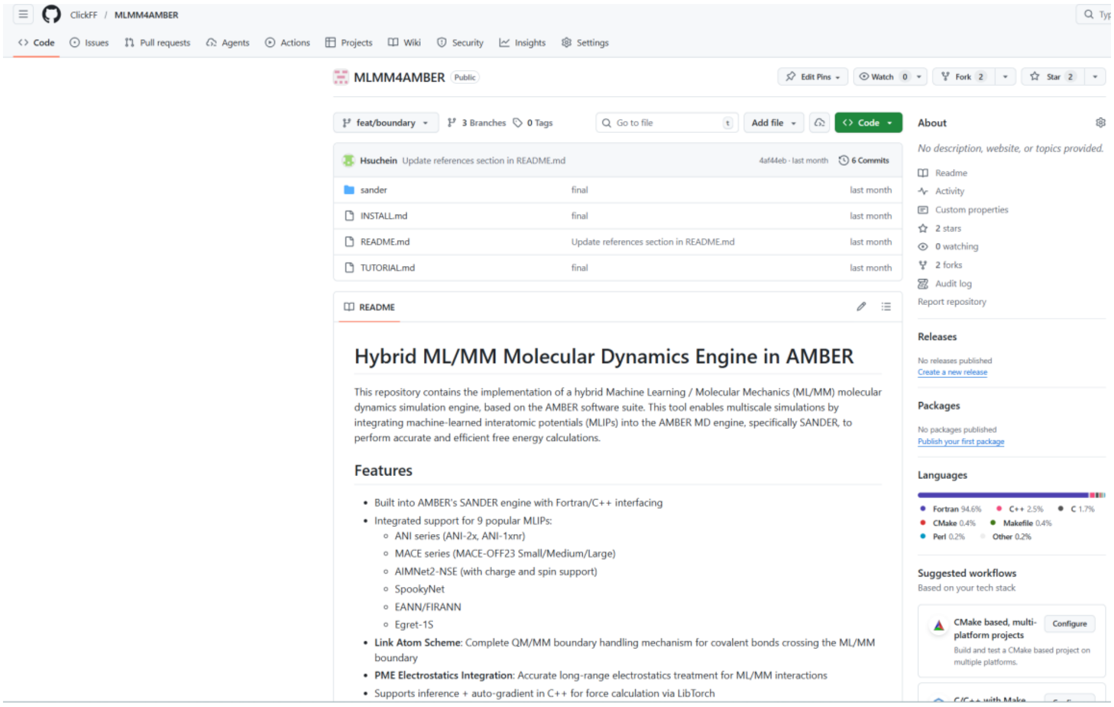

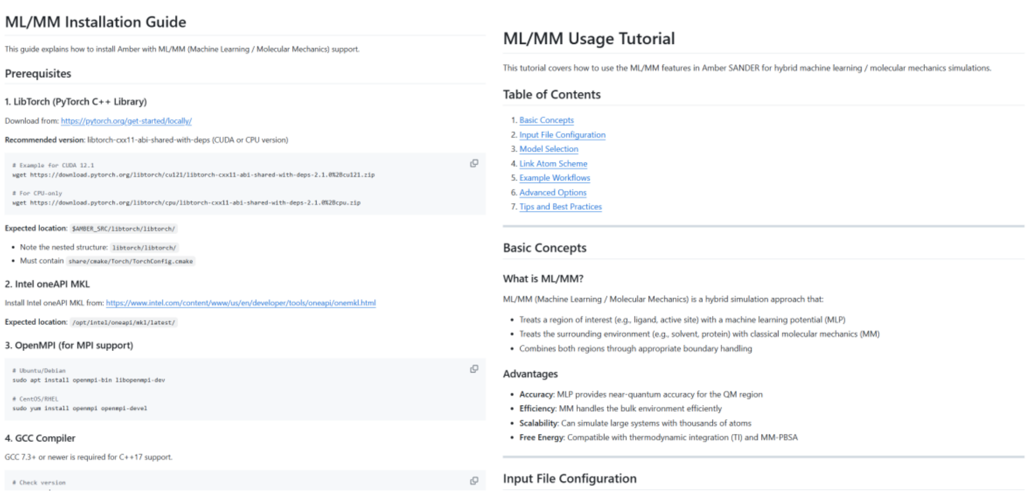

Response to Reviewer 2:

Recommendation: This work explores enzyme catalysis with a newly implemented ML/MM approach. The link method is taken from the QM/MM community and applied to ML/MM simulations. The link method solves the problem of dangling bonds between the MM and ML regions by adding an additional atom to stabilize bonds. The study then applies this new implementation to Diels–Alder reactions and studies the resulting mechanisms observed. DFT and experimental values are compared to the ML/MM results.

The introduction of this new method to ML/MM computations is a useful addition to the community and this is an important problem. However, the subsequent evaluation of the method is limited in detail. There is no benchmarking of the performance of the link method itself. For example, there is no evaluation of how moving to ML/MM from an entirely ML description changes performance. Additionally, the authors do not investigate how the size of the ML region influences the results or whether the results converge with increasing ML region size. It is not clear by the end of the work how well the link method really performs or what additional error it has introduced.

Furthermore, the use of ANI-1xnr to study this system in such detail is not a good choice. ANI-1xnr is trained to periodic condensed phase systems. In this work, there are small cluster of molecules and given that ANI-1xnr does not work well in the gas phase the use of this potential needs to be justified. This should be carried out through careful evaluation of the potential in comparison to QM results for this system. Unfortunately, I expect it will be found to be highly inaccurate. Furthermore ANI-1xnr is trained on low-fidelity QM calculations which will further reduce accuracy of this potential. Given this, it is highly likely the ANI-1xnr potential is inaccurate for this system and therefore the careful evaluation of the mechanisms explored are not particularly useful.

Given these two major points, I cannot recommend publishing the paper in its current form.

Question 1. Comparing ML to ML/MM on small systems to evaluate the link method and ML/MM as a model.

Response: We thank the reviewer for this important request. To more rigorously evaluate the numerical error introduced by the link-atom boundary treatment, we have performed a series of NVE molecular dynamics benchmarks on a tyrosine--tyrosine (Tyr--Tyr) dipeptide in vacuum (45 atoms total), comparing four simulation protocols: (1) classical force field at Δt=1.0 fs, (2) classical force field at Δt=0.1 fs, (3) pure MLIP (ANI-1xnr) at Δt=0.1 fs, and (4) ML/MM with link atoms at Δt=0.1 fs. In the ML/MM simulation, the two tyrosine sidechains (30 atoms, from C_β onward) were assigned to the ML region, with link atoms placed at each C_α-C_β boundary, directly replicating the boundary topology used in the enzymatic production simulations. All simulations were run in the microcanonical (NVE) ensemble with no thermostat, no periodic boundaries, and an effectively infinite nonbonded cutoff, to isolate any energy drift as a direct measure of numerical error from each method.

The results are presented in the new Fig. S2 and demonstrate three key findings. First, at the same timestep of 0.1 fs, the energy conservation of the ML/MM simulation (SD = 0.0029 kcal mol^{-1}, drift = 0.0084 kcal mol^{-1} over 0.5 ns) is indistinguishable from that of the pure MLIP simulation (SD = 0.0050 kcal mol^{-1}, drift = -0.0027 kcal mol^{-1}) and from the classical force field at the same time step (SD = 0.0006 kcal mol), drift = -0.0002 kcal mol^{-1}). This directly demonstrates that the link-atom Jacobian force redistribution introduces no measurable additional numerical error into the total Hamiltonian beyond that already present in the underlying MLIP evaluation. Second, a comparison of the classical forcefield at 1.0 fs versus 0.1 fs (SD = 0.0528 vs. 0.0006 kcal mol^{-1}) shows that the MD integration timestep itself is by far the dominant source of energy drift, exceeding the link-atom contribution by more than an order of magnitude. Third, the overlay of energy drift across all four protocols (Fig. S2) confirms that the link-atom ML/MM curve is entirely contained within the envelope of the pure MLIP simulation, with no systematic drift attributable to the boundary treatment over 0.5 ns.

From a theoretical standpoint, this outcome is expected: the link-atom forces are redistributed to the physical boundary atoms through an exact chain-rule Jacobian projection (Eqs.~2--7 in the main text), which ensures that the total energy gradient is computed consistently and that no spurious forces accumulate over time. The conservation test provides practical confirmation that this theoretical guarantee is correctly implemented. We have added this analysis as a new subsection of the Supporting Information.

The corresponding computational methods of energy conservation tests are presented on Supporting Information Page S16 to Page S17:

*"**Link Atom Energy Conservation Validation***

To assess the numerical error potentially introduced by the link atom scheme at the ML/MM boundary, we performed a series of NVE molecular dynamics benchmarks on a tyrosine－tyrosine (Tyr－Tyr) dipeptide in vacuum (45 atoms). The system was constructed using tLEaP with the ff14SB force field[26] and energy-minimized (5000 cycles) prior to dynamics. An initial NVE equilibration was carried out at 300 K with Δt = 1.0 fs for 1 ns using the classical force field. Four production NVE simulations were then restarted from the equilibrated coordinates and velocities (Figure S2). All runs used ntt = 0 (microcanonical ensemble), no periodic boundaries, and an effectively infinite nonbonded cutoff (9999 Å). The ANI1-xnr potential was employed.[4] In the ML/MM simulation, only the sidechain atoms from Cβ onward (atoms @7－21 and @28－42, 30 atoms total) were assigned to the ML region, with link atoms placed at each Cα－Cβ boundary. The energy conservation of the ML/MM simulation (ΔE = 0.009 kcal/mol over 0.5 ns, Figure S2) confirms that the link atom treatment at the ML/MM boundary introduces negligible numerical error into the Hamiltonian, comparable to the intrinsic integration noise of the ML potential itself."

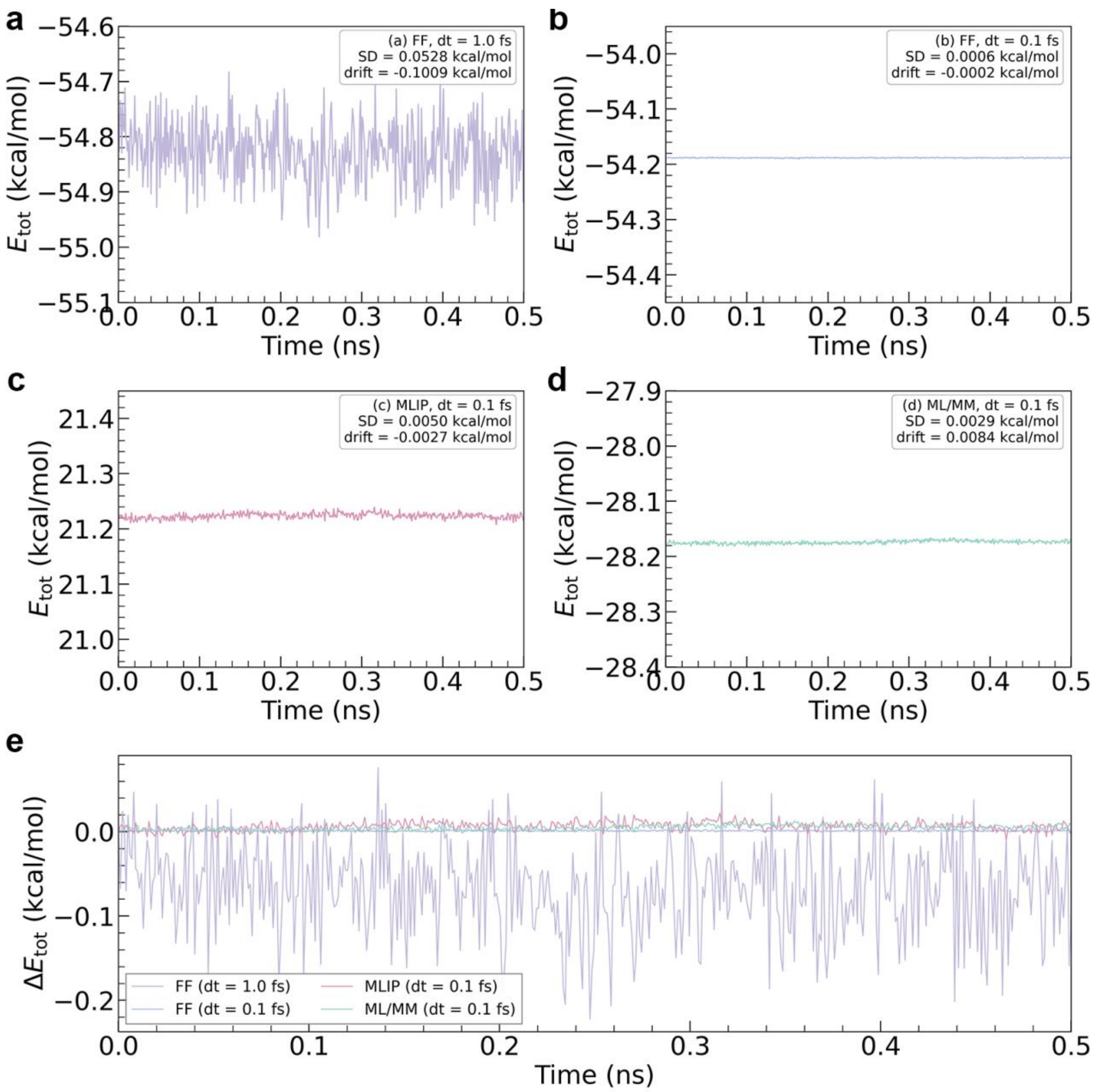

Figure S2: Energy conservation benchmarks for NVE simulations of the Tyr - Tyr dipeptide in vacuum. (a) - (d) Total energy Etot(t) for each simulation over 0.5 ns, with the method, timestep, standard deviation (SD), and total energy drift annotated in each panel. All panels share a 0.5 kcal/mol y-axis range to facilitate visual comparison. (e) Overlay of the total energy drift ΔEtot(t) = Etot(t) − Etot(0) for all four simulations.

Question 2. Comparing varying size regions of ML in the ML/MM simulations to show convergence

Response: We thank the reviewer for this important suggestion. To demonstrate convergence with respect to ML region size, we have performed additional ML/MM MetaD simulations of MaDA-3 (endo pathway) using four systematically expanded ML region definitions: (1) substrate only (84 atoms, no link atoms); (2) substrate plus 12 catalysis-related residues (222 atoms, 12 link atoms), which corresponds to the region used throughout the main text; (3) a first-shell selection comprising all non-Gly/Ala/Cys/Met sidechains with at least one heavy atom within 4 Å of the substrate (362 atoms, 19 link atoms, 19 residues); and (4) a

second-shell selection extending this cutoff to 8 Å (833 atoms, 58 link atoms, 58 residues). The results, summarized in the new Figure S3 and reproduced from five independent simulations per region size, show that the computed activation barrier converges rapidly upon inclusion of the catalysis-related residues: the substrate-only region yields 34.1±4.4 kcal mol^{-1}, while the catalysis-related, first-shell, and second-shell regions give 30.0±1.7, 29.4±1.8, and 30.0±1.8 kcal mol^{-1}, respectively. The barrier is thus well-converged beyond the catalysis-related region, and further expansion to the second shell introduces no statistically significant change. These results confirm that the ML region employed in the main text is sufficient for quantitative accuracy and that the conclusions are robust to the choice of region boundary.

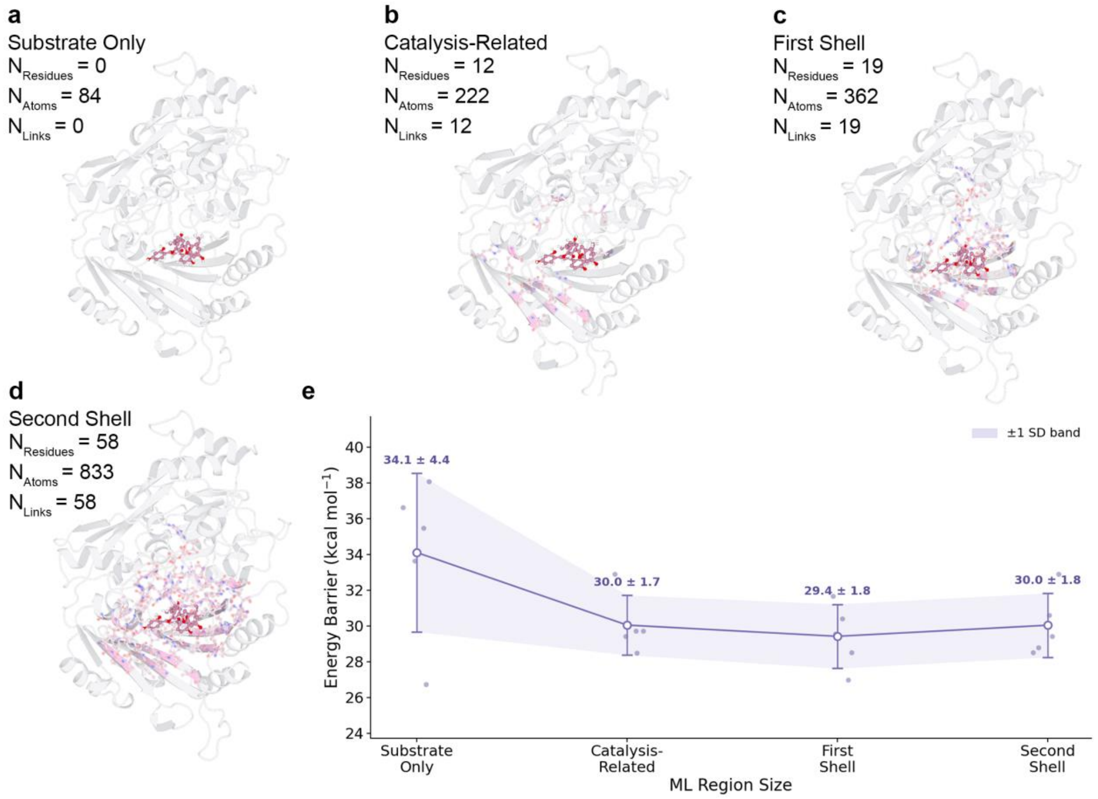

Figure S3: Convergence of the ML/MM activation barrier with respect to ML region size. a-d, Representative structures illustrating the four ML region definitions used in the convergence study. ML atoms are shown as sticks coloured by element; the protein backbone is rendered as a grey cartoon. N_{Residues}, N_{Atoms}, and N_{Links} denote the number of residue sidechains, total ML atoms (including link atoms), and QM/MM boundary link atoms, respectively. **e,** Computed endo activation free energy barrier ($\Delta G^{\ddagger}, kcal\,mol^{-1}$) for MaDA-3 as a function of ML region size. Each data point represents an independent ML/MM MetaD simulation (small circles); the large circle and error bars indicate the mean ± one standard deviation across replicates; the shaded band shows the ±1 SD envelope across all four region sizes.

The barrier converges upon inclusion of the catalysis-related residues and remains stable through first- and second-shell expansions, confirming that the region used in the main text (Catalysis-Related) is sufficient for quantitative accuracy.

The corresponding results showing the convergence tests are presented on Page 10, Line 175 to Line 181:

"*To determine the minimal ML region sufficient for converged barriers, we performed MetaD simulations of MaDA-3 with four progressively expanded ML regions, from substrate only (84 atoms) to a second coordination shell (833 atoms). The activation barrier converges upon inclusion of the catalysis-related residues identified in prior work (222 atoms, 12 residues; 30.0 ± 1.7 kcal mol⁻1), with no statistically significant change upon further expansion (Fig. S3), and this region was therefore adopted for all production simulations.*"

The corresponding computational methods showing how convergence tests conducted are presented on Supporting Information Page S21 to Page S22:

"***ML Region Convergence Test***

To verify that the computed activation free energy barriers are insensitive to the choice of ML region boundary, we performed a systematic convergence study using MaDA-3 as the representative system. Four ML region definitions of increasing size were constructed:

• Substrate only. The ML region comprised the two substrate molecules (84 atoms, 0 link atoms). No protein residues were included and no ML/MM boundary bonds were introduced.

• Catalysis-related (main-text region). The 12 protein sidechain residues most directly involved in catalysis were added to the substrate, yielding 222 ML atoms and 12 link atoms. This is the region employed in all ML/MM MetaD simulations reported in the main text.

• First shell. All non-Gly/Ala/Cys/Met protein sidechains possessing at least one heavy atom within 4 Å of any substrate heavy atom were included (19 residues, 362 ML atoms, 19 link atoms). Backbone atoms were excluded throughout.286

• Second shell. The distance threshold was extended to 8 Å, giving 58 residues, 833 ML atoms, and 58 link atoms.

For each region definition, five independent ML/MM MetaD simulations were carried out using the ANI-1xnr potential, following the same protocol as described in the main Methods section (collective variables, hill height and width, temperature, and time step identical to the production runs). The endo activation free energy barrier $\Delta G\ddagger_{endo}$ was extracted from each simulation, and the mean and standard deviation were computed across the five replicates. Protein residues outside the ML region were treated at the MM level using the AMBER ff14SB force field, with ML/MM boundary bonds capped by hydrogen link atoms placed along the Cα–Cβ bond of each boundary residue. The total charge of the ML region was adjusted for each system according to the net formal charge of the included residues."

Question 3. Validating the ANI-1xnr potential for the chosen system through QM calculations and ideally calculations of the barrier height for the reaction in the cluster

Response: We thank the reviewer for this critical point. Rigorous validation of the MLIP for the specific chemistry under study is indeed essential. To address this concern, we have performed comprehensive potential energy surface (PES) benchmarks that go beyond barrier comparisons and assess the fidelity of ANI-1xnr across the entire reactive landscape relevant to the Diels-Alder cycloaddition:

(1) Two-dimensional PES comparison across the full reactive surface. We constructed two-dimensional relaxed PES scans for both the endo and exo Diels-Alder pathways of the substrate in the gas phase. The two forming C-C bond distances (denoted AB and CD) were scanned from 1.5 to 4.0 Å in increments of 0.05 Å, yielding a grid of 2,601 structures per pathway (5,202 structures in total). At each grid point, the geometry was optimized at the ωB97X/6-31G* level using Gaussian, with the two scanned distances frozen. The resulting DFT energies define a reference PES against which the MLIP can be evaluated. For each of the 5,202 optimized structures, single-point energies were then evaluated with ANI-1xnr. The results are presented in the new Figure S8 of the Supporting Information: the left column shows the DFT reference PES, the center column shows the ANI-1xnr PES, and the right column shows the unsigned error |DFT − MLP|. Key observations include: (i) The overall topography of the PES, including the positions of the reactant basin, transition-state saddle region, and product well, is faithfully reproduced by ANI-1xnr for both the endo and exo pathways. (ii) The root-mean-square errors over the full 2,601-point grid are 4.44 kcal/mol for the endo pathway and 4.39 kcal/mol for the exo pathway. Although these errors are not negligible in absolute terms, they are distributed relatively uniformly across the surface rather than being concentrated near the transition state, indicating that ANI-1xnr captures the essential shape of the PES without systematic distortion of the reaction channel. (iii) Using a minimum-energy path (MEP) search algorithm on the ANI-1xnr PES, the resulting reaction path is in close agreement with the MEP obtained from the DFT surface, confirming that the MLIP does not shift or distort the intrinsic reaction coordinate. This analysis provides substantially stronger validation than a single-point barrier comparison, because it demonstrates that the MLIP is reliable over the entire region of configuration space sampled during metadynamics, not merely at stationary points.

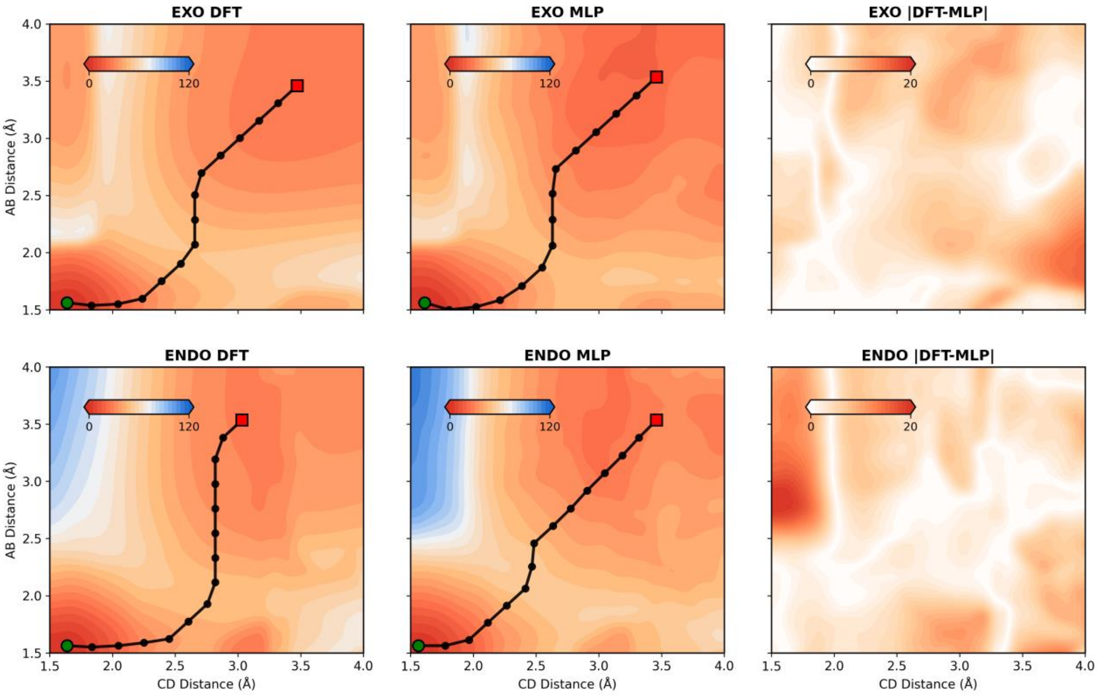

Figure S1: Comparison of two-dimensional potential energy surfaces for the *exo* (top row) and *endo* (bottom row) Diels-Alder pathways. Left: DFT reference (ωB97X/6-31G*). Centre: ANI-1xnr single-point energies. Right: unsigned error $|E_{\mathrm{DFT}} - E_{\mathrm{MLP}}|$. The energy colour scale is in kcal mol^{-1}. Black curves with dots denote the minimum-energy paths obtained by the Dijkstra algorithm. Green circles mark the reactant minimum; red squares mark the product minimum. RMSEs over the full grid are 4.39 kcal mol^{-1} (*exo*) and 4.44 kcal mol^{-1} *endo*.

(2) Broader implications for the framework. The rapidly improving landscape of reactive universal MLIPs is an encouraging development for the present framework. We have added an explicit statement to the Conclusion acknowledging that ANI-1xnr, while well-suited to the Diels-Alder chemistry studied here, represents a first step with limited extensibility to more chemically diverse reaction classes. More capable next-generation models, including UMA, AIMNet2-NSE, and AIMNet2-rxn, should be incorporated into the framework in future work to broaden its applicability.

(3) AIMNet2-NSE benchmarking on the six core systems. Having since integrated AIMNet2-NSE into the framework, we performed ML/MM MetaD calculations for all six primary systems studied in the main text. Compared with ANI-1xnr, AIMNet2-NSE yields activation barriers 2-3 kcal mol^{-1} closer to the DFT reference values (Table S5), consistent with its more advanced architecture and charge-equilibration scheme. These results are included in the revised manuscript and indicate that future applications should preferentially employ newer-generation reactive universal potentials as they become available.

Table S5: Activation free energies ($\Delta G^{\ddagger}$, kcal mol^{-1}) of MaDA1 and MaDA3 along the *endo*/*exo* pathways, as obtained from experiment, DFT cluster models, and ML/MM metadynamics. The symbol / denotes cases where no previous experimental or computational data were available. DFT QM cluster model data from Refs.[27,28] All energies are reported in kcal mol^{-1}.

	MaDA3 *endo*	MaDA3 *exo*	MaDA1 *endo*	MaDA1 *exo*	Aqueous *endo*	Aqueous *exo*
Experimental	/	19.29	16.14	/	/	/
DFT cluster	/	20.8	20.3	/	23.3	26.1
ML/MM MetaD (ANI-1xnr)	30.04	25.75	25.08	29.33	27.68	30.31
ML/MM MetaD (AIMNet2-NSE)	22.72	23.13	23.72	27.01	23.76	27.91

(4) Limitations of ANI-1xnr and the landscape of reactive universal MLIPs. We appreciate the reviewer's perspective and wish to provide important context regarding our choice of ANI-1xnr. This project was initiated in early 2025, at which point ANI-1xnr was one of the very few reactive universal MLIPs available that were both broadly applicable and state-of-the-art. The next-generation models the reviewer may have in mind appeared considerably later: AIMNet2-rxn was posted to ChemRxiv in June 2025, UMA (Meta FAIR) appeared on arXiv in June 2025, MACE-OMOL was released in August 2025, and AIMNet2-NSE was published in Angewandte Chemie in January 2026. All of these models were therefore unavailable at the time our simulations were designed and initiated, and our selection of ANI-1xnr reflects the state of the field at that moment rather than an oversight. We hope this timeline clarifies the rationale behind our choice.

The corresponding discussion illustrating the robustness of the ANI-1xnr for current systems are presented on Page 9, Line 163 to Page 10, Line 166:
"Two-dimensional PES scans along the endo and exo forming-bond coordinates at the DFT and ANI-1xnr levels confirm close agreement in reaction pathway geometry and energetics, with only minor deviations in non-reactive regions (Fig. S7)."

The corresponding computational methods of the potential energy surface scans are presented on Supporting Information Page S13 to Page S15:
*"**Gas-Phase Potential Energy Surface Validation of ANI-1xnr***
*To assess the fidelity of the ANI-1xnr potential across the entire reactive landscape, we constructed two-dimensional potential energy surfaces (PES) for both the endo and exo Diels–Alder pathways and compared the ANI-1xnr predictions against DFT reference calculations. PES Construction For each stereochemical pathway (endo and exo), the two forming C–C bond distances (denoted AB and CD) were scanned from 1.5 to 4.0 Å in increments of 0.05 Å, yielding a uniform grid of 51 × 51 = 2,601 structures per pathway (5,202 structures in total). At each grid point, a constrained geometry optimisation was performed using Gaussian 16 at the ωB97X/6-31G*23,24 level of theory: the two scanned bond distances were frozen while all remaining internal coordinates were fully relaxed. The resulting DFT energies define the reference PES. For each of the 5,202 optimised structures, single-point energies were then evaluated with ANI-1xnr using MAPLE25 package. MAPLE provides a unified inference interface for multiple*

MLIP backends including ANI-family models, enabling batch evaluation of energies and forces on arbitrary molecular geometries.

Minimum-Energy Path Search

To further assess whether the MLIP preserves the intrinsic reaction coordinate, we performed minimum-energy path (MEP) searches on both the DFT and ANI-1xnr surfaces using a modified Dijkstra algorithm with a path-length penalty.

Algorithm. *The two-dimensional PES is represented as a regular grid graph in which each node* (i, j) *corresponds to a grid point with energy* E_{ij}*, and edges connect each node to its eight nearest neighbours (including diagonals). For a diagonal step the edge length is* $\sqrt{2}\,\Delta$*, whereas for an axial step it is* Δ*, where* $\Delta = 0.05$ Å *is the grid spacing.*

To find the path of lowest energy barrier that also avoids unnecessarily circuitous routes, we define a composite cost function. For a path $\mathcal{P} = \{(i_0, j_0), (i_1, j_1), \ldots, (i_N, j_N)\}$ *from the reactant node to the product node, the cost is:*

$$C(\mathcal{P}) = \max_{k\in\mathcal{P}} E_{i_k j_k} + \lambda \sum_{k=1}^{N} d_k$$

where $\max_{k} E_{i_k j_k}$ *is the highest energy encountered along the path (the bottleneck energy),* d_k *is the Euclidean step length between consecutive nodes, and λ is a path-length penalty coefficient (in kcal* □□□$^{-1}$ Å^{-1}*) that penalises unnecessarily long detours. This formulation seeks the path whose maximum energy is lowest, with the penalty term breaking ties in favour of shorter, more direct routes. A value of* $\lambda = 1.0$ *was used throughout.*

This cost function is minimised globally using Dijkstra's algorithm adapted for the composite objective: at each step, the algorithm expands the unvisited node with the smallest accumulated cost

$$C_{acc}(i_k, j_k) = max\backslash bigl\,(C_{acc}(i_{k-1}, j_{k-1}), E_{i_k j_k}\backslash bigr) + \lambda\, d_k$$

initialised with $C_{acc}(i_0, j_0) = E_{i_0 j_0}$*. Because the cost is monotonically non-decreasing along any extension of the path, the algorithm is guaranteed to find the global optimum.*

Post-processing. *The raw grid path was smoothed using a cubic B-spline interpolation with 500 intermediate points, followed by uniform arc-length resampling to* N_{pts} *evenly spaced points along the path. Energies at the resampled coordinates were obtained by bilinear interpolation on the original grid."*

The corresponding discussions stating more advanced potential (AIMNet2-NSE) is more accurate than ANI1-xnr are presented on Page 10, Line 184 to Page 12, Line 192:

"Encouragingly, ML/MM MetaD simulations performed with AIMNet2-NSE yielded barriers substantially closer to the DFT reference, reducing the mean unsigned deviation to approximately 2–3 kcal mol−1 across all six pathways (Table S5), suggesting that more advanced reactive

universal potentials hold considerable promise for further improving quantitative."

The corresponding discussion of limitations in Conclusion section is presented on Page 19, Line 354 to Line 358:
"While ANI-1xnr serves as a capable and practical starting point for closed-shell pericyclic chemistry, more accurate and transferable next-generation reactive potentials, such as AIMNet2-rxn[27], MACE-OMOL[52], and UMA[53], are expected to further improve accuracy and extend the scope of the framework to a wider range of reaction classes as they mature and become integrated"

Question 4. As an additional minor comment, much of the text is well written but the introduction is lacking a careful description of the problem they are trying to solve (dangling bonds between regions) and also does not fully describe the link method (which is the major methodological contribution). Additionally, the ANI-1xnr is not described in the methods section - both the model architecture and the training data used should be carefully outlined. As well as this, a literature review of other ML/MM methods, for example: https://pubs.acs.org/doi/abs/10.1021/acs.jctc.5c01464 should also be provided.

Response: We thank the reviewer for these constructive suggestions. We have made the following revisions:

(1) Expanded Introduction: We have expanded the introduction to explain why severing a covalent bond at the ML/MM boundary creates an unsaturated valence that corrupts the local chemical environment representation of the MLIP. We then describe the link-atom method and articulate its practical advantages: the link atom is regenerated deterministically at every MD step from the positions of the two boundary atoms, introducing no additional degrees of freedom; it saturates the dangling valence without basis-set superposition or charge-transfer artifacts; and the associated forces are redistributed to the physical atoms through an exact chain-rule Jacobian projection (Eqs. 3-4), which preserves energy conservation in microcanonical trajectories. We also note that these properties make the link-atom scheme particularly well suited to MLIP backends, where the descriptor space must receive a chemically complete local environment. The Theory section (Section 2.1) retains the full mathematical derivation, from link-atom positioning (Eq. 1) through the Jacobian-based force redistribution (Eqs. 2-7), with explicit discussion of what is standard QM/MM formalism versus what is specific to the MLIP context.

(2) ANI-1xnr description in SI: A new section in the Supporting Information ("Reactive Machine Learning Interatomic Potentials Used in This Work") now provides a detailed description of both ANI-1xnr and AIMNet2-NSE. For ANI-1xnr, we describe the model architecture (modified Behler–Parrinello symmetry functions with 5.2 Å radial and 3.5 Å angular cutoffs, element-specific feed-forward neural networks), the training data

generation procedure (active learning with oscillating temperature/density, BLYP/TZV2P-D3 reference calculations via CP2K, 26,650 periodic simulation cells spanning >1,200 unique molecular species). For AIMNet2-NSE, we describe the invariant message-passing architecture with Neural Spin Equilibration (NSE), which enables the model to handle open-shell systems and charged species by distributing the global charge Q and spin multiplicity S across atoms through a learned Fukui-like mechanism.

(3) Literature review of ML/MM methods: We have added a comprehensive survey of ML/MM developments to the Introduction, covering: (i) embedding schemes, from the mechanical embedding used in AMBER-based ML/MM work to the electrostatic embedding of Zinovjev et al. (emle-engine, JCTC 2024) and the polarisation-corrected mechanical embedding of Semelak et al. (JCTC 2025); (ii) ML/MM applications to enzymatic reactions, including the substrate-only reactive ML/MM studies by Zinovjev et al. (ChemRxiv 2025) and Lei et al. (JCTC 2025); (iii) recent tools such as the ML/MM Toolkit of Ohmura et al. (JCTC 2025, https://pubs.acs.org/doi/abs/10.1021/acs.jctc.5c01464), which the reviewer specifically recommended and which we now cite; and (iv) three recent review articles providing comprehensive accounts of these developments (Grassano et al., Chem. Phys. Rev. 2025; Andreichev et al., arXiv 2025; Świderek et al., WIREs Comput. Mol. Sci. 2025). This review contextualizes our contribution within the rapidly growing ML/MM landscape and highlights the specific gap, which is operational link-atom support for incorporating active-site residues into the MLIP region that our work addresses.

The corresponding comprehensive literature review on ML/MM development across different software packages and embedding schemes has been added to the Introduction from Page 4, Line 42 to Line 55:

"*Building on this, the ML/MM framework replaces the QM region with MLIPs, achieving both efficiency and accuracy. In recent years,ML/MM has been implemented across several mainstream MD software platforms, including GROMOS*[15,16]*, GENESIS*[17]*, and AMBER*[18-21]*. A central challenge common to all ML/MM implementations is the treatment of the coupling between the ML and MM subsystems. The most simplest approach, mechanical embedding (ME), evaluates the ML region in vacuo and adds classical non-bonded interactions between subsystems; this strategy has been employed in early AMBER-based ML/MM work*[18,20]*. However, ME ignores the polarisation of the ML region by the MM environment. Therefore, a more advanced polarisation-corrected mechanical embedding (PCME) schemes have been developed by Semelak et al.*[21]*; subsequently, Zinovjev et al.*[19,22] *introduced an electrostatic embedding. Beyond the embedding scheme, progress in ML/MM spans long-range electrostatic treatments, model architectures, and coupling strategies; comprehensive accounts of these developments can be found in recent reviews*[23–25]*.*"

The corresponding description of the link atom scheme has been added to the Introduction from Page 5, Line 63 to Line 70:

"To overcome this limitation, we draw on decades of QM/MM experience with boundary schemes including hybrid orbitals[29]*, buffer methods*[30]*, and pseudobonds*[31]*, and adopt the link-atom method*[32] *(Fig. 1c), the most widely used approach*[33]*, to incorporate active-site residues into the*

ML region and thereby extend reactive ML/MM to the full breadth of enzymatic mechanisms. In the link-atom scheme, a hydrogen capping atom is inserted along each severed covalent bond at every MD step, saturating the dangling valence of the boundary atom and providing the MLIP descriptor with a chemically complete local environment."

The corresponding introduction of the architecture and training dataset ANI1xnr used has been added to the Supporting Information from Page S10 to Page S12:

"Reactive Machine Learning Interatomic Potentials Used in This Work

ANI-1xnr

ANI-1xnr[4] is a general-purpose reactive MLIP developed by Zhang et al. for condensed-phase chemistry of the elements C, H, N, and O. The model builds on the ANI architecture[2,3,13], which decomposes the total energy into atomic contributions via species-specific neural networks operating on Atomic Environment Vectors (AEVs), a modified Behler--Parrinello symmetry function representation.

Architecture. *ANI-1xnr uses radial ($R_c^{\mathrm{rad}} = 5.2$ Å) and angular ($R_c^{\mathrm{ang}} = 3.5$ Å) cutoffs to construct the AEV descriptor for each atom. The AEV consists of species-resolved radial and angular sub-vectors, and a separate feed-forward neural network is trained for each element (C, H, N, O). The total energy is:*

$$E = \sum_i \mathrm{NN}^{Z_i}\left(\mathbf{G}_i^{Z_i}\right)$$

where $\mathbf{G}_i^{Z_i}$ is the AEV of atom i with element Z_i. Similar to previous ANI models, ANI-1xnr predicts energies based solely upon atomic positions and element types; it does not explicitly depend on the total charge or spin multiplicity. Forces are obtained via automatic differentiation. In our ML/MM framework, the model is loaded through a C++ interface linked via LibTorch (the C++ frontend of PyTorch[14]).

Training data. *Unlike prior ANI models that were trained on near-equilibrium organic molecules in vacuo, ANI-1xnr was developed through an automated nanoreactor active learning (NR-AL) workflow designed to sample condensed-phase reactive chemistry.[4] The NR-AL procedure initializes periodic simulation cells with randomly placed small molecules (e.g. C_2H_2, NH_3, H_2O, CO, CH_4) and drives MLIP-accelerated molecular dynamics under oscillating temperature (up to ~6000 K) and density conditions to promote chemical reactions. High-uncertainty structures, identified by ensemble disagreement, are selected and labelled with single-point DFT calculations performed using CP2K[15] at the BLYP[16,17]/TZV2P[18] level of theory with Goedecker--Teter--Hutter (GTH) pseudopotentials[19] and Grimme D3 dispersion correction with zero damping.[20] All reference calculations used unrestricted Kohn--Sham DFT with singlet spin multiplicity for the periodic simulation cell. After more than 50 AL iterations, the resulting training set comprises 26,650 periodic simulation cells with an average system size of 139 atoms, spanning densities from $\sim 0.03 to > 2.0\ g\ \mathrm{cm}^{-3}$ and containing over 1,200 unique molecular species identified by cross-referencing with the PubChem database.*

AIMNet2-NSE

AIMNet2-NSE[7,8] *is an invariant message-passing neural network potential with Neural Charge/Spin Equilibration, developed for open-shell and charged reactive chemistry. It extends the AIMNet2 architecture*[7] *with explicit handling of both total charge* Q *and spin multiplicity* S.

Architecture. *AIMNet2-NSE uses a 2D learnable atomic embedding and T iterations of message-passing convolution with scalar (*$l = 0$*) and vector (*$l = 1$*) channels, using Gaussian radial basis functions (*$S = 16$ *radial shells) with a cutoff of* $R_c = 5.0$ Å.[7] *The total energy is decomposed into local, dispersion (D4), and Coulomb contributions:*

$$U_{\text{Total}} = U_{\text{Local}} + U_{\text{Disp}} + U_{\text{Coul}}$$

The critical distinguishing feature is the Neural Spin Equilibration (NSE) module,\cite{aimnetnse} which constrains the spin-resolved partial charges through:

$$\tilde{q}_i^{\alpha} = q_i^{\alpha} + f_i^{\alpha} \cdot l(Q^{\alpha} - \sum_j q_j^{\alpha}), \quad Q^{\alpha} = 0.5(Q - S + 1)$$

where f_i^{α} *is a learned Fukui-like weight ensuring* $\sum_i \tilde{q}_i^{\alpha} = Q^{\alpha}$. *This enables the model to distinguish between different electronic states of the same molecular geometry, which is essential for modelling radical intermediates, homolytic bond cleavage, and open-shell transition states.*

Training data. *AIMNet2 was trained on a large, chemically diverse dataset of organic and elemental-organic molecules spanning 14 elements (H, B, C, N, O, F, Si, P, S, Cl, As, Se, Br, I), with reference calculations performed at the ωB97X-D*[21]*/def2-TZVPP level of theory.*[7] *The training data include neutral and charged species across multiple charge states, with conformations sampled by normal-mode displacement, torsion scanning, and molecular dynamics."*

Response to Reviewer 3:

Recommendation: The considered work by Wang, X. et al. is a high quality systematic study of the value of ML/MM calculations for enzyme design. I have noticed a clear need for additional research in this area and am excited to see this work start the discussion. ML/MM will no doubt play an essential role in the future of enzyme design. However, there is a lack of research showing its limitations and proper implementation protocols. Overall, I find the article to be a good fit for the journal, with thoroughly described methods, and recommend it for publication. However, there are several numerical and citation inconsistencies that should be addressed before publication.

Question 1. There is a slight discrepancy in the claim "Our ML/MM MetaD simulations reproduced these values with sub-kcal mol−1 accuracy on average" on line 244. In Table S3, the ΔG_endo is 26.69 and the ΔG_exo is 27.88, but the ΔG_endo: ΔG_exo ratio is listed as 1:6.4, which is the opposite of what the PMF indicates. Similarly, the Computational ΔΔG is listed as 1.19 where it should be −1.19. Most likely possibility is that the values in the table were swapped. If they are not swapped the main text will need to be updated in a few places to reflect this outlier in the trend.

Response: We thank the reviewer for careful reading. The reviewer is correct: the computational ratio and ΔΔG sign for MaDA3-Mu3 were indeed reported incorrectly in the original Table S3 due to an error in our post-processing. The underlying free-energy values ($\Delta G\_endo = 26.69$ kcal mol^{-1}, $\Delta G\_exo = 27.88$ kcal mol^{-1}) are correct and were not swapped. From these values: $\Delta\Delta G = \Delta G\_endo - \Delta G\_exo = 26.69 - 27.88 = -1.19$ kcal mol^{-1} The negative sign indicates that the endo pathway is kinetically favoured for MaDA3-Mu3. Through the Eyring equation, the corresponding product ratio is: endo:exo = $\exp(-\Delta\Delta G/RT) = \exp(1.19 / (0.001987 \times 323)) \approx 6.4 : 1$ We have corrected Table S3 as follows:

With this correction, MaDA3-Mu3 is the one case among the four mutants where the computational prediction deviates from the experimental trend: experiment reports an approximately 1:1 endo:exo ratio ($\Delta\Delta G \approx 0$), whereas our ML/MM MetaD predicts a clear endo preference ($\Delta\Delta G = -1.19$ kcal mol^{-1}). We have revised the main text to acknowledge this outlier explicitly and to present an updated assessment of the overall accuracy. Across the four MaDA3 mutants, the mean unsigned error (MUE) in ΔΔG is 0.61 kcal mol^{-1}, and the computational predictions correctly capture the qualitative trend for three of the four variants (R294G, R294A, and Mu5). The Mu3 outlier, while outside the sub-kcal mol^{-1} threshold for this particular variant, still falls within the accepted uncertainty range of state-of-the-art free-energy simulations (1 − 2 kcal mol^{-1}). We note that accumulation of five simultaneous mutations in Mu3 (I292F, R294G, L296R) substantially reorganises the active-site environment, which may challenge sampling convergence in the metadynamics protocol. The revised main text now reads (changes underlined in the manuscript):

The correction of the Table S6:

Table S6: Endo/Exo selectivity ratios and activation free energies ($\Delta G^{\ddagger}$, kcal mol^{-1}) of MaDA3 variants from experiment and ML/MM metadynamics simulations. The ratio refers to the relative endo:exo product distribution. $\Delta\Delta G = \Delta G_{\mathrm{endo}} - \Delta G_{\mathrm{exo}}$, with positive values favoring the *exo* pathway.

	MaDA3-R294G	MaDA3-R294A	MaDA3-Mu3	MaDA3-Mu5
Experimental ratio (endo:exo)	1:3.8	1:1.2	1:1	1:0.5
Computational ratio (endo:exo)	1:10.6	1:1.38	6.4:1	1:1.36
ΔG_{endo} (kcal mol^{-1})	27.27	26.88	26.69	27.50
ΔG_{exo} (kcal mol^{-1})	25.87	26.69	27.88	27.30
Experimental $\Delta\Delta G$ (kcal mol^{-1})	0.86	0.11	0	−0.45
Computational $\Delta\Delta G$ (kcal mol^{-1})	1.40	0.19	-1.19	0.20

The corresponding discussions of the MU3 outlier has been added to the Results section from Page 16, Line 282 to Line 288:

"Our simulations reproduced these values with a mean unsigned error of 0.61 kcal mol^{-1} in ΔΔG across the four MaDA-3 variants (Table S6). Three of the four mutants were predicted correctly in both sign and magnitude; the exception was Mu3, for which the simulation predicted an endo preference (ΔΔG = 1.19 kcal mol−1) whereas experiment indicates roughly equal product formation (ΔΔG ≈ 0). This deviation, while outside the sub-kcal mol^{-1} threshold, remains within the typical uncertainty of free-energy simulations."

Question 2. The claim "absolute values exhibited a positive offset about 5 kcal mol−1 compared to theozyme and experimental results" is not supported by the data in Table S2. MaDA3 exo the ΔG‡ is 19.29 experimentally and 25.75 computationally. Thus, an offset of 6.46. And for MaDA1 endo ΔG‡ is 16.14 experimentally and 25.08 computationally. Thus, an offset of 8.94. What I believed happened is the authors used the QM cluster model offset, which is indeed close to 5 so it would be find to update the main text statement to mention the DFT instead of the experimental values or update the offset

Response: We thank the reviewer for this careful check. The reviewer is correct that the offset relative to experimental values is 6-9 kcal/mol, not "about 5." The "about 5" statement was intended to refer to the offset relative to DFT cluster model results: MaDA3 exo: 25.75 − 20.8 = 4.95 kcal/mol; MaDA1 endo: 25.08 − 20.3 = 4.78 kcal/mol.

The corresponding corrections to the discussions has been added from Page 10, Line 184 to Line 187:

"though absolute values obtained with ANI-1xnr exhibited a positive offset of approximately 5 kcal mol^{-1} compared to DFT cluster model results (and approximately 7 – 9 kcal mol−1 compared to experimental values, which themselves differ from DFT estimates by 1 – 4 kcal mol−1 [1,2])."

Question 3. There seems to be an inconsistency in the statement "the endo pathway is thermodynamically preferred. In this case, our method yields ΔΔG = 2.63 kcal mol−1, in close agreement with the DFT estimate of 2.8 kcal mol−1". The statement exerts that the

endo pathway is preferred but a ΔΔG of +2.63 would indicate a higher barrier for the endo pathway based on the definition of ΔΔG in Table S3, ΔΔG = ΔGendo − ΔGexo. Looking at Table S2, it should be negative, endo – exo.

Response: We thank the reviewer for carefully identifying this sign error. Upon re-examination of Table S2, the correct values are: $\Delta G_{endo\ (aqueous)}$ = 27.68 kcal/mol, $\Delta G_{exo\ (aqueous)}$ = 30.31 kcal/mol ΔΔG = ΔG_{endo} - ΔG_{exo} = 27.68 - 30.31 = -2.63 kcal/mol
The negative value correctly reflects that the endo pathway proceeds over a lower activation barrier and is therefore kinetically favored, consistent with the experimental observations. We apologize for any confusion this inconsistency may have caused. The sign error in the original manuscript has been corrected in the revised version:

The corresponding corrections to the discussions has been added from Page 12, Line 209 to Line 211:
"In this case, our method yields ΔΔG = −2.63 kcal mol−1, in close agreement with the DFT estimate of -2.8 kcal mol^{-1}."
Question 4. There is an arithmetic error in Figure 2e, where the |d1 − d2| is computed as 0.49 where It should be 0.39. Similarly, the d3 value is not labeled on the molecular structures on the Figure 2e and 2d plots.

Response: We thank the reviewer for catching this arithmetic error. For MaDA-3 endo (Figure 2e): d1 = 1.73, d2 = 2.12 and |d1 − d2| = |1.73 − 2.12| = 0.39 Å (not 0.49 as printed) We have corrected the figure and also verified all other $|d_1 - d_2|$ values to make sure everything is correct.

We note that for the other four systems, the two phenyl rings adopt a nearly parallel orientation (dihedral angle < 10°), allowing the centroid–centroid distance $d_3$to be labeled directly and unambiguously on the molecular structures. In Figures 2d and 2e, however, the two rings are approximately perpendicular to one another, and placing a d_3 label directly on the structure would obscure key functional-group distances that are more critical for interpreting the reaction geometry. We have therefore chosen to report the d_3 values in the figure caption rather than annotating them directly on the molecular structures in these two cases.

The corresponding corrections have been made to Figure 2:

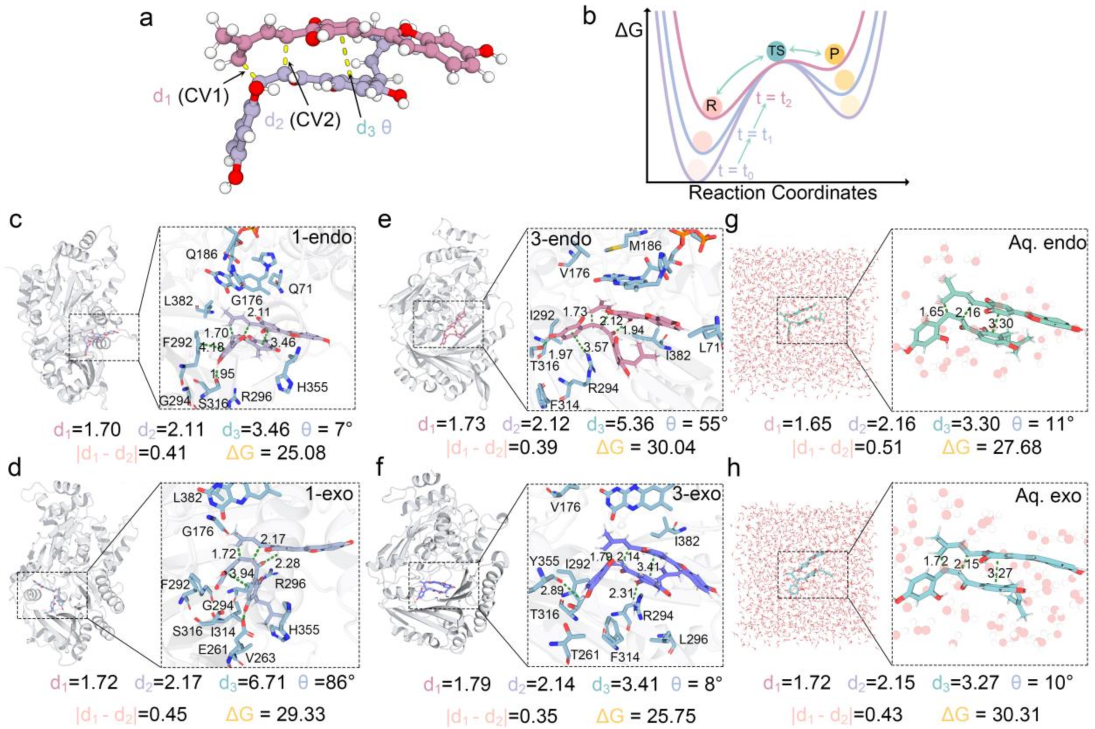

Question 5. The authors have been meticulous in their citations hygiene. However, there are two places that could lead to confusion. In Table S2, DFT QM cluster model data is provided with no attributing citations. While there are citations in the main text, someone reviewing only the SI may attribute this data to this publication. Similarly, in Figure 4, experimental values from different publications are provided with no accompanying citations in the figure or caption. While there are citations in the main text, sometimes figures are shown separately from the text during presentations/reviews which could lead to misattribution of the data.

Response: We thank the reviewer for this important point regarding citation hygiene. We have added explicit citations directly to Table S2, noting that the DFT QM cluster model data are taken from other publications. For Figure 4, we have added reference citations to the caption alongside all experimental values, ensuring that the source of each data point is unambiguous. This ensures that both the table and figure can be interpreted independently of the main text, avoiding any potential misattribution.

Table S2 now has been marked as Table S5 and corresponding citations have been added to Table S5:

Table S5: Activation free energies ($\Delta G^{\ddagger}$, kcal mol^{-1}) of MaDA1 and MaDA3 along the *endo*/*exo* pathways, as obtained from experiment, DFT cluster models, and ML/MM metadynamics. The symbol / denotes cases where no previous experimental or computational data were available. DFT QM cluster model data from Refs.[27,28] All energies are reported in kcal mol^{-1}.

	MaDA3 *endo*	MaDA3 *exo*	MaDA1 *endo*	MaDA1 *exo*	Aqueous *endo*	Aqueous *exo*
Experimental	/	19.29	16.14	/	/	/
DFT cluster	/	20.8	20.3	/	23.3	26.1
ML/MM MetaD (ANI-1xnr)	30.04	25.75	25.08	29.33	27.68	30.31
ML/MM MetaD (AIMNet2-NSE)	22.72	23.13	23.72	27.01	23.76	27.91

The corresponding citations have been added to Figure 4:

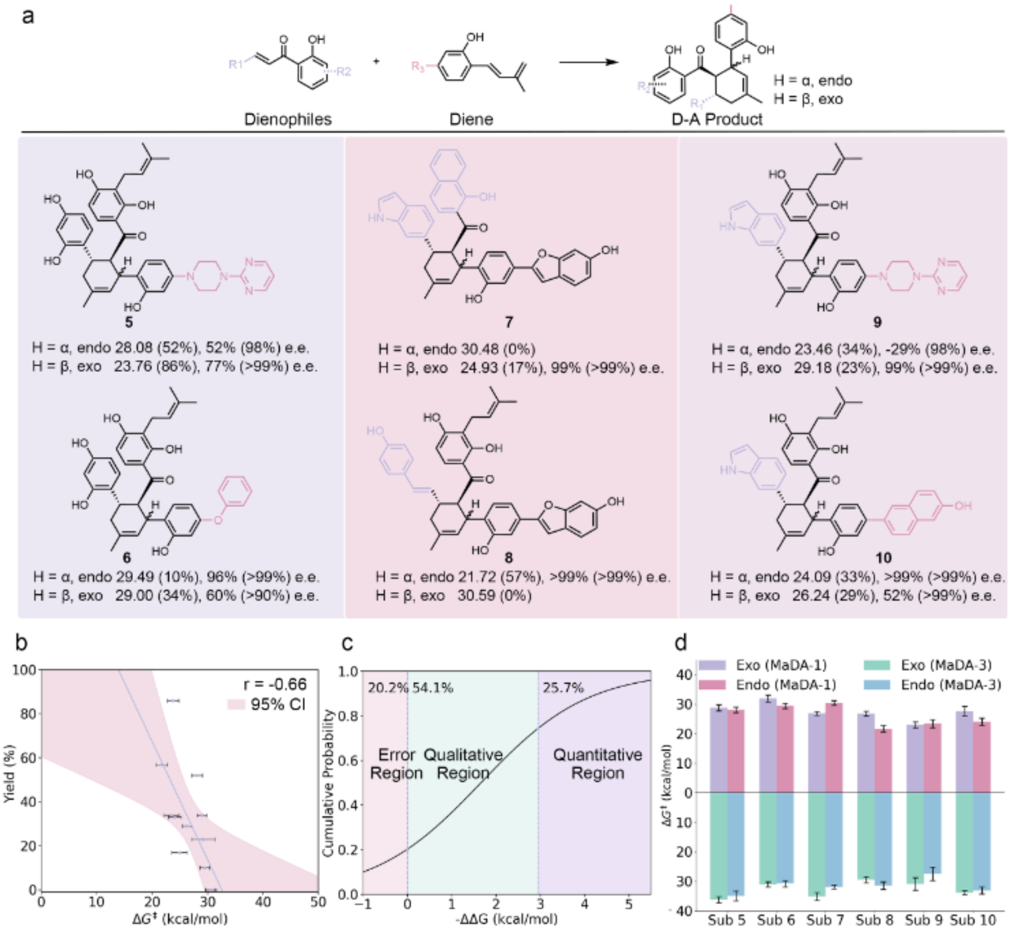

Figure 4: Generalizability of ML/MM across a broad catalytic substrate spectrum. **a**, Estimated activation-energy barriers (in kcal mol^{-1}) and corresponding enantiomeric excesses (e.e.) obtained from ML/MM simulations; experimental yields and e.e. values[1,2] are shown in brackets. **b**, Linear regression of experimental yieldsversus estimated $\Delta G^{\ddagger}$, with data points shown as dots and error bars representing the standard error. The absolute Pearson correlation coefficient (r) and the 95% confidence interval (CI) are indicated. **c**, Analysis and empirical cumulative distribution function fitting of the $-\Delta\Delta G$ distribution. The error region denotes cases where the predominant enantiomer is incorrectly assigned ($-\Delta\Delta G < 0$); the qualitative region corresponds to correct enantiomer prediction with $> 1\%$ deviation in e.e. relative to experiment; and the quantitative region indicates both correct enantiomer assignment and quantitative agreement in e.e. values. **d**, Computed activation-energy barriers (in kcal mol^{-1}) in MaDA-1 and MaDA-3 for the two enantiomeric pathways.

Question 6. In the sentence, "To balance accuracy and efficiency, Walsh et al. introduced the hybrid QM/MM framework" the authors attribute the discovery to Walsh whereas it should be to Warshel. Levitt could also be mentioned.

Response: We sincerely apologize for this error. The QM/MM framework was introduced by and Levitt in their seminal 1976 paper (J. Mol. Biol. 1976, 103, 227–249), for which Warshel shared the 2013 Nobel Prize in Chemistry.

The corresponding corrections have been made in Introduction from Page 4, Line 38 to Line 39:

"To balance accuracy and efficiency, Warshel and Levitt introduced the hybrid QM/MM framework[9]*"*

Question 7. The authors claim "the link-atom method (Fig. 1c) remains the simplest and most practical choice, yet it has never been systematically implemented in ML/MM simulations of enzymatic reactivity". I would remove this claim as a simple search returns a few previous demonstrations of ML/MM link atom demonstrations (e.g., ML/MM toolkit – Towards Accelerated Mechanistic Investigation of Enzymatic Reactions)

Response: We thank the reviewer for this important correction. Following the reviewer's suggestion, we conducted a more thorough literature search and identified the ML/MM Toolkit paper (Ohmura et al., ChemRxiv 2025) as a prior implementation of the link-atom approach in an ML/MM context for enzymatic reactions. We agree that our original claim was inaccurate and have removed it entirely from the revised manuscript.

In its place, we have added a more careful and accurate statement that acknowledges prior work while clarifying our own contribution. We now note that, to the best of our knowledge, only a small number of implementations have demonstrated link-atom ML/MM for enzymatic reactivity, and that the field as a whole remains in an early stage. We have also substantially expanded the surrounding discussion to provide a more complete description of the link-atom method itself, including the physical motivation for capping dangling valences, the deterministic regeneration of the capping atom at each MD step, and the Jacobian-based force redistribution scheme, as well as a broader account of the factors that have limited the adoption of ML/MM beyond substrate-only treatments, of which the boundary scheme is only one aspect. We hope these revisions address the reviewer's concern and more accurately represent the current state of the field.

The corresponding correction and more comprehensive discussion of link-atom scheme have been added to Introduction from Page 4, Line 56 to Page 5, Line 70:

"Although the ML/MM framework is now well established for non-reactive simulations, extending it to chemical reactions has required overcoming two interrelated challenges. One of the primary bottleneck is the availability of robust, transferable reactive MLIPs; recent models such as ANI-1xnr[26] *and AIMNet2-rxn*[27] *have largely addressed this gap, enabling ML/MM to model bond-breaking and bond-forming events. The second challenge is the ML/MM boundary: without*

a boundary treatment that can cap severed covalent bonds, the ML region must be confined to an intact molecule, typically the substrate. Indeed, current reactive ML/MM studies of enzymatic reactions have been limited to substrate-only ML regions[28]*, restricting applications to reaction types. To overcome this limitation, we draw on decades of QM/MM experience with boundary schemes including hybrid orbitals*[29]*, buffer methods*[30]*, and pseudobonds*[31]*, and adopt the link-atom method*[32] *(Fig. 1c), the most widely used approach*[33]*, to incorporate active-site residues into the ML region and thereby extend reactive ML/MM to the full breadth of enzymatic mechanisms. In the link-atom scheme, a hydrogen capping atom is inserted along each severed covalent bond at every MD step, saturating the dangling valence of the boundary atom and providing the MLIP descriptor with a chemically complete local environment."*

Response Letter

We sincerely appreciate the reviewers' comments and suggestions, which have greatly improved the paper. Below are our responses. The italicized sections correspond to new/updated discussions, which are also highlighted in the revised manuscript.

Response to Reviewer 1:

Recommendations: I thank the authors for their thorough and thoughtful revision. The manuscript has been substantially improved in response to all three reviewers' comments. I particularly appreciated the ML region convergence study, which provides a pragmatic and convincing response to the mechanical embedding limitation: by systematically expanding the ML region to include successive shells of active-site residues, the authors demonstrate that polarization effects are captured directly within the MLIP rather than through the embedding scheme, and that the computed barriers are well-converged beyond the catalysis-related region. This is a sensible strategy that sidesteps the need for electrostatic embedding in the present context. The remaining changes (comprehensive literature review, revised novelty claims, performance benchmarks, etc.) collectively address the other concerns I raised in my initial review. I am now satisfied that the work is suitable for publication in Nature Communications. I have only two minor observations that the authors may wish to consider during the proofing stage:

Question 1. Performance scaling interpretation. The authors state that "throughput is essentially independent of ML region size up to at least 222 atoms, confirming that the MLIP evaluation rather than the boundary treatment dominates the computational cost." I believe this interpretation is slightly inverted: the fact that throughput is flat up to ~222 atoms suggests that the MLIP evaluation is not yet the bottleneck in that regime, and that some other component (e.g., the MM evaluation or the coupling overhead) is rate-limiting. The MLIP cost appears to become relevant only at larger region sizes. This does not affect any conclusion of the paper, but a small rephrasing would be more precise.

Response: We thank the reviewer for this insightful observation. The reviewer's interpretation is correct: in the current ML/MM framework, the MM evaluation dominates the overall computational cost and is therefore the rate-limiting component. Consequently, the addition of a modest number of link atoms has a negligible impact on throughput compared to the MM calculation. We note that this clarification does not affect any of the conclusions presented in the paper.

Question 2. Link atom treatment across architectures. The new SI section "Universal Link Atom Treatment Across Heterogeneous MLIP Architectures" provides a detailed and pedagogically valuable exposition. However, I note that in practice, the MLIP simply receives a capped molecular cluster containing a hydrogen atom at the link position: the architecture-specific descriptions document the normal internal operation of each MLIP when presented with that hydrogen, rather than any modification to the models themselves.

The authors may wish to clarify this distinction so that readers do not overinterpret the complexity of the link atom dispatch as implying architecture-specific algorithmic changes.

Response: We thank the reviewer for this important clarification. The SI section was added in the previous revision with the intention of helping readers understand the mathematical formalism underlying our framework and the supported MLIP architectures. The reviewer's observation is well-taken: in practice, the ML/MM framework simply presents each MLIP with a capped molecular cluster containing a hydrogen atom at the link position, without any architectural modification to the models themselves. To prevent readers from overinterpreting the link atom dispatch as implying architecture-specific algorithmic changes, we have added the following clarifying statement to the SI.

The corresponding clarifying is presented on Supporting Information Page S2:
"In practice, no modification to any MLIP architecture is required: the module simply presents each model with a capped molecular cluster containing a hydrogen link atom, and the architecture-specific behavior described below arises entirely from the native descriptor extraction scheme of each model."

Question 3. SI figure numbering. I noticed a discrepancy between the figure numbers referenced in the main text and those in the Supporting Information (e.g., the ML region convergence figure is referred to as Figure S4 in the main text but labeled Figure S3 in the SI). I would recommend the authors carefully check all SI figure cross-references for consistency before final publication.

Neither point requires additional calculations or a further revision cycle.

Response: We thank the reviewer for carefully identifying this inconsistency. We have thoroughly checked all cross-references between the main text and the Supporting Information, and corrected the figure numbering to ensure full consistency throughout the manuscript. The discrepancy has been resolved in the revised version.

Question 4. The code is hosted on GitHub (https://github.com/ClickFF/MLMM4AMBER) and includes a README file, detailed installation instructions (INSTALL.md), and a usage tutorial with example workflows. I did not install or run the code myself, but the installation procedure is well documented and the primary complexity lies in compiling AMBER itself, which serves as the backend MD engine and is a prerequisite. The authors have addressed the repository documentation concerns raised during the first round of review.

Response: We sincerely thank the reviewer for examining the code repository and documentation. We are committed to making the MLMM framework as accessible as possible to the broader computational chemistry community. To this end, we have ensured that the repository includes comprehensive documentation spanning installation, configuration, and step-by-step tutorials with example workflows. We believe that lowering the barrier to adoption is essential for promoting the wider application of ML/MM methods,

and we hope that this framework will serve as a practical tool to further advance rational enzyme design and mechanistic elucidation in enzymatic catalysis.

Response to Reviewer 2:

Recommendations: The analysis of the ML/MM region is greatly improved and was required. The analysis of ANI-1xnr shows that this is a source of error and better potentials are available. I don't think that stating that when the project started this was the best available potential is a justification for the choice.

Response: We thank the reviewer for this critical assessment regarding the choice of MLIP. We fully agree that ANI-1xnr is not the optimal potential for the systems studied, and we acknowledge that the justification based on its availability at the project's inception is insufficient. Indeed, a central finding of our revised manuscript is the systematic identification of the MLIP as the primary source of error in the ML/MM framework. By replacing ANI-1xnr with the more recent AIMNet2-NSE model, we observed an improvement of 2-3 kcal/mol in computed barriers, bringing the results into closer agreement with experimental values. This comparison serves as a concrete demonstration that the accuracy of ML/MM simulations is critically dependent on the quality of the underlying MLIP, and we believe this constitutes one of the practical contributions of our work: establishing a transparent benchmarking protocol that enables users to evaluate and upgrade MLIPs as better models become available.

We also wish to highlight that the MLIP field is advancing at a remarkable pace. Even during the revision period of this manuscript, several state-of-the-art models have been released, including Meta's UMA 1.2 (Universal Models for Atoms, https://github.com/facebookresearch/fairchem; arXiv:2506.23971), and MACE-POLAR-1 (arXiv:2602.19411). These developments underscore that any study employing MLIPs will inevitably face a degree of model obsolescence given the current pace of innovation. Rather than viewing this as a limitation, we see it as further motivation for the modular design of our framework, which allows straightforward integration of next-generation MLIPs as they emerge. We are committed to incorporating these frontier models in our future studies, and we thank the reviewer for the comments that have helped us articulate this point more clearly.

Response to Reviewer 3:

Reviewer #3 (Remarks to the Author):

Recommendations: I thank the authors for the care they took in addressing my suggestions. I have reviewed the responses and all of my suggestions have been addressed completely.

Response: We sincerely thank the reviewer for the constructive suggestions provided during the review process, which have significantly improved the quality and clarity of our

manuscript. We hope that this work will make a meaningful contribution to the field and inspire further developments in ML/MM methodology for enzyme catalysis.
